# Synthesis and Preliminary Evaluation of the Cytotoxicity of Potential Metabolites of Quinoline Glycoconjugates

**DOI:** 10.3390/molecules27031040

**Published:** 2022-02-03

**Authors:** Monika Domińska, Gabriela Pastuch-Gawołek, Adrian Domiński, Piotr Kurcok, Karol Erfurt

**Affiliations:** 1 Department of Organic Chemistry, Bioorganic Chemistry and Biotechnology, Silesian University of Technology, B. Krzywoustego 4, 44-100 Gliwice, Poland; 2 Biotechnology Centre, Silesian University of Technology, B. Krzywoustego 8, 44-100 Gliwice, Poland; 3 Centre of Polymer and Carbon Materials, Polish Academy of Sciences, M. Curie-Skłodowskiej 34, 41-819 Zabrze, Poland; adrian.dominski@cmpw-pan.edu.pl (A.D.); piotr.kurcok@cmpw-pan.edu.pl (P.K.); 4 Department of Chemical Organic Technology and Petrochemistry, Silesian University of Technology, B. Krzywoustego 4, 44-100 Gliwice, Poland; karol.erfurt@polsl.pl

**Keywords:** quinoline glycoconjugates, metabolites, cytotoxicity, anticancer activity, click chemistry

## Abstract

The design of prodrugs is one of the important strategies for selective anti-cancer therapies. When designing prodrugs, attention is paid to the possibility of their targeting tumor-specific markers such as proteins responsible for glucose uptake. That is why glycoconjugation of biologically active compounds is a frequently used strategy. Glycoconjugates consisting of three basic building blocks: a sugar unit, a linker containing a 1,2,3-triazole ring, and an 8-hydroxyquinoline fragment was described earlier. It is not known whether their cytotoxicity is due to whole glycoconjugates action or their metabolites. To check the biological activity of products that can be released from glycoconjugates under the action of hydrolytic enzymes, the synthetically obtained potential metabolites were tested in vitro for the inhibition of proliferation of HCT-116, MCF-7, and NHDF-Neo cell lines using the MTT assay. Research shows that for the full activity of glycoconjugates, the presence of all three building blocks in the structure of a potential drug is necessary. For selected derivatives, additional tests of targeted drug delivery to tumor cells were carried out using polymer nanocarriers in which they are encapsulated. This approach significantly lowered the determined IC_50_ values of the tested compounds and improved their selectivity and effectiveness.

## 1. Introduction

Designing anticancer drugs is one of the greatest challenges in medicinal chemistry in the XXI century. There are many therapeutic strategies for the treatment of cancer, such as surgery, radiation therapy, and chemotherapy. However, most of the available anti-cancer therapies are characterized by narrow therapeutic windows, which are mainly due to their high systemic toxicity, caused by a lack of tumor-specific selectivity. Therefore, it is necessary to search for new, effective drugs for the treatment of cancer. An important element of this search is the increasingly better understanding of the mechanisms of action of potential drugs, which may result in the implementation of safer, more selective therapies.

One of the many directions offered by medical chemistry is the concept of designing prodrugs, i.e., substances that become active only as a result of their metabolism inside the body. According to the definition proposed 60 years ago, prodrugs are inactive derivatives of drugs that undergo biotransformation in vivo to release active molecules, allowing specific drug delivery and triggering a therapeutic effect. The prodrug strategy minimizes drug deactivation before achieving the expected molecular goal and is the starting point for research into mechanisms that determine the bioavailability of potential drugs [1,2,3]. Most prodrugs are designed to improve the physicochemical, biopharmaceutical, or pharmacokinetic properties of a compound. The main aim is to improve the solubility and bioavailability of the drug and to increase its penetration into the cell. In most cases, prodrugs are simple chemical derivatives that require only one to two chemical or enzymatic transformation steps to yield the active parent drug. In some cases, a prodrug may consist of two pharmacologically active drugs that are coupled together into a single molecule so that each drug acts as a promoiety for the other such derivatives, called co-drugs [4]. A significant amount of the conventional chemotherapeutic agents have poor pharmacokinetic profiles and are distributed non-specifically in the body, leading to systemic toxicity with serious side effects. When designing prodrugs, attention is paid to the possibility of targeting tumor-specific markers. The microenvironment of tumors differs significantly from normal tissues. Compared to healthy counterparts, cancer tissues are characterized by unique pathophysiological markers such as hypoxia or a reducing microenvironment, high intracellular glutathione level, low pH, specific proteins overexpression, and elevated level of reactive oxygen species. All of these factors can act as promoters of prodrugs activation to induce a pharmacological effect without damaging normal tissues [5,6].

Cancer cells are characterized by changed energy metabolism compared to healthy cells. This fact contributes to the Warburg effect and is one of the most common features of cancer. Cancer cells produce their energy through glycolysis followed by lactic acid fermentation, characteristic of hypoxic conditions, and its level is much higher (over a hundred times) than in healthy cells, for which mitochondrial oxidative phosphorylation is the main source of energy [7,8]. The high rate of glycolysis consumes large amounts of glucose, hence the cells of some neoplasms are characterized by overexpression of proteins responsible for glucose uptake into their interior, the so-called GLUT transporters [9,10,11]. This fact can be exploited for the selective delivery of drugs by glycoconjugation of biologically active compounds [12,13]. The glycoconjugate prodrugs aim to reduce the systemic toxicity of the drug by targeted transport to cancer cells via GLUT transporters and the release of the active form of the drug in the intracellular microenvironment.

It is also known that cancer cells have an increased need for metal ions such as zinc, calcium, iron, and copper. They are involved in basic cellular processes, therefore their chelation effect plays an important role in drug design [14,15,16]. The elevated amount of copper in cancerous tissues, combined with the fact that copper promotes angiogenesis, cancer growth, and metastasis, has led to attempts to obtain copper-complexing compounds and use them in anti-cancer therapy [17,18,19]. An example of such a compound is 8-hydroxyquinoline, the derivatives of which constitute one of the important groups of metal-chelating compounds necessary for the growth and angiogenesis of neoplastic cells [20,21,22,23]. The conducted experiments proved that the conjugation of metallodrugs with a molecule of sugar is able to deliver prodrugs to a specific tumor tissue due to the overexpression of glucose receptors in neoplastic cells, which provides better antitumor activity and reduction in systemic toxicity [24,25].

**Figure 1:**
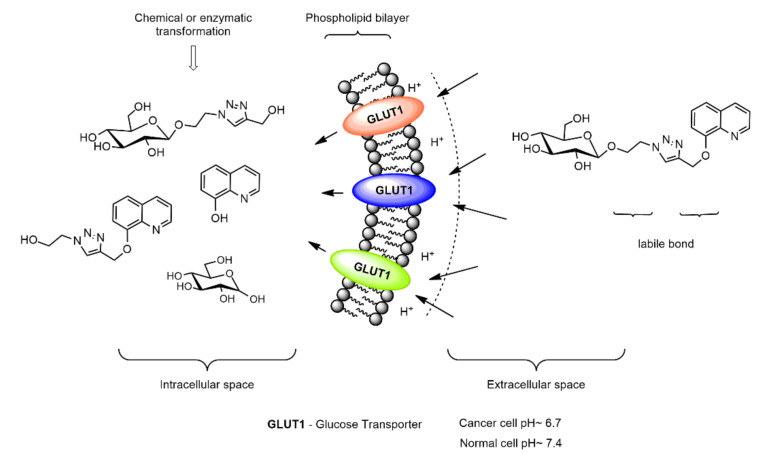
Interaction at the micro-environment. Targeted prodrug delivery.

Considering the above, our research group conducted a series of experiments on the glycoconjugation of 8-hydroxyquinoline (8-HQ) derivatives [26,27,28]. We assumed that due to the addition of a sugar derivative to the active 8-HQ fragment, the obtained molecules would be able to selectively enter a tumor cell using the Warburg effect. Moreover, the addition of the 1,2,3-triazole fragment to the structure of glycoconjugates improved their cytotoxic activity and had a positive effect on the ability to form complexes with metal ions. Several of the designed glycoconjugates derivatives of 8-HQ have shown significant cytotoxicity at the micromolar level against the variety of cell lines tested, compared to their parent compounds. The designed bonds linking sugar and quinoline are stable in the extracellular space. However, after entering the cell, these compounds can be degraded under the action of hydrolytic enzymes, releasing the active form of the drug, which is able to induce cytotoxicity (Figure 1). Except for the cytotoxicity assessment of the glycoconjugates themselves, it seems advisable to check the biological activity of products that can be released in the tumor cell from glycoconjugates by the action of hydrolytic enzymes. Comprehensive research should focus on assessing the activity of each of the possible metabolites of a given prodrug to determine exactly what role individual fragments of the molecule play, as well as which of their structural elements are responsible for the antitumor effect and whether these compounds are not toxic to healthy cells.

## 2. Results and Discussion

### 2.1. Synthesis

The aim of the research was to obtain and evaluate the biological activity of potential metabolites that may be formed in biological systems by the degradation of anti-cancer prodrugs based on glycoconjugates derivatives of 8-hydroxyquinoline described in recent works [26,27,28]. This will allow answering the question of which fragment of the molecule is responsible for the obtained biological activity.

The designed compounds consist of an 8-hydroxyquinoline fragment linked by an aliphatic chain of various lengths to a 1,2,3-triazole ring. The second type of metabolites are D-glucose derivatives containing anomeric oxygen, nitrogen, or sulfur atom and also connect to the 1,2,3-triazole ring by various linkers. Due to the stability of triazoles in typical physiological conditions, the 1,2,3-triazole linker is present in glycoconjugates and all metabolite structures. Studies have shown that the 1,2,3-triazole ring is an important system that influences anti-cancer activity. Furthermore, their tendency to form hydrogen bonds increases the solubility of such molecules in biological systems, which favor binding to biomolecular targets, thus allowing in vivo administration [29].

**Scheme 1:**
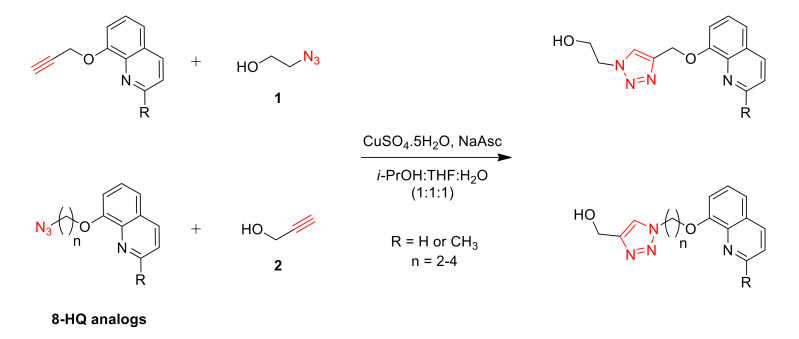
Strategies for the synthesis of metabolites of 8-hydroxyquinoline derivatives.

**Scheme 2:**
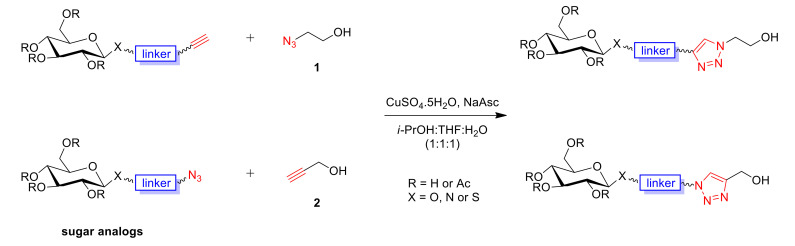
Strategies for the synthesis of metabolites of sugar derivatives.

**Figure 2:**
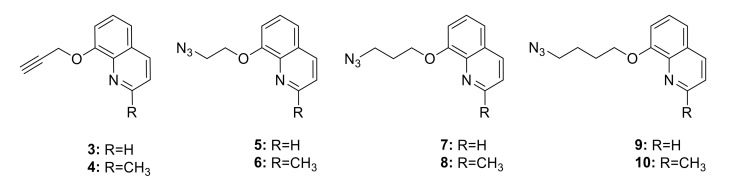
Structures of substrates derivatives of 8-hydroxyquinoline.

**Figure 3:**
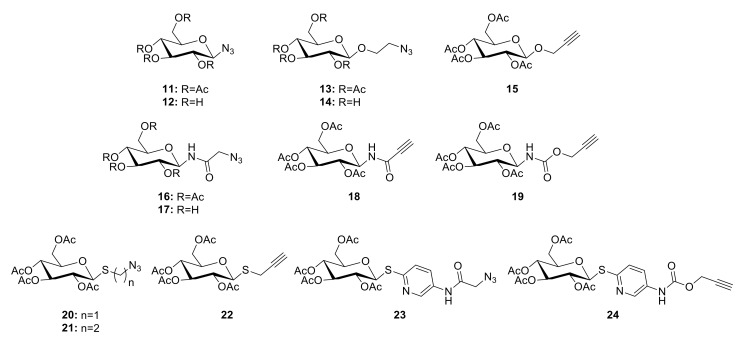
Structures of substrates derivatives of sugar.

The desired metabolites were prepared by the copper(I)-catalyzed 1,3-dipolar azide-alkyne cycloaddition (CuAAC), in the variant used for the synthesis of biologically active compounds belonging to the group of the so-called *click-chemistry* reactions developed by Sharples [29,30,31,32]. This type of reaction allows for the quick and efficient synthesis of new compounds and combinatorial libraries. This process is characterized by high yields, stereospecificity, mild reaction conditions, the absence of by-products, and the simplicity of product purification. The CuAAC reaction was carried out between the appropriate azide or propargyl quinoline derivatives **3**–**10** or sugar derivatives **11**–**24** and 2-azidoethanol **1** or propargyl alcohol **2**. A general procedure for the synthesis of metabolites is shown in Scheme 1 and Scheme 2. Used for the reaction, quinoline substrates functionalized in the 8-OH position (Figure 2) and sugar derivatives substituted at the anomeric position (Figure 3) were prepared according to the previously published procedures [26,27,28]. Propargyl alcohol **2** is a commercially available reagent, whereas 2-azidoethanol **1** was obtained by substituting 2-bromoethanol with sodium azide in DMF [33]. Confirmation of bromine atom exchange to the azide moiety was the appearance in the ^13^C-NMR spectra of the signal of CH_2_N_3_ carbon with a shift of about 53.5 ppm.

The reaction for the synthesis of metabolites was carried out by dissolving the mentioned reagents in an equimolar ratio in the THF/*i*-PrOH solvent system, followed by adding the CuSO_4_·5H_2_O/NaAsc aqueous catalyst system to the reaction mixture. CuSO_4_ was used as the source of copper ions. Meanwhile, sodium ascorbate was a reducing agent of Cu(II) to Cu(I) in situ and avoided the formation of oxidation byproducts. The reaction was carried out for 24 h at room temperature. Pure products were isolated by column chromatography in good or very good yields. As a result of the reaction, structures containing only a 1,4-disubstituted 1,2,3-triazole ring in the linker were obtained. The structures of all synthesized metabolites, as well as the standard glycoconjugates, are presented in Table 1 and Table 2. The new structures were confirmed using NMR and HRMS spectroscopy methods. The presence of characteristic signals in the NMR spectra originating from the 1,2,3-triazole ring indicated the formation of the desired product. These are signal a singlet at about δ = 7.9 ppm from the H-C(5) proton triazole ring in ^1^H-NMR spectra and two characteristic carbon signals at about 123 ppm and 144 ppm for C(4) and C(5) from triazole ring in the ^13^C-NMR spectra. The NMR spectra of all synthesized products are presented in the Supplementary Materials. The physicochemical properties, such as melting point and optical rotation, were also determined.

### 2.2. Cytotoxicity Studies

The obtained compounds were tested in vitro for the inhibition of the proliferation of several tumor cell lines. Cell lines were selected for the experiments, on which glycoconjugates from which described metabolites could arise were previously tested. HCT-116 (colorectal carcinoma cell line) and MCF-7 (human breast adenocarcinoma cell line) are characterized by a high demand for glucose and overexpression of the GLUT transporters [34,35]. In addition, an excess of copper ions was observed near the above-mentioned lines [36,37]. NHDF-Neo (Normal Human Dermal Fibroblasts-Neonatal) is a healthy cell line and is a reference point to evaluate the selectivity of compounds. Screening of cell viability after exposure to the compounds was performed using the MTT assay. It is used to measure cellular metabolic activity as an indicator of cell viability, proliferation, and cytotoxicity. The basis of the MTT method is the ability of mitochondrial dehydrogenase present in the mitochondria of metabolically active cells to convert the yellow tetrazolium salt (MTT) into purple formazan crystals. MTT assay was carried out according to the protocol (MTT, Sigma-Aldrich, Taufkirchen, Germany) [38]. Cells were treated with various concentrations of test compounds for 24–72 h. The activity of test compounds is expressed by IC_50_ values, defined as 50% inhibition of cell growth compared to the untreated control. The results are presented in Table 1 and Table 2 and compared with the activity of glycoconjugates, which could theoretically form the tested potential metabolites.

The motivation for obtaining the metabolites **M1**–**M8** presented in Table 1 was to check whether the addition of a sugar unit is necessary to improve the biological properties of 8-HQ or whether the introduction of the linker containing the 1,2,3-triazole ring alone would be sufficient. In contrast to the glycoconjugates, which were mostly able to inhibit cell proliferation in the concentration range studied, most of the resulting 8-HQ derivative metabolites did not show interesting activity. Considering the influence of the length of the alkyl chain between the quinoline fragment and the 1,2,3-triazole ring, the most active turned out to be metabolites with three or four carbon atoms in the linker. It is probably related to the possibility of the compound penetrating the phospholipid membrane into the cells. The compound with the longest alkyl chain and the 1,2,3-triazole ring is characterized by the highest lipophilicity, which may help in its transport across cell membranes. The observed lower activity of the analogous glycoconjugate than the most active metabolite **M7** probably indicates incomplete hydrolysis of the glycosidic bond in the cell by β-glycosidases, leading to the release of active aglycone, which is able to complex copper(II) ions.

The obtained metabolites containing a sugar part and a linker with a 1,2,3-triazole ring in the structure were also weakly active or completely inactive against the tested tumor cell lines (Table 2). This confirms that the quinoline fragment is responsible for showing antiproliferative activity. On the other hand, sugar molecules are also an additional source of energy, allowing the proliferation of cells. The exceptions are the compounds **M14** and **M18**. Probably the combination of the 1,2,3-triazole ring with an amide moiety, as in the case of the **M14** compound, improves the ability to complex Cu^2+^ ions, thus inhibiting the proliferation of neoplastic cells by eliminating an important factor of their growth. It is worth adding that the sugar derivative **16** without the 1,2,3-triazole ring was completely inactive against the tested cell lines. Due to the presence of acetyl protecting groups in the sugar part, which improve the lipophilicity of the molecule, the metabolite **M14** may be able to cross biological barriers and enter cells by passive diffusion. The analogous metabolite **M15** with the unprotected sugar fragment showed no antiproliferative activity. The deprotected derivatives apparently do not have sufficient affinity for GLUT transporters. Cytotoxic activity was also demonstrated by the metabolite **M18** containing a sulfur atom in the anomeric position of the sugar. Probably the compounds with the *S*-glycosidic bond do not undergo premature enzymatic degradation before entering the cell and therefore the sulfur atom together with the 1,2,3-triazole ring is able to complex copper ions. It was observed that the extension of the alkyl chain in the metabolite **M19** did not improve the cytotoxicity. On the other hand, the spatial orientation of the 1,2,3-triazole ring with the linker turned out to be important. For comparison, in order to create a 1,2,3-triazole system in the structure of the metabolite **M20**, a derivative of 1-thiosugar with an anomeric propargyl moiety was used. This reaction created an "inverted" system of 1,2,3-triazole in relation to that in the **M18** metabolite. This compound did not show any antiproliferative activity. It can be assumed that not only the hydrolytic stability is important for biological activity, but also the mutual spatial orientation of the atom in the anomeric position of the sugar and the 1,2,3-triazole ring. In view of the above results, the lack of activity of the **M21** and **M22** derivatives is surprising. Quite unexpectedly, no beneficial effect was found to introduce an additional pyridine fragment together with an amide or carbamate bond into the linker structure between the 1-thiosugar moiety and the 1,2,3 triazole ring. This additional fragment was supposed to increase the ability of metabolites to complex copper ions, which are needed for the growth of cancer cells. Perhaps additional cytotoxicity studies on an extended panel of tumor cell lines are worth carrying out for these molecules.

Compounds showing activity against the tested tumor cell lines turned out to be toxic to healthy cells at the same time. This is because passive transport, which allows them to enter the cell, is not preferred for designed prodrugs and does not guarantee selectivity. As a result, the tested compounds damage both cancer cells and healthy cells. Experiments should be extended in the direction allowing for better matching of compounds to the structure of GLUT transporters, which should ultimately improve their selectivity.

The above experiments do not explain whether the low cytotoxic activity of the obtained metabolites results from the real lack of activity of the structures or because of the difficulties crossing of biological membranes by 8-HQ derivatives. To investigate this issue in detail, additional targeted drug delivery experiments to tumor cells were required. One of the strategies to increase the bioavailability and selectivity of drugs is the use of polymeric carriers in which they are encapsulated. Their action is based on the controlled release of encapsulated drugs under the influence of appropriate intracellular stimuli (e.g., temperature, pH), thus improving the drug's ability to target cancer cells [39,40]. It is known that in the microenvironment of neoplastic tissues there is a mildly acidic environment, which results from excessive glycolysis in tumors [41]. Therefore, for the targeted transport of selected, previously tested, metabolites **M5** and **M7**, specially developed biodegradable pH-sensitive polymer nanocarriers were used, which decompose with the release of the drug under the influence of pH changes [42]. Herein, pH-responsive micelles consisting of poly(ethylene glycol)-*b*-poly(acetal-functionalized aliphatic carbonate)-*b*-oligo([R]-3-hydroxybutyrate) triblock copolymer were used as nanocarriers for metabolites. Recent studies have shown that the encapsulation of 8-HQ derivative glycoconjugates in pH-sensitive nanocarriers significantly improved their effectiveness by avoiding systemic side effects and reducing the doses of administered prodrugs [42]. 

**Figure 4:**
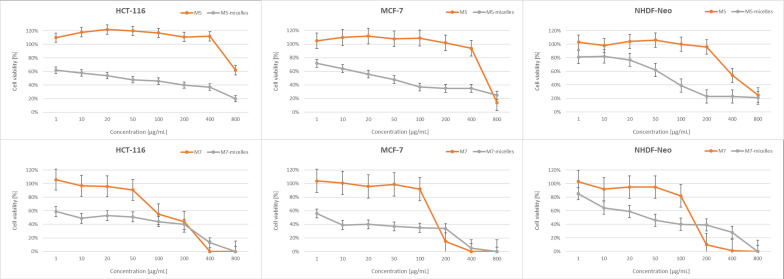
The cell viability of free metabolites and metabolites loaded into micelles for HCT-116, MCF-7, and NHDF-Neo cells after 72 h of incubation. Data are presented as the mean ± standard deviation (*n* = 3).

The in vitro cytotoxic activity of free drugs (**M5** and **M7**) and drug-loaded pH-responsive micelles (**M5**-micelles and **M7**-micelles) was determined by the MTT test. Cells were incubated with the appropriate compounds for 72 h in a concentration range oscillating within the IC_50_ activity of the above-mentioned compounds. The polymeric material can be safely used in research because it is not toxic to the cell lines tested [42]. The relationship between cell viability and the concentration of free drugs and drug-loaded micelles is shown in Figure 4. The IC_50_ value was also determined for each case and is summarized in Table 3. The results demonstrate that the drug-loaded micelles showed a much stronger cytotoxic effect against all tested cell lines as compared to the free drugs. **M5** and **M7** loaded into micelles achieve much lower IC_50_ values compared to their free counterparts. It is worth noting that the inhibitory effect of micelles loaded with metabolites on the proliferation of healthy NHDF-Neo cells, in comparison to free drugs, is not as great as in case wherein they are applied to neoplastic cells. As a result, metabolites-loaded micelles showed a higher selectivity index compared to free metabolites, which may be due to the specific microenvironment of tumor cells. It can be assumed that the use of micelles facilitates the active transport of compounds directly to the cancer cell, thus increasing the accumulation of the drug in cancer cells and its effectiveness. The lower cytotoxic activity of free metabolites as well as of free glycoconjugates is probably due to their difficult penetration into the cell through phospholipid membranes. The low activity of analogous glycoconjugates may also be caused by too early hydrolysis of the glycosidic linkage and therefore difficult crossing of biological membranes, or by an incompatibility between the sugar derivatives and GLUT transporters. However, no mechanism of action can be ruled out at this stage.

## 3. Materials and Methods

### 3.1. General Information

NMR spectra were recorded with an Agilent spectrometer at a frequency of 400 MHz or Varian spectrometer at a frequency of 600 MHz using tetramethylsilane (TMS) as the internal standard and CDCl_3_ or DMSO-d6 as the solvents, which were purchased from ACROS Organics (Geel, Belgium). Chemical shifts (δ) were reported in ppm and the coupling constants (*J*) in Hz. The following abbreviations were used to explain the observed multiplicities: s: singlet, d: doublet, dd: doublet of doublets, ddd: doublet of doublet of doublets, t: triplet, dd~t: doublet of doublets resembling a triplet (with similar values of coupling constants), m: multiplet, b: broad. High-resolution mass spectra (HRMS) were recorded with a WATERS LCT Premier XE system using the electrospray-ionization (ESI) technique. Optical rotations were measured with a JASCO P-2000 polarimeter using a sodium lamp (589.3 nm) at room temperature. Melting point measurements were performed on OptiMelt (MPA 100) Stanford Research Systems. Reactions were monitored by thin-layer chromatography (TLC) on precoated plates of silica gel 60 F254 (Merck Millipore, Burlington, MA, USA). The TLC plates were visualized under UV light (λ = 254 nm) or by charring after spraying with 10% solution of sulfuric acid in ethanol. Crude products were purified using column chromatography performed on Silica Gel 60 (70–230 mesh, Fluka, St. Louis, MI, USA), developed with toluene:EtOAc or CHCl_3_:MeOH as solvent systems. Organic solvents were evaporated on a rotary evaporator under diminished pressure at 40 °C. The absorbance on the MTT assay was measured spectrophotometrically at the 570 nm wavelength using a plate reader (Epoch, BioTek, Winooski, VT, USA).

All used chemicals and solvents were purchased from Sigma-Aldrich (Saint Louis, MO, USA), ACROS Organics (Geel, Belgium), and Avantor Performance Materials (Gliwice, Poland) and were used without further purification. d-Glucose, 8-hydroxyquinoline, 8-hydroxyquinaldine, and propargyl alcohol **2** are commercially available (Sigma-Aldrich). 2-Azidoethanol **1**, 8-Hydroxyquinoline derivatives **3**–**10** and sugar derivatives **11**–**24** were prepared according to the respective published procedures [26,27,28,33].

### 3.2. Chemistry

#### 3.2.1. General Procedure for the Synthesis of Metabolites **M1**–**M22**

The appropriate 8-hydroxyquinoline derivative **3**–**10** or sugar derivative **11**–**24** (1 eq.) was dissolved in dry THF (2 mL) and *i*-PrOH (2 mL) and then 2-azidoethanol **1** or propargyl alcohol **2** (1 eq.) was added. The solutions of sodium ascorbate (0.4 eq.) in H_2_O (1 mL) and CuSO_4_·5H_2_O (0.2 eq.) in H_2_O (1 mL) were mixed and immediately added to the reaction mixture. The reaction was stirred at room temperature for 24 h. The reaction progress was monitored by TLC in an eluents system CHCl_3_:MeOH (20:1 or 2:1). After completion of the reaction, the catalyst systems were filtered off and solvents were concentrated under reduced pressure. The crude residues were purified using column chromatography (toluene:AcOEt, 2:1 and CHCl_3_:MeOH, 100:1 for fully protected glycoconjugates and analogs of 8-HQ or gradient of CHCl_3_:MeOH from 50:1 to 2:1 for products with unprotected sugar part).

1-Hydroxyethyl-4-(8-quinolinyloxymethyl)-*1H*-1,2,3-triazol **M1**: Starting from 8-(prop-2-yn-1-yloxy)quinoline **3** and 2-azidoethanol **1**, the product was obtained as a brown solid (81% yield); m.p.: 126–128 °C; [α]^25^_D_ = −0.6 (c = 1.0, CHCl_3_); ^1^H NMR (400 MHz, CDCl_3_): δ 4.01 (t, 2H, *J* = 5.0 Hz, CH_2_), 4.45 (t, 2H, *J* = 5.0 Hz, CH_2_), 5.46 (s, 2H, CH_2_O_Quin_), 7.28 (dd, 1H, *J* = 1.4 Hz, *J* = 8.2 Hz, H-7_Quin_), 7.35–7.48 (m, 3H, H-3_Quin_, H-5_Quin_, H-6_Quin_), 7.94 (s, 1H, H-5_Triaz_), 8.12 (d, 1H, *J* = 8.3 Hz, H-4_Quin_), 8.83 (bs, 1H, H-2_Quin_); ^13^C NMR (100 MHz, CDCl_3_): δ 52.82 (CH_2_N), 61.02 (CH_2_O), 62.75 (CH_2_O), 109.78 (C-7_Quin_), 120.19 (C-5_Quin_), 121.68 (C-3_Quin_), 124.65 (C-5_Triaz_), 126.82 (C-6_Quin_), 129.50 (C-4a_Quin_), 136.19 (C-4_Quin_), 140.00 (C-8a_Quin_), 143.72 (C-4_Triaz_), 149.12 (C-2_Quin_), 153.76 (C-8_Quin_); HRMS (ESI-TOF): calcd for C_14_H_15_N_4_O_2_ ([M + H]^+^): *m*/*z* 271.1195; found: *m*/*z* 271.1199.

1-Hydroxyethyl-4-(2-methyl-8-quinolinyloxymethyl)-1*H*-1,2,3-triazol **M2**: Starting from 2-methyl-8-(prop-2-yn-1-yloxy)quinoline **4** and 2-azidoethanol **1**, the product was obtained as a brown solid (80% yield); m.p.: 109–111 °C; [α]^25^_D_ = 0.2 (c = 1.0, CHCl_3_); ^1^H-NMR (600 MHz, CDCl_3_): δ 2.73 (s, 3H, CH_3_), 3.99 (t, 2H, *J* = 5.0 Hz, CH_2_), 4.44 (t, 2H, *J* = 5.0 Hz, CH_2_), 5.46 (s, 2H, CH_2_O_Quin_), 7.24 (m, 1H, H-7_Quin_), 7.29 (d, 1H, *J* = 8.4 Hz, H-3_Quin_), 7.34–7.38 (m, 2H, H-5_chin_, H-6_Quin_), 7.90 (s, 1H, H-5_Triaz_), 8.00 (d, 1H, *J* = 8.4 Hz, H-4_Quin_); ^13^C-NMR (150 MHz, CDCl_3_): δ 25.40 (CH_3_), 52.76 (CH_2_N), 61.01 (CH_2_O), 63.01 (CH_2_O), 110.24 (C-7_Quin_), 120.16 (C-5_Quin_), 122.67 (C-3_Quin_), 124.52 (C-5_Triaz_), 125.74 (C-6_Quin_), 127.73 (C-4a_Quin_), 136.28 (C-4_Quin_), 139.65 (C-8a_Quin_), 144.00 (C-4_Triaz_), 153.30 (C-2_Quin_), 158.20 (C-8_Quin_); HRMS (ESI-TOF): calcd for C_15_H_17_N_4_O_2_ ([M + H]^+^): *m/z* 285.1352; found: *m/z* 285.1357.

4-Hydroxymethyl-1-(8-quinolinyloxyethyl)-1*H*-1,2,3-triazol **M3**: Starting from 8-(2-azidoethoxy)quinoline **5** and propargyl alcohol **2**, the product was obtained as a brown solid (91% yield); m.p.: 116–119 °C; [α]^25^_D_ = 23.0 (c = 1.0, CHCl_3_); ^1^H-NMR (400 MHz, DMSO): δ 4.51 (s, 2H, CH_2_OH), 4.61 (t, 2H, *J* = 5.1 Hz, CH_2_), 4.89 (t, 2H, *J* = 5.1 Hz, CH_2_), 7.25 (dd, 1H, *J* = 1.5 Hz, *J* = 7.5 Hz, H-7_Quin_), 7.47–7.58 (m, 3H, H-3_Quin_, H-5_Quin_, H-6_Quin_), 8.25 (s, 1H, H-5_Triaz_), 8.33 (dd, 1H, *J* = 1.9 Hz, *J* = 8.6 Hz, H-4_Quin_), 8.89 (dd, 1H, *J* = 1.7 Hz, *J* = 4.1 Hz, H-2_Quin_); ^13^C-NMR (100 MHz, DMSO): δ 49.02 (CH_2_N), 55.04 (CH_2_O), 67.37 (CH_2_O), 110.39 (C-7_Quin_), 120.43 (C-5_Quin_), 121.90 (C-3_Quin_), 123.32 (C-5_Triaz_), 126.70 (C-6_Quin_), 129.07 (C-4a_Quin_), 135.82 (C-4_Quin_), 139.70 (C-8a_Quin_), 148.07 (C-4_Triaz_), 149.21 (C-2_Quin_), 153.71 (C-8_Quin_); HRMS (ESI-TOF): calcd for C_14_H_15_N_4_O_2_ ([M + H]^+^): *m*/*z* 271.1195; found: *m*/*z* 271.1197.

4-Hydroxymethyl-1-(2-methyl-8-quinolinyloxyethyl)-*1H*-1,2,3-triazol **M4**: Starting from 2-methyl-8-(2-azidoethoxy)quinoline **6** and propargyl alcohol **2**, the product was obtained as a beige solid (83% yield); m.p.: 150–152 °C; [α]^25^_D_ = −27.0 (c = 1.0, CHCl_3_); ^1^H-NMR (400 MHz, DMSO): δ 2.69 (s, 3H, CH_3_), 4.53 (d, 2H, *J* = 4.2 Hz, CH_2_OH), 4.57 (t, 2H, *J* = 5.1 Hz, CH_2_), 4.88 (t, 2H, *J* = 5.1 Hz, CH_2_), 5.15 (bs, 1H, OH), 7.20 (dd, 1H, *J* = 1.3 Hz, *J* = 7.7 Hz, H-7_Quin_), 7.39–7.46 (m, 2H, H-3_Quin_, H-6_Quin_), 7.50 (dd, 1H, *J* = 1.2 Hz, *J* = 8.2 Hz, H-5_Quin_), 8.20 (d, 1H, *J* = 8.4 Hz, H-4_Quin_); 8.46 (s, 1H, H-5_Triaz_), ^13^C-NMR (100 MHz, DMSO): δ 25.02 (CH_3_), 49.02 (CH_2_N), 55.12 (CH_2_O), 67.54 (CH_2_O), 110.88 (C-7_Quin_), 120.33 (C-5_Quin_), 122.52 (C-3_Quin_), 123.71 (C-5_Triaz_), 125.66 (C-6_Quin_), 127.33 (C-4a_Quin_), 135.99 (C-4_Quin_), 139.26 (C-8a_Quin_), 148.08 (C-4_Triaz_), 153.23 (C-2_Quin_), 157.55 (C-8_Quin_); HRMS (ESI-TOF): calcd for C_15_H_17_N_4_O_2_ ([M + H]^+^): *m*/*z* 285.1352; found: *m*/*z* 285.1353.

4-Hydroxymethyl-1-(8-quinolinyloxypropyl)-*1H*-1,2,3-triazol **M5**: Starting from 8-(3-azidopropoxy)quinoline **7** and propargyl alcohol **2**, the product was obtained as a brown solid (86% yield); m.p.: 126–127 °C; [α]^25^_D_ = 19.0 (c = 1.0, CHCl_3_); ^1^H-NMR (400 MHz, DMSO): δ 2.41 (p, 2H, *J* = 6.5 Hz, CH_2_), 4.19 (t, 2H, *J* = 6.1 Hz, CH_2_), 4.51 (s, 2H, CH_2_OH), 4.62 (t, 2H, *J* = 6.9 Hz, CH_2_), 7.19 (dd, 1H, *J* = 2.0 Hz, *J* = 7.0 Hz, H-7_Quin_), 7.47–7.59 (m, 3H, H-3_Quin_, H-5_Quin_, H-6_Quin_), 8.10 (s, 1H, H-5_Triaz_), 8.32 (dd, 1H, *J* = 1.6 Hz, *J* = 8.2 Hz, H-4_Quin_), 8.89 (bs, 1H, H-2_Quin_); ^13^C-NMR (100 MHz, DMSO): δ 29.56 (CH_2_), 46.37 (CH_2_N), 54.97 (CH_2_O), 65.31 (CH_2_O), 109.76 (C-7_Quin_), 119.83 (C-5_Quin_), 121.76 (C-3_Quin_), 122.76 (C-5_Triaz_), 126.70 (C-6_Quin_), 128.97 (C-4a_Quin_), 135.71 (C-4_Quin_), 139.70 (C-8a_Quin_), 147.96 (C-4_Triaz_), 148.95 (C-2_Quin_), 154.13 (C-8_Quin_); HRMS (ESI-TOF): calcd for C_15_H_17_N_4_O_2_ ([M + H]^+^): *m/z* 285.1352; found: *m/z* 285.1354.

4-Hydroxymethyl-1-(2-methyl-8-quinolinyloxypropyl)-1*H*-1,2,3-triazol **M6**: Starting from 2-methyl-8-(3-azidopropoxy)quinoline **8** and propargyl alcohol **2**, the product was obtained as a brown oil (79% yield); [α]^25^_D_ = 3.0 (c = 1.0, CHCl_3_); ^1^H-NMR (400 MHz, DMSO): δ 2.41 (m, 2H, CH_2_), 2.68 (s, 3H, CH_3_), 4.19 (m, 2H, CH_2_), 4.51 (s, 2H, CH_2_OH), 4.63 (m, 2H, CH_2_), 7.18 (m, 1H, H-7_Quin_), 7.36–7.56 (m, 3H, H-3_Quin_, H-5_Quin_, H-6_Quin_), 8.12 (s, 1H, H-5_Triaz_), 8.22 (m, 1H, H-4_Quin_); ^13^C-NMR (100 MHz, DMSO): δ 24.96 (CH_3_), 29.56 (CH_2_), 46.43 (CH_2_N), 55.03 (CH_2_O), 65.58 (CH_2_O), 110.40 (C-7_Quin_), 119.87 (C-5_Quin_), 122.53 (C-3_Quin_), 122.94 (C-5_Triaz_), 125.80 (C-6_Quin_), 128.93 (C-4a_Quin_), 136.05 (C-4_Quin_), 139.10 (C-8a_Quin_), 148.10 (C-4_Triaz_), 153.34 (C-2_Quin_), 158.93 (C-8_Quin_); HRMS (ESI-TOF): calcd for C_16_H_19_N_4_O_2_ ([M + H]^+^): *m*/*z* 299.1508; found: *m*/*z* 299.1509.

4-Hydroxymethyl-1-(8-quinolinyloxybutyl)-1*H*-1,2,3-triazol **M7**: Starting from 8-(4-azidobutoxy)quinoline **9** and propargyl alcohol **2**, the product was obtained as a brown oil (80% yield); [α]^25^_D_ = 1.0 (c = 1.0, CHCl_3_); ^1^H-NMR (400 MHz, DMSO): δ 1.82 (m, 2H, CH_2_), 2.07 (m, 2H, CH_2_), 4.21 (t, 2H, *J* = 5.6 Hz, CH_2_), 4.51 (t, 2H, *J* = 5.6 Hz, CH_2_), 4.52 (s, 2H, CH_2_OH), 7.20 (m, 1H, H-7_Quin_), 7.40–7.66 (m, 3H, H-3_Quin_, H-5_Quin_, H-6_Quin_), 8.12 (s, 1H, H-5_Triaz_), 8.32 (m, 1H, H-4_Quin_), 8.87 (bs, 1H, H-2_Quin_); ^13^C-NMR (100 MHz, DMSO): δ 25.57 (CH_2_), 27.01 (CH_2_), 48.93 (CH_2_N), 55.08 (CH_2_O), 67.85 (CH_2_O), 109.36 (C-7_Quin_), 119.63 (C-5_Quin_), 121.84 (C-3_Quin_), 122.91 (C-5_Triaz_), 126.84 (C-6_Quin_), 128.85 (C-4a_Quin_), 135.75 (C-4_Quin_), 139.75 (C-8a_Quin_), 147.92 (C-4_Triaz_), 148.89 (C-2_Quin_), 154.50 (C-8_Quin_); HRMS (ESI-TOF): calcd for C_16_H_19_N_4_O_2_ ([M + H]^+^): *m/z* 299.1508; found: *m/z* 299.1509.

4-Hydroxymethyl-1-(2-methyl-8-quinolinyloxybutyl)-1*H*-1,2,3-triazol **M8**: Starting from 2-methyl-8-(4-azidobutoxy)quinoline **10** and propargyl alcohol **2**, the product was obtained as a brown oil (76% yield); [α]^24^_D_ = −2.0 (c = 0.9, CHCl_3_); ^1^H-NMR (400 MHz, DMSO): δ 1.80 (m, 2H, CH_2_), 2.07 (m, 2H, CH_2_), 2.64 (s, 3H, CH_3_), 4.20 (m, 2H, CH_2_), 4.48–4.60 (m, 4H, CH_2_, CH_2_OH), 7.16 (m, 1H, H-7_Quin_), 7.34–7.50 (m, 3H, H-3_Quin_, H-5_Quin_, H-6_Quin_), 8.18 (m, 1H, H-4_Quin_); 8.19 (s, 1H, H-5_Triaz_), ^13^C-NMR (100 MHz, DMSO): δ 24.97 (CH_3_), 25.30 (CH_2_), 27.19 (CH_2_), 48.93 (CH_2_N), 55.07 (CH_2_O), 68.11 (CH_2_O), 109.60 (C-7_Quin_), 119.40 (C-5_Quin_), 122.42 (C-3_Quin_), 123.15 (C-5_Triaz_), 125.74 (C-6_Quin_), 127.22 (C-4a_Quin_), 135.94 (C-4_Quin_), 139.16 (C-8a_Quin_), 147.95 (C-4_Triaz_), 153.88 (C-2_Quin_), 157.19 (C-8_Quin_); HRMS (ESI-TOF): calcd for C_17_H_21_N_4_O_2_ ([M + H]^+^): *m*/*z* 313.1665; found: *m*/*z* 313.1664.

4-Hydroxymethyl-1-*N*-(2,3,4,6-tetra-*O*-acetyl-β-D-glucopyranosyl)-1*H*-1,2,3-triazol **M9**: Starting from 2,3,4,6-tetra-*O*-acetyl-β-D-glucopyranosyl azide **11** and propargyl alcohol **2**, the product was obtained as a white solid (91% yield); m.p.: 165–166 °C; [α]^24^_D_ = −15.8 (c = 1.0, CHCl_3_); ^1^H-NMR (400 MHz, CDCl_3_): δ 1.88, 2.03, 2.07, 2.08 (4s, 12H, CH_3_CO), 4.01 (ddd, 1H, *J* = 2.2 Hz, *J* = 5.0 Hz, *J* = 10.1 Hz, H-5_Glu_), 4.15 (dd, 1H, *J* = 2.2 Hz, *J* = 12.6 Hz, H-6a_Glu_), 4.30 (dd, 1H, *J* = 5.0 Hz, *J* = 12.6 Hz, H-6b_Glu_), 4.81 (bs, 2H, CH_2_OH), 5.25 (dd~t, 1H, *J* = 9.5 Hz, *J* = 10.1 Hz, H-4_Glu_), 5.39–4.49 (m, 2H, H-2_Glu_, H-3_Glu_), 5.89 (d, 1H, *J* = 9.3 Hz, H-1_Glu_), 7.79 (s, 1H, H-5_Triaz_); ^13^C-NMR (100 MHz, CDCl_3_): δ 20.19, 20.51, 20.54, 20.67 (CH_3_CO), 56.61 (CH_2_OH), 61.56 (C-6_Glu_), 67.73, 70.35, 72.67, 75.14 (C-2_Glu_, C-3_Glu_, C-4_Glu_, C-5_Glu_), 85.76 (C-1_Glu_), 120.04 (C-5_Triaz_), 148.42 (C-4_Triaz_), 169.00, 169.36, 169.90, 170.48 (CH_3_CO); HRMS (ESI-TOF): calcd for C_17_H_24_N_3_O_10_ ([M + H]^+^): *m*/*z* 430.1462; found: *m*/*z* 430.1457.

4-Hydroxymethyl-1-*N*-(β-D-glucopyranosyl)-1*H*-1,2,3-triazol **M10**: Starting from β-D-glucopyranosyl azide **12** and propargyl alcohol **2**, the product was obtained as a brown solid (90% yield); m.p.: 142–145 °C; [α]^24^_D_ = 3.0 (c = 1.0, CH_3_OH); ^1^H-NMR (400 MHz, DMSO): δ 3.23 (m, 1H, H-2_Glu_), 3.34–3.48 (m, 3H, H-3_Glu_, H-4_Glu_, H-5_Glu_), 3.64–3.80 (m, 2H, H-6a_Glu_, H-6b_Glu_), 4.53 (m, 2H, CH_2_OH), 4.60 (m, 1H, OH), 5.12 (d, 1H, *J* = 4.9 Hz, OH), 5.18 (t, 1H, *J* = 4.3 Hz, 6-OH), 5.24 (d, 1H, *J* = 3.9 Hz, OH), 5.33 (d, 1H, *J* = 5.8 Hz, OH), 5.50 (d, 1H, *J* = 9.3 Hz, H-1_Glu_), 8.11 (s, 1H, H-5_Triaz_); ^13^C-NMR (100 MHz, DMSO): δ 54.91 (CH_2_OH), 60.77 (C-6_Glu_), 69.58, 72.04, 77.01, 79.87 (C-2_Glu_, C-3_Glu_, C-4_Glu_, C-5_Glu_), 87.37 (C-1_Glu_), 121.87 (C-5_Triaz_), 147.74 (C-4_Triaz_); HRMS (ESI-TOF): calcd for C_9_H_16_N_3_O_6_ ([M + H]^+^): *m*/*z* 262.1039; found: *m*/*z* 262.1037.

4-Hydroxymethyl-1-(2-(2,3,4,6-tetra-*O*-acetyl-β-D-glucopyranosyloxy)ethyl)-1*H*-1,2,3-triazol **M11**: Starting from 2-azidoethyl 2,3,4,6-tetra-*O*-acetyl-β-D-glucopyranoside **13** and propargyl alcohol **2**, the product was obtained as a white solid (79% yield), m.p.: 151–152 °C; [α]^24^_D_ = −5.6 (c = 1.0, CHCl_3_); ^1^H-NMR (400 MHz, CDCl_3_): δ 1.96, 2.00, 2.02, 2.08 (4s, 12H, CH_3_CO), 3.71 (ddd, 1H, *J* = 2.4 Hz, *J* = 4.7 Hz, *J* = 9.9 Hz, H-5_Glu_), 3.92 (m, 1H, CH_2_O), 4.13 (dd, 1H, *J* = 2.4 Hz, *J* = 12.4 Hz, H-6a_Glu_), 4.23 (m, 1H, CH_2_O), 4.25 (dd, 1H, *J* = 4.7 Hz, *J* = 12.4 Hz, H-6b_Glu_), 4.48 (d, 1H, *J* = 7.8 Hz, H-1_Glu_), 4.50–4.67 (m, 2H, CH_2_N), 4.79 (s, 2H, CH_2_OH), 4.98 (dd, 1H, *J* = 7.8 Hz, *J* = 9.4 Hz H-2_glu_), 5.07 (dd~t, 1H, *J* = 9.4 Hz, *J* = 9.9 Hz, H-4_glu_), 5.17 (dd~t, 1H, *J* = 9.4 Hz, *J* = 9.4 Hz, H-3_glu_), 7.61 (s, 1H, H-5_Triaz_); ^13^C-NMR (100 MHz, CDCl_3_): δ 20.57, 20.57, 20.60, 20.72 (CH_3_CO), 49.97 (CH_2_N), 56.65 (CH_2_OH), 61.74 (C-6_Glu_), 67.86, 68.22, 71.12, 71.98, 72.46 (C-2_Glu_, C-3_Glu_, C-4_Glu_, C-5_Glu_, CH_2_O), 100.43 (C-1_Glu_), 123.36 (C-5_Triaz_), 147.67 (C-4_Triaz_), 169.47, 169.67, 170.09, 170.63 (CH_3_CO); HRMS (ESI-TOF): calcd for C_19_H_28_N_3_O_11_ ([M + H]^+^): *m/z* 474.1724; found: *m/z* 474.1725.

4-Hydroxymethyl-1-(2-(β-D-glucopyranosyloxy)ethyl)-1*H*-1,2,3-triazol **M12**: Starting from 2-azidoethyl β-D-glucopyranoside **14** and propargyl alcohol **2**, the product was obtained as a white solid (96% yield); m.p.: 60–63 °C; [α]^22^_D_ = −5.0 (c = 1.0, DMSO); ^1^H-NMR (400 MHz, DMSO): δ 2.96 (m, 1H, H-2_Glu_), 3.04 (m, 1H, H-4_Glu_), 3.09–3.16 (m, 2H, H-3_Glu_, H-5_Glu_), 3.43 (m, 1H, H-6a_Glu_), 3.67 (m, 1H, H-6b_Glu_), 3.90 (m, 1H, CH_2_O), 4.00–4.14 (m, 3H, CH_2_O, CH_2_N), 4.23 (d, 1H, *J* = 7.8 Hz, H-1_Glu_), 4.51 (d, 2H, *J* = 5.4 Hz, CH_2_OH), 4.55 (t, 1H, *J* = 5.4 Hz, OH), 4.92 (d, 1H, *J* = 5.2 Hz, OH), 4.95 (d, 1H, *J* = 4.8 Hz, OH), 5.06 (d, 1H, *J* = 4.9 Hz, OH), 5.14 (t, 1H, *J* = 5.6 Hz, 6-OH), 8.01 (s, 1H, H-5_triaz_); ^13^C-NMR (100 MHz, DMSO): δ 49.57 (CH_2_N), 55.02 (CH_2_OH), 61.07 (C-6_Glu_), 67.44, 70.02, 73.33, 76.62, 77.00 (C-2_Glu_, C-3_Glu_, C-4_Glu_, C-5_Glu_, CH_2_O), 102.96 (C-1_Glu_), 123.48 (C-5_Triaz_), 147.70 (C-4_Triaz_); HRMS (ESI-TOF): calcd for C_11_H_20_N_3_O_7_ ([M + H]^+^): *m*/*z* 306.1301; found: *m*/*z* 306.1300.

1-Hydroxyethyl-4-((2,3,4,6-tetra-*O*-acetyl-β-D-glucopyranosyloxy)methyl)-1*H*-1,2,3-triazol **M13**: Starting from propargyl 2,3,4,6-tetra-*O*-acetyl-β-D-glucopyranoside **15** and 2-azidoethanol **1**, the product was obtained as a colorless oil (76% yield); [α]^25^_D_ = −24.0 (c = 1.0, CHCl_3_); ^1^H-NMR (400 MHz, CDCl_3_): δ 2.00, 2.00, 2.03, 2.09 (4s, 12H, CH_3_CO), 3.73 (ddd, 1H, *J* = 2.4 Hz, *J* = 4.7 Hz, *J* = 10.0 Hz, H-5_Glu_), 4.03–4.10 (m, 2H, CH_2_OH), 4.16 (dd, 1H, *J* = 2.4 Hz, *J* = 12.3 Hz, H-6a_Glu_), 4.25 (dd, 1H, *J* = 4.7 Hz, *J* = 12.3 Hz, H-6b_Glu_), 4.42–4.56 (m, 2H, CH_2_N), 4.70 (d, 1H, *J* = 7.9 Hz, H-1_Glu_), 4.84 and 4.92 (qAB, 2H, *J* = 12.7 Hz, CH_2_C), 4.98 (dd, 1H, *J* = 7.9 Hz, *J* = 9.5 Hz H-2_Glu_), 5.09 (dd~t, 1H, *J* = 9.4 Hz, *J* = 10.0 Hz, H-4_Glu_), 5.21 (dd~t, 1H, *J* = 9.4 Hz, *J* = 9.5 Hz, H-3_Glu_), 7.67 (s, 1H, H-5_Triaz_); ^13^C-NMR (100 MHz, CDCl_3_): δ 20.60, 20.69, 20.78 (CH_3_CO), 52.72 (CH_2_N), 61.15, 61.82, 63.10 (CH_2_OH, CH_2_O, C-6_Glu_), 68.36, 71.42, 71.94, 72.73 (C-2_Glu_, C-3_Glu_, C-4_Glu_, C-5_Glu_), 99.98 (C-1_Glu_), 124.10 (C-5_Triaz_), 144.09 (C-4_Triaz_), 169.43, 169.55, 170.20, 170.79 (CH_3_CO); HRMS (ESI-TOF): calcd for C_19_H_28_N_3_O_11_ ([M + H]^+^): *m*/*z* 474.1724; found: *m*/*z* 474.1726.

2-(4-(Hydroxymethyl)-1*H*-1,2,3-triazol-1-yl)-*N*-(2,3,4,6-tetra-*O*-acetyl-β-D-glucopyranosyl)acetamide **M14**: Starting from 2,3,4,6-tetra-*O*-acetyl-*N*-(β-D-glucopyranosyl)azidoacetamide **16** and propargyl alcohol **2**, the product was obtained as a white solid (92% yield); m.p.: 173–174 °C; [α]^24^_D_ = 4.8 (c = 1.0, CHCl_3_); ^1^H-NMR (400 MHz, CDCl_3_): δ 2.00, 2.03, 2.03, 2.08 (4s, 12H, CH_3_CO), 3.82 (ddd, 1H, *J* = 2.2 Hz, *J* = 4.4 Hz, *J* = 10.0 Hz, H-5_Glu_), 4.10 (dd, 1H, *J* = 2.2 Hz, *J* = 12.5 Hz, H-6a_Glu_), 4.27 (dd, 1H, *J* = 4.4 Hz, *J* = 12.5 Hz, H-6b_Glu_), 4.82 (s, 2H, CH_2_OH), 4.87 (dd~t, 1H, *J* = 9.5 Hz, *J* = 9.5 Hz, H-2_Glu_), 5.03 and 5.14 (qAB, 2H, *J* = 16.6 Hz, CH_2_N), 5.04 (dd~t, 1H, *J* = 9.5 Hz, *J* = 10.0 Hz H-4_Glu_), 5.20 (dd~t, 1H, *J* = 9.1 Hz, *J* = 9.5 Hz, H-1_Glu_), 5.28 (dd~t, 1H, *J* = 9.5 Hz, *J* = 9.5 Hz, H-3_Glu_), 6.92 (d, 1H, *J* = 9.1 Hz, NH), 7.66 (s, 1H, H-5_Triaz_); ^13^C-NMR (100 MHz, CDCl_3_): δ 20.53, 20.56, 20.59, 20.72 (CH_3_CO), 52.74 (CH_2_N), 56.44 (CH_2_OH), 61.54 (C-6_Glu_), 68.01, 70.39, 72.54, 73.81 (C-2_Glu_, C-3_Glu_, C-4_Glu_, C-5_Glu_), 78.36 (C-1_Glu_), 123.38 (C-5_Triaz_), 148.62 (C-4_Triaz_), 165.90 (C=O), 169.49, 169.83, 170.62, 171.16 (CH_3_CO); HRMS (ESI-TOF): calcd for C_19_H_27_N_4_O_11_ ([M + H]^+^): *m*/*z* 487.1676; found: *m*/*z* 487.1676.

2-(4-(Hydroxymethyl)-1*H*-1,2,3-triazol-1-yl)-*N*-(β-D-glucopyranosyl)acetamide **M15**: Starting from *N*-(β-D-glucopyranosyl)azidoacetamide **17** and propargyl alcohol **2**, the product was obtained as a yellow solid (86% yield); m.p.: 155–158 °C; [α]^23^_D_ = 14.0 (c = 1.0, DMSO); ^1^H-NMR (400 MHz, DMSO): δ 3.03–3.15 (m, 3H, H-2_Glu_, H-4_Glu_, H-5_Glu_), 3.19 (m, 1H, H-3_Glu_), 3.42 (m, 1H, H-6a_Glu_), 3.64 (m, 1H, H-6b_Glu_), 4.52 (s, 2H, CH_2_OH), 4.71 (dd~t, 1H, *J* = 8.9 Hz, *J* = 9.0 Hz, H-1_Glu_), 5.08 and 5.13 (qAB, 2H, *J* = 16.4 Hz, CH_2_N), 7.91 (s, 1H, H-5_Triaz_), 8.94 (d, 1H, *J* = 9.0 Hz, NH); ^13^C-NMR (100 MHz, DMSO): δ 51.47 (CH_2_N), 55.02 (CH_2_OH), 60.82 (C-6_Glu_), 69.86, 72.61, 77.33, 78.73 (C-2_Glu_, C-3_Glu_, C-4_Glu_, C-5_Glu_), 79.67 (C-1_Glu_), 124.31 (C-5_Triaz_), 147.70 (C-4_Triaz_), 165.95 (C=O), HRMS (ESI-TOF): calcd for C_11_H_19_N_4_O_7_ ([M + H]^+^): *m*/*z* 319.1254; found: *m*/*z* 319.1251.

1-(2-Hydroxyethyl)-*N*-(2,3,4,6-tetra-*O*-acetyl-β-D-glucopyranosyl)-1*H*-1,2,3-triazole-4-carboxamide **M16**: Starting from 2,3,4,6-tetra-*O*-acetyl-*N*-(β-D-glucopyranosyl)propiolamide **18** and 2-azidoethanol **1**, the product was obtained as a white solid (88% yield); m.p.: 194–196 °C; [α]^23^_D_ = −15.6 (c = 1.0, CHCl_3_); ^1^H-NMR (600 MHz, CDCl_3_): δ 1.99, 2.03, 2.05, 2.07 (4s, 12H, CH_3_CO), 3.91 (ddd, 1H, *J* = 2.2 Hz, *J* = 4.3 Hz, *J* = 10.0 Hz, H-5_Glu_), 4.06–4.11 (m, 2H, CH_2_OH), 4.10 (dd, 1H, *J* = 2.2 Hz, *J* = 12.5 Hz, H-6a_Glu_), 4.27 (dd, 1H, *J* = 4.3 Hz, *J* = 12.5 Hz, H-6b_Glu_), 4.51–4.59 (m, 2H, CH_2_N), 5.13 (dd~t, 1H, *J* = 9.6 Hz, *J* = 10.0 Hz, H-4_Glu_), 5.15 (dd~t, 1H, *J* = 9.5 Hz, *J* = 9.5 Hz H-2_glu_), 5.35 (dd~t, 1H, *J* = 9.5 Hz, *J* = 9.5 Hz, H-1_glu_), 5.47 (dd~t, 1H, *J* = 9.5 Hz, *J* = 9.6 Hz, H-3_Glu_), 7.93 (d, 1H, *J* = 9.6 Hz, NH), 8.27 (s, 1H, H-5_Triaz_); ^13^C-NMR (150 MHz, CDCl_3_): δ 20.58, 20.61, 20.73 (CH_3_CO), 52.97 (CH_2_N), 60.86 (CH_2_OH), 61.68 (C-6_Glu_), 68.16, 70.43, 73.07, 73.63 (C-2_Glu_, C-3_Glu_, C-4_Glu_, C-5_Glu_), 77.84 (C-1_Glu_), 127.38 (C-5_Triaz_), 141.85 (C-4_Triaz_), 160.53 (C=O), 169.55, 170.06, 170.30, 170.71 (CH_3_CO); HRMS (ESI-TOF): calcd for C_19_H_27_N_4_O_11_ ([M + H]^+^): *m*/*z* 487.1676; found: *m*/*z* 487.1678.

(1-(2-Hydroxyethyl)-1*H*-1,2,3-triazol-4-yl)methyl (2,3,4,6-tetra-O-acetyl-β-D-glucopyranosyl)carbamate **M17**: Starting from 2,3,4,6-tetra-*O*-acetyl-*N*-(β-D-glucopyranosyl)-*O*-propargyl carbamate **19** and 2-azidoethanol **1**, the product was obtained as a white solid (83% yield); m.p.: 93–95 °C; [α]^23^_D_ = 0.8 (c = 1.0, CHCl_3_); ^1^H-NMR (400 MHz, CDCl_3_): δ 2.01, 2.03, 2.07 (4s, 12H, CH_3_CO), 3.83 (m, 1H, H-5_Glu_), 4.00–4.08 (m, 2H, CH_2_OH), 4.13 (m, 1H, H-6a_Glu_), 4.29 (m, 1H, H-6b_Glu_), 4.40–4.58 (m, 2H, CH_2_N), 4.94 (m, 1H, H-1_Glu_), 4.99–5.19 (m, 3H, CH_2_O, H-2_Glu_), 5.20–5.35 (m, 2H, H-3_Glu_, H-4_Glu_), 6.06 (m, 1H, NH), 7.75 (s, 1H, H-5_Triaz_); ^13^C NMR (100 MHz, CDCl_3_): δ 20.57, 20.58, 20.66, 20.73 (CH_3_CO), 52.74 (CH_2_N), 58.64, 61.00, 61.58 (CH_2_OH, CH_2_O, C-6_Glu_), 68.07, 70.18, 72.93, 73.32 (C-2_Glu_, C-3_Glu_, C-4_Glu_, C-5_Glu_), 80.78 (C-1_Glu_), 125.01 (C-5_Triaz_), 142.36 (C-4_Triaz_), 155.49 (C=O), 169.53, 169.95, 170.56, 170.64 (CH_3_CO); HRMS (ESI-TOF): calcd for C_20_H_29_N_4_O_12_ ([M + H]^+^): *m*/*z* 517.1782; found: *m*/*z* 517.1780.

4-Hydroxymethyl-1-((2,3,4,6-tetra-*O*-acetyl-β-D-glucopyranosyl)thiomethyl)-1*H*-1,2,3-triazol **M18**: Starting from azidomethyl 2,3,4,6-tetra-*O*-acetyl-1-thio-β-D-glucopyranoside **20** and propargyl alcohol **2**, the product was obtained as a white solid (78% yield); m.p.: 135–137 °C; [α]^25^_D_ = −33.0 (c = 1.0, CHCl_3_); ^1^H NMR (400 MHz, CDCl_3_): δ 2.00, 2.03, 2.05, 2.10 (4s, 12H, CH_3_CO), 3.69 (ddd, 1H, *J* = 2.5 Hz, *J* = 4.2 Hz, *J* = 10.0 Hz, H-5_Glu_), 4.12 (dd, 1H, *J* = 4.2 Hz, *J* = 12.5 Hz, H-6a_Glu_), 4.17 (dd, 1H, *J* = 2.5 Hz, *J* = 12.5 Hz, H-6b_Glu_), 4.64 (d, 1H, *J* = 10.1 Hz, H-1_Glu_), 4.81 (bs, 2H, CH_2_OH), 5.03 (dd~t, 1H, *J* = 9.3 Hz, *J* = 10.1 Hz, H-2_Glu_), 5.09 (dd~t, 1H, *J* = 9.4 Hz, *J* = 10.0 Hz, H-4_Glu_), 5.20 (dd~t, 1H, *J* = 9.3 Hz, *J* = 9.4 Hz, H-3_Glu_), 5.39 i 5.67 (qAB, 2H, *J* = 14.7 Hz, CH_2_N), 7.77 (s, 1H, H-5_Triaz_); ^13^C-NMR (100 MHz, CDCl_3_): δ 20.56, 20.58, 20.61, 20.82 (CH_3_CO), 48.61 (CH_2_N), 56.78 (CH_2_OH), 61.52 (C-6_Glu_), 67.92, 69.58, 73.57, 76.21 (C-2_Glu_, C-3_Glu_, C-4_Glu_, C-5_Glu_), 82.37 (C-1_Glu_), 121.69 (C-5_Triaz_), 148.96 (C-4_Triaz_), 169.33, 169.45, 170.02, 170.83 (CH_3_CO); HRMS (ESI-TOF): calcd for C_18_H_26_N_3_O_10_S ([M + H]^+^): *m*/*z* 476.1339; found: *m*/*z* 476.1339.

4-Hydroxymethyl-1-((2,3,4,6-tetra-*O*-acetyl-β-D-glucopyranosyl)thioethyl)-1*H*-1,2,3-triazol **M19**: Starting from 2-azidoethyl 2,3,4,6-tetra-*O*-acetyl-1-thio-β-D-glucopyranoside **21** and propargyl alcohol **2**, the product was obtained as a white solid (86% yield); m.p.: 150 °C; [α]^24^_D_ = −31.8 (c = 1.0, CHCl_3_); ^1^H-NMR (400 MHz, CDCl_3_): δ 2.01, 2.04, 2.06, 2.08 (4s, 12H, CH_3_CO), 3.06 (m, 1H, CHS), 3.27 (m, 1H, CHS), 3.73 (ddd, 1H, *J* = 3.1 Hz, *J* = 4.3 Hz, *J* = 10.1 Hz, H-5_Glu_), 4.19 (dd, 1H, *J* = 3.1 Hz, *J* = 12.5 Hz, H-6a_Glu_), 4.23 (dd, 1H, *J* = 4.3 Hz, *J* = 12.5 Hz, H-6b_Glu_), 4.44 (d, 1H, *J* = 10.0 Hz, H-1_Glu_), 4.53–4.69 (m, 2H, CH_2_N), 4.81 (s, 2H, CH_2_OH), 5.04 (dd~t, 1H, *J* = 9.4 Hz, *J* = 10.0 Hz, H-2_Glu_), 5.08 (dd~t, 1H, *J* = 9.4 Hz, *J* = 10.1 Hz, H-4_Glu_), 5.22 (dd~t, 1H, *J* = 9.4 Hz, *J* = 9.4 Hz, H-3_Glu_), 7.68 (s, 1H, H-5_Triaz_); ^13^C-NMR (100 MHz, CDCl_3_): δ 20.57, 20.58, 20.66, 20.75 (CH_3_CO), 30.34 (CH_2_S), 50.50 (CH_2_N), 56.61 (CH_2_OH), 61.92 (C-6_Glu_), 68.12, 69.48, 73.54, 76.23 (C-2_Glu_, C-3_Glu_, C-4_Glu_, C-5_Glu_), 83.63 (C-1_Glu_), 122.77 (C-5_Triaz_), 147.63 (C-4_Triaz_), 169.40, 169.46, 170.08, 170.62 (CH_3_CO); HRMS (ESI-TOF): calcd for C_19_H_28_N_3_O_10_S ([M + H]^+^): *m*/*z* 490.1495; found: *m*/*z* 490.1497.

1-Hydroxyethyl-4-((2,3,4,6-tetra-*O*-acetyl-β-D-glucopyranosyl)thiomethyl)-1*H*-1,2,3-triazol **M20**: Starting from propargyl 2,3,4,6-tetra-*O*-acetyl-1-thio-β-D-glycopyranosides **22** and 2-azidoethanol **1**, the product was obtained as a colorless oil (73% yield); [α]^25^_D_ = −24.4 (c = 0.5, CHCl_3_); ^1^H-NMR (600 MHz, CDCl_3_): δ 2.00, 2.02, 2.03, 2.09 (4s, 12H, CH_3_CO), 3.73 (ddd, 1H, *J* = 2.4 Hz, *J* = 4.5 Hz, *J* = 10.1 Hz, H-5_Glu_), 3.93 and 4.03 (qAB, 2H, *J* = 14.8 Hz, CH_2_S), 4.03–4.09 (m, 2H, CH_2_OH), 4.15 (dd, 1H, *J* = 2.4 Hz, *J* = 12.4 Hz, H-6a_Glu_), 4.21 (dd, 1H, *J* = 4.5 Hz, *J* = 12.4 Hz, H-6b_Glu_), 4.43–4.52 (m, 2H, CH_2_N), 4.62 (d, 1H, *J* = 10.1 Hz, H-1_Glu_), 4.99 (dd, 1H, *J* = 9.3 Hz, *J* = 10.1 Hz H-4_Glu_), 5.10 (dd~t, 1H, *J* = 9.4 Hz, *J* = 10.1 Hz, H-2_Glu_), 5.20 (dd~t, 1H, *J* = 9.3 Hz, *J* = 9.4 Hz, H-3_Glu_), 7.65 (s, 1H, H-5_Triaz_); ^13^C-NMR (150 MHz, CDCl_3_): δ 20.58, 20.69, 20.79 (CH_3_CO), 24.23 (CH_2_S), 52.80 (CH_2_N), 61.12, 61.96 (CH_2_OH, C-6_Glu_), 68.29, 70.08, 73.84, 75.88 (C-2_Glu_, C-3_Glu_, C-4_Glu_, C-5_Glu_), 82.72 (C-1_Glu_), 123.49 (C-5_Triaz_), 144.79 (C-4_Triaz_), 169.43, 169.64, 170.14, 170.82 (CH_3_CO); HRMS (ESI-TOF): calcd for C_19_H_28_N_3_O_10_S ([M + H]^+^): *m*/*z* 490.1495; found: *m*/*z* 490.1493.

2-(4-(Hydroxymethyl)-1*H*-1,2,3-triazol-1-yl)-*N*-(6-((2,3,4,6-tetra-O-acetyl-β-D-glucopyranosyl)thio)pyridin-3-yl)acetamide **M21**: Starting from (5-azidoacetamide-2-pyridyl) 2,3,4,6-tetra-*O*-acetyl-1-thio-β-D-glucopyranoside **23** and propargyl alcohol **2**, the product was obtained as a white solid (83% yield); m.p.: 171–174 °C; [α]^25^_D_ = −7.8 (c = 0.9, CHCl_3_); ^1^H-NMR (400 MHz, DMSO): δ 1.96, 1.98, 1.98, 2.00 (4s, 12H, CH_3_CO), 4.02 (m, 1H, H-5_Glu_), 4.10–4.20 (m, 2H, H-6a_Glu_, H-6b_Glu_), 4.55 (d, 2H, *J* = 5.6 Hz, CH_2_OH), 4.93–5.04 (m, 2H, H-2_Glu_, H-4_Glu_), 5.20 (t, 1H, *J* = 5.6 Hz, OH), 5.34 (s, 2H, CH_2_N), 5.42 (dd~t, 1H, *J* = 9.4 Hz, *J* = 9.4 Hz, H-3_Glu_), 5.73 (d, 1H, *J* = 10.3 Hz, H-1_Glu_), 7.42 (d, 1H, *J* = 8.7 Hz, H-3_Pyr_), 7.97 (dd, 1H, *J* = 2.6 Hz, *J* = 8.7 Hz, H-4_Pyr_), 7.99 (s, 1H, H-5_Triaz_), 8.67 (d, 1H, *J* = 2.1 Hz, H-6_Pyr_), 10.72 (s, 1H, NH); ^13^C-NMR (100 MHz, DMSO): δ 20.27, 20.32, 20.37, 20.45 (CH_3_CO), 51.95 (CH_2_N), 55.01 (CH_2_OH), 61.83 (C-6_Glu_), 68.02, 69.39, 72.96, 74.51 (C-2_Glu_, C-3_Glu_, C-4_Glu_, C-5_Glu_), 81.15 (C-1_Glu_), 122.92 (C-5_Triaz_), 124.36 (C_Pyr_), 127.82 (C_Pyr_), 133.21 (C_Pyr_), 140.55 (C_Pyr_), 147.86 (C-4_Triaz_), 149.66 (C_Pyr_), 164.94 (C=O), 169.08, 169.27, 169.47, 169.88 (CH_3_CO); HRMS (ESI-TOF): calcd for C_24_H_30_N_5_O_11_S ([M + H]^+^): *m*/*z* 596.1663; found: *m*/*z* 596.1662.

(1-(2-Hydroxyethyl)-1*H*-1,2,3-triazol-4-yl)methyl (6-((2,3,4,6-tetra-*O*-acetyl-β-D-glucopyranosyl)thio)pyridin-3-yl)carbamate **M22**: Starting from (5-(((prop-2-yn-1-yloxy)carbonyl)amino)-2-pyridyl) 2,3,4,6-tetra-*O*-acetyl-1-thio-β-D-glucopyranoside **24** and 2-azidoethanol **1**, the product was obtained as a white solid (86% yield); m.p.: 124–126 °C; [α]^24^_D_ = −4.2 (c = 1.0, CHCl_3_); ^1^H-NMR (400 MHz, DMSO): δ 1.95, 1.97, 1.98, 2.00 (4s, 12H, CH_3_CO), 3.73–3.82 (m, 2H, CH_2_OH), 4.02 (m, 1H, H-5_Glu_), 4.09–4.19 (m, 2H, H-6a_Glu_, H-6b_Glu_), 4.41 (d, 2H, *J* = 5.4 Hz, CH_2_N), 4.93–5.06 (m, 2H, H-2_Glu_, H-4_Glu_), 5.22 (s, 2H, CH_2_O), 5.41 (dd~t, 1H, *J* = 9.4 Hz, *J* = 9.4 Hz, H-3_Glu_), 5.69 (d, 1H, *J* = 10.3 Hz, H-1_Glu_), 7.38 (d, 1H, *J* = 8.7 Hz, H-3_Pyr_), 7.84 (dd, 1H, *J* = 2.1 Hz, *J* = 8.7 Hz, H-4_Pyr_), 8.15 (s, 1H, H-5_Triaz_), 8.54 (d, 1H, *J* = 2.1 Hz, H-6_Pyr_), 10.02 (s, 1H, NH); ^13^C-NMR (100 MHz, DMSO): δ 20.18, 20.23, 20.28, 20.34 (CH_3_CO), 52.08 (CH_2_N), 57.67 (CH_2_O), 59.71 (CH_2_OH), 61.73 (C-6_Glu_), 67.93, 69.33, 72.90, 74.40 (C-2_Glu_, C-3_Glu_, C-4_Glu_, C-5_Glu_), 81.26 (C-1_Glu_), 123.05 (C-5_Triaz_), 125.17 (C_Pyr_), 126.59 (C_Pyr_), 133.84 (C_Pyr_), 139.68 (C_Pyr_), 141.52 (C-4_Triaz_), 148.02 (C_Pyr_), 153.12 (C=O), 168.98, 169.17, 169.38, 169.80 (CH_3_CO); HRMS (ESI-TOF): calcd for C_25_H_32_N_5_O_12_S ([M + H]^+^): *m*/*z* 626.1768; found: *m*/*z* 626.1769.

#### 3.2.2. Preparation and Characterization of Micelles

Micelles were prepared by standard solvent evaporation method from pH-responsive amphiphilic poly(ethylene glycol)-*b*-polycarbonate-*b*-oligo([R]-3-hydroxybutyrate) copolymer which was synthesized by PEG/organocatalyst-initiated ring-opening polymerization of ketal-protected six-membered cyclic carbonate and subsequent esterification with natural oligo([R]-3-hydroxybutyrate) as described in our previous study [42]. The size and morphology of micelles were determined by dynamic light scattering (DLS) measurements using a Brookhaven BI-200 goniometer equipped with a Brookhaven BI-9000 AT (Brookhaven Instruments Corp., Holtsville, NY, USA) and cryo-TEM (Tecnai F20 X TWIN mi-croscope (FEI Company, Hillsboro, OR, USA), respectively. The DLS and cryo-TEM measurement of the drug free micelles showed a relatively small hydrodynamic size of ~25.5 nm with a spherical shape [42] (Figure S45a,b). Meanwhile, metabolite-loaded mi-celles revealed particles size of around ~34 nm and ~36 nm for **M5**-micelles and **M7**-micelles, respectively (Figure S45c,d). Furthermore, the drug loading efficiency (DLE) and the drug loading content (DLC) were determined according to standard formulas. The encapsulation properties of pH-sensitive micelles were evaluated at the feed drug ratio of the copolymer was 1:10. The DLC and DLE values for micelles loaded with **M5** were 5.4% ± 0.1 and 49.9% ± 1.6 and with **M7** 5.2% ± 0.2 and 46.8% ± 2.9, respectively. No significant differences in encapsulation properties were observed between metabolites, which is probably related to the small difference in chemical structure. Detailed information on copolymer synthesis and physicochemical characterization of micelles has been provided in our former studies [42].

### 3.3. Biological Evaluation

#### 3.3.1. Cell Cultures

The human colon adenocarcinoma cell line (HCT-116) was purchased from the American Type Culture Collection (ATCC, Manassas, VA, USA). The human breast adenocarcinoma cell line (MCF-7) was obtained from collections at the Maria Sklodowska-Curie Memorial Cancer Center and National Institute of Oncology (Gliwice, Poland). The Normal Human Dermal Fibroblasts-Neonatal (NHDF-Neo) was purchased from LONZA (Cat. No. CC-2509, NHDF-Neo, Dermal Fibroblasts, Neonatal, Lonza, Poland). Cells were grown in a culture medium in a humidified atmosphere at 5% CO_2_ and 37 °C. The culture media consisted of RPMI 1640 or DMEM+F12 (HyClone), supplemented with 10% of heat-inactivated fetal bovine serum (FBS, EURx, Poland) and 1% of Antibiotic Antimycotic Solution, 100 U/mL penicillin, and 10 mg/mL streptomycin (Sigma-Aldrich, Taufkirchen, Germany). 

#### 3.3.2. MTT Assay

Cells viability was assessed by the MTT (3-[4,5–dimethylthiazol-2-yl]-2,5-diphenyltetrazolium bromide) test (Sigma-Aldrich, Taufkirchen, Germany) according to the manufacturer’s protocol. Stock solutions of tested compounds were prepared in DMSO and diluted to the desired concentrations with the appropriate volumes of the growth medium directly before the experiment (DMSO content in the highest concentration did not exceed 0.5%). The cells were seeded into 96-well plates at concentration 1 × 10^4^ (HCT-116, NHDF-Neo) or 5 × 10^3^ (MCF-7) per well and incubated for 24 h at 37 °C in a humidified atmosphere of 5% CO_2_. Then, the culture medium was removed, replaced with the solution of the tested compounds in a fresh medium with varying concentrations. As a control in the cytotoxicity studies, cells suspended in a medium supplemented with 0.5% DMSO were used. It was the amount of DMSO necessary to dissolve the highest concentration of a given sample. Then, cells were incubated for a further 24 h or 72 h. After this time, the medium was removed, and the MTT solution (50 µL, 0.5 mg/mL in PBS) was added into each well. After 3 h of incubation, the MTT solution was carefully removed, and the acquired formazan crystals were dissolved in DMSO. The absorbance was measured spectrophotometrically at the 570 nm wavelength using a multi-well plate reader (Epoch, BioTek, Winooski, VT, USA). The experiment was conducted in at least three independent repetitions with four technical repetitions for each tested concentration, and the results were expressed as the survival fraction (%) of the control. The IC_50_ values were calculated using CalcuSyn software (version 2.0, Biosoft, Cambridge, UK). The IC_50_ parameter was defined as the concentration of drug that was necessary to reduce the proliferation of cells to 50% of the untreated control. The results are shown as the average value ± SD.

## 4. Conclusions

As part of the research, the ability to inhibit the proliferation of cancer cells by glycoconjugates derivatives of 8-hydroxyquinoline and their metabolites, which theoretically may be released in biological systems under the action of hydrolytic enzymes, were compared. For the study, compounds were selected consisting of an 8-hydroxyquinoline fragment linked by an aliphatic chain of various lengths to a 1,2,3-triazole ring as well as D-glucose derivatives containing anomeric oxygen, nitrogen, or sulfur atom also connected to the 1,2,3-triazole ring by various linkers. The applied CuAAC reaction allowed for a quick and efficient synthesis of metabolites, which in the next stage were subjected to MTT cytotoxicity screening tests.

The performed studies prove that the presence of all three building blocks is necessary for the full cytotoxic activity of glycoconjugates: the sugar unit, the linker containing a 1,2,3-triazole ring, and the 8-hydroxyquinoline fragment. The sugar fragment improves the pharmacokinetic properties of potential drugs. On the other hand, a heteroaromatic fragment increases the cytotoxicity of glycoconjugates, probably by improving the chelating capacity of metal ions, thus inhibiting the growth of neoplastic cells. The toxic effect of metabolites on normal cells indicates that further studies are necessary to improve the transport of prodrugs directly to the tumor cells. Therefore, it is worth continuing the idea of adding a sugar unit to 8-HQ, however, by using a position other than the anomeric position that participates in binding to GLUT transporters, so that the obtained derivatives will have sufficient affinity for these transporters.

The high IC_50_ values of the metabolites do not clearly indicate their lack of anticancer activity. Perhaps these compounds cannot reach the target site of action. The encapsulation of selected metabolites in micelles significantly improved their cytotoxic effect through the possibility of using a much lower therapeutic dose of the drug. The use of pH-sensitive micelles allows the drug to be released close to the tumor cell, preventing early release of the drug into the systemic circulation, thus reducing its side effects.

## Figures and Tables

**Table 1:** Biological activity of the obtained compounds (metabolites of 8-hydroxyquinoline derivatives and glycoconjugates from which they could arise).

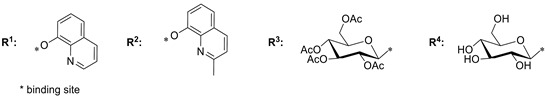								
Compound number	Structure	Activity IC_50_ [µM] ^a^			Glycoconjugate Structure ^d^	Activity IC_50_ [µM]		
		HCT-116^b^	MCF-7^c^	NHDF^b^		HCT-116	MCF-7	NHDF
**M1**	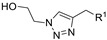	>800	>800	-	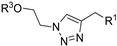	239.95 ± 2.27	105.91 ± 4.06	216.12 ± 9.68
**M2**	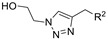	>800	>800	-	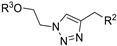	290.62 ± 7.02	135.97 ± 1.47	715.16 ± 10.63
**M3**	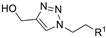	>800	>800	-	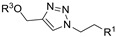	216.95 ± 4.73	196.49 ± 1.91	405.85 ± 5.73
**M4**	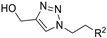	>800	750.45 ± 1.07	-	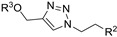	229.56 ± 2.59	375.58 ± 8.34	-
**M5**	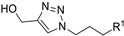	>800	602.95 ± 1.95	-	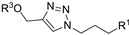	142.98 ± 2.30	200.60 ± 1.10	214.75 ± 6.43
**M6**	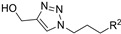	469.82 ± 8.61	194.13 ± 0.18	202.02 ± 3.29	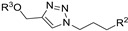	135.07 ± 6.98	221.11 ± 2.40	426.80 ± 3.80
**M7**	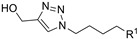	196.18 ± 3.55	155.96 ± 0.45	131.99 ± 1.00	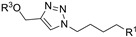	328.75 ± 9.02	254.81 ± 3.63	-
**M8**	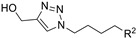	564.85 ± 3.59	286.01± 1.42	-	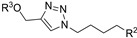	294.74 ± 1.79	214.83 ± 1.65	>800

^a^ Cytotoxicity was evaluated using the MTT assay; ^b^ Incubation time 24 h; ^c^ Incubation time 72 h; ^d^ The synthesis and biological activity of the presented glycoconjugates are described in publications [26,27,28]. Data are presented as the mean ± standard deviation (*n* = 3).

**Table 2:** Biological activity of the obtained compounds (metabolites of sugar derivatives and glycoconjugates from which they could arise).

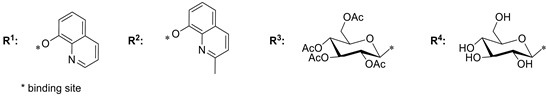								
Compound Number	Structure	Activity IC_50_ [µM] ^a^			Glycoconjugate Structure ^d^	Activity IC_50_ [µM]		
		HCT-116 ^b^	MCF-7 ^c^	NHDF ^b^		HCT-116	MCF-7	NHDF
**M9**	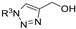	>800	>800	-	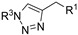	69.00 ± 2.53	57.69 ± 3.32	57.37 ± 3.19
**M10**	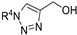	>800	>800	-	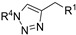	212.00 ± 7.71	185.34 ± 2.21	247.24 ± 11.64
**M11**	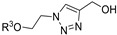	>800	>800	-	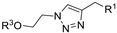	239.95 ± 2.27	105.91 ± 4.06	216.12 ± 9.68
**M12**	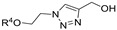	>800	>800	-	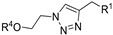	>800	>800	-
**M13**	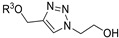	>800	>800	-	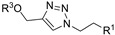	216.95 ± 4.73	196.49 ± 1.91	405.85 ± 5.73
**M14**	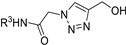	258.32 ± 2.06	428.66 ± 2.11	101.15 ± 4.98	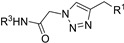	246.24 ± 6.19	192.66 ± 3.71	219.14 ± 2.40
**M15**	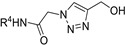	747.66 ± 8.29	>800	-	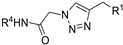	112.79 ± 1.58	87.89 ± 4.11	94.69 ± 0.46
**M16**	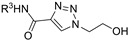	>800	>800	-	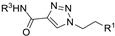	239.05 ± 2.97	203.78 ± 3.55	382.61 ± 2.42
**M17**	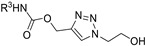	>800	>800	-	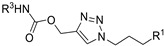	246.23 ± 1.31	176.40 ± 1.81	696.74 ± 1.60
**M18**	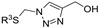	107.24 ± 2.17	248.77 ± 1.58	89.07 ± 8.63	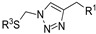	106.71 ± 4.10	59.12 ± 1.46	54.62 ± 0.74
**M19**	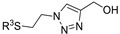	>800	792.99 ± 1.30	-	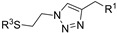	127.05 ± 1.75	76.30 ± 1.33	105.32 ± 3.40
**M20**	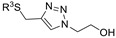	>800	>800	-	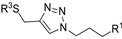	172.83 ± 3.48	153.34 ± 0.25	229.12 ± 2.06
**M21**	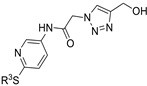	>800	>800	-	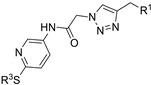	146.16 ± 3.49	69.72 ± 3.50	71.81 ± 6.70
**M22**	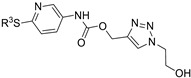	>800	>800	-	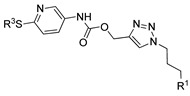	63.49 ± 2.37	67.50 ± 1.58	64.00 ± 5.34

^a^ Cytotoxicity was evaluated using the MTT assay; ^b^ Incubation time 24 h; ^c^ Incubation time 72 h; ^d^ The synthesis and biological activity of the presented glycoconjugates are described in publications [26,27,28]. Data are presented as the mean ± standard deviation (*n* = 3).

**Table 3:** Biological activity of the free metabolites and metabolites loaded into micelles.

Compound Number	Structure	Activity IC_50_ [µM] ^a^		
		HCT-116 ^b^	MCF-7 ^b^	NHDF ^b^
**M5**	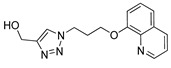	>800	602.95 ± 1.95	509.52 ± 3.26
**M5-micelles**		23.59 ± 1.44	33.04 ± 1.73	59.54 ± 2.81
**M7**	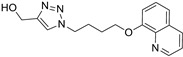	169.19 ± 3.90	155.96 ± 0.45	85.14 ± 4.16
**M7-micelles**		12.41 ± 0.41	4.46 ± 0.36	45.76 ± 1.78

^a^ Cytotoxicity was evaluated using the MTT assay; ^b^ Incubation time 72 h. Data are presented as the mean ± standard deviation (*n* = 3).

## Data Availability

Data are contained within the article.

## Supplementary Materials

The following supporting information can be downloaded online. Figures S1–S44: ^1^H and ^13^C-NMR spectra of all obtained compounds; Figure S45: Characterization of micelles.

## Sample Availability

Samples of the compounds **M1**–**M22** are available from the authors.
