# Supplementary material for: Synthesis and Preliminary Evaluation of the Cytotoxicity of Potential Metabolites of Quinoline Glycoconjugates
Source: Molecules. 2022 Feb 3;27(3):1040. doi: 10.3390/molecules27031040 (PMC8838273; doi:10.3390/molecules27031040)
Supplement: Supplementary file 1 [file molecules-27-01040-s001.zip › molecules-1557129-supplementary.pdf]

Supplementary Materials

Synthesis and Preliminary Evaluation of the Cytotoxicity of Potential Metabolites of Quinoline Glycoconjugates

Monika Domińska [1,2,*], Gabriela Pastuch-Gawołek [1,2,*], Adrian Domiński [3], Piotr Kurcok [3] and Karol Erfurt [4]

[1] Department of Organic Chemistry, Bioorganic Chemistry and Biotechnology, Silesian University of Technology, B. Krzywoustego 4, 44-100 Gliwice, Poland
[2] Biotechnology Centre, Silesian University of Technology, B. Krzywoustego 8, 44-100 Gliwice, Poland
[3] Centre of Polymer and Carbon Materials, Polish Academy of Sciences, M. Curie-Skłodowskiej 34, 41-819 Zabrze, Poland; adrian.dominski@cmpw-pan.edu.pl (A.D.); piotr.kurcok@cmpw-pan.edu.pl (P.K.)
[4] Department of Chemical Organic Technology and Petrochemistry, Silesian University of Technology, B. Krzywoustego 4, 44-100 Gliwice, Poland; karol.erfurt@polsl.pl
* Correspondence: monika.krawczyk@polsl.pl (M.D.); gabriela.pastuch@polsl.pl (G.P.-G.)

^{1}H and ^{13}C NMR spectra of all obtained compounds

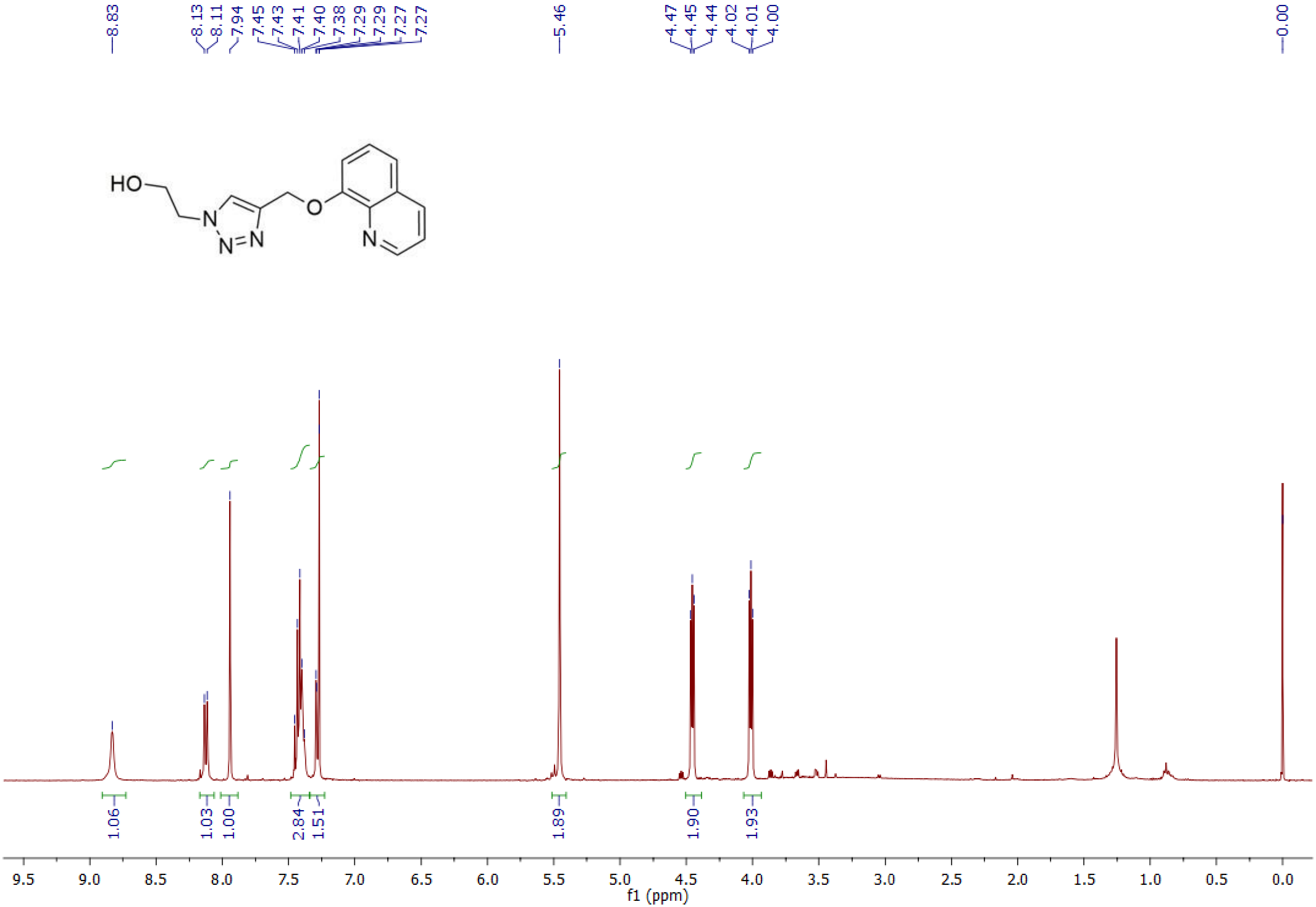

Figure S1. ^{1}H NMR spectrum of compound **M1** (400 MHz/$CDCl_3$/TMS; δ (ppm)).

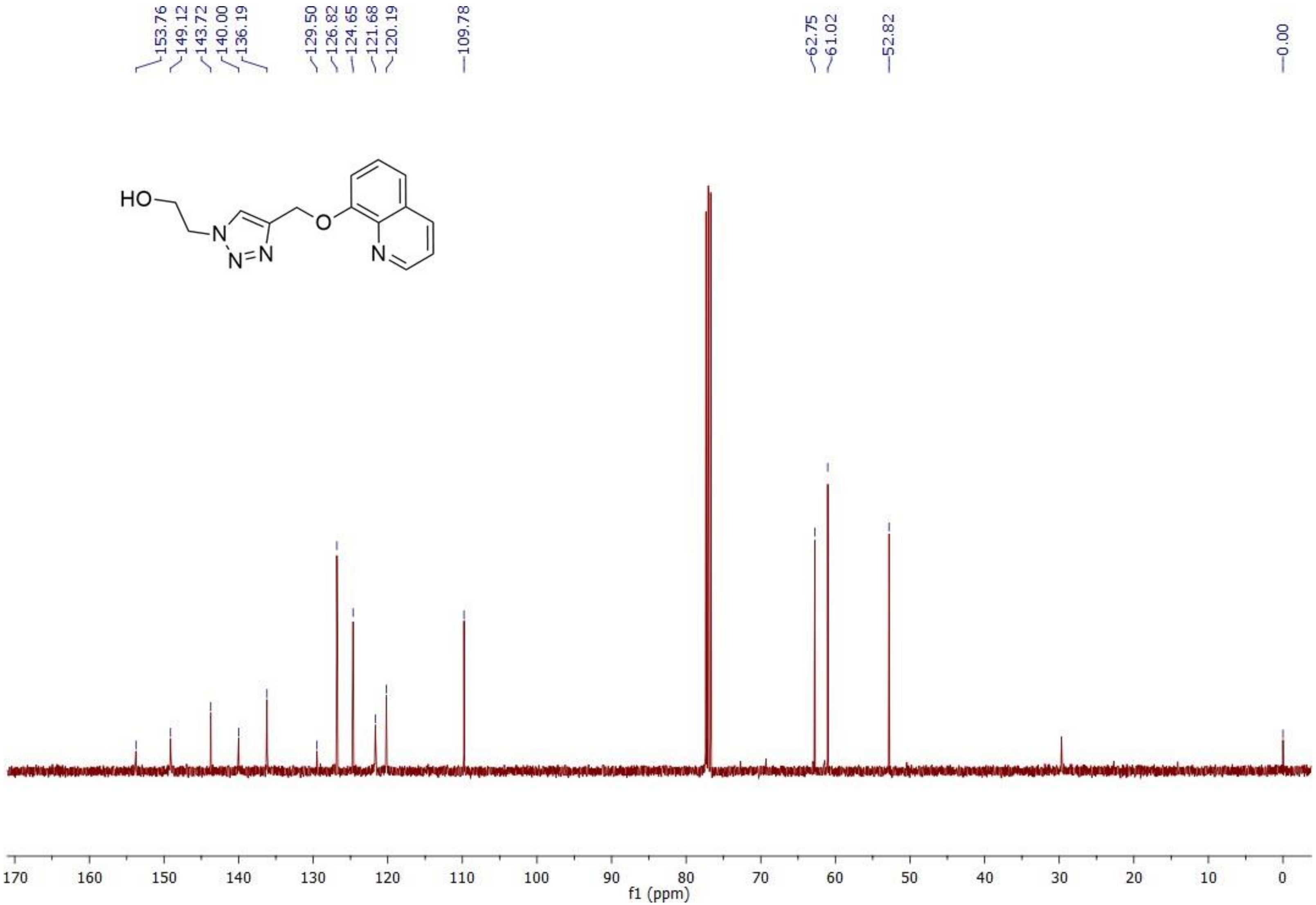

Figure S2. ^{13}C NMR spectrum of compound **M1** (100 MHz/$CDCl_3$/TMS; δ (ppm)).

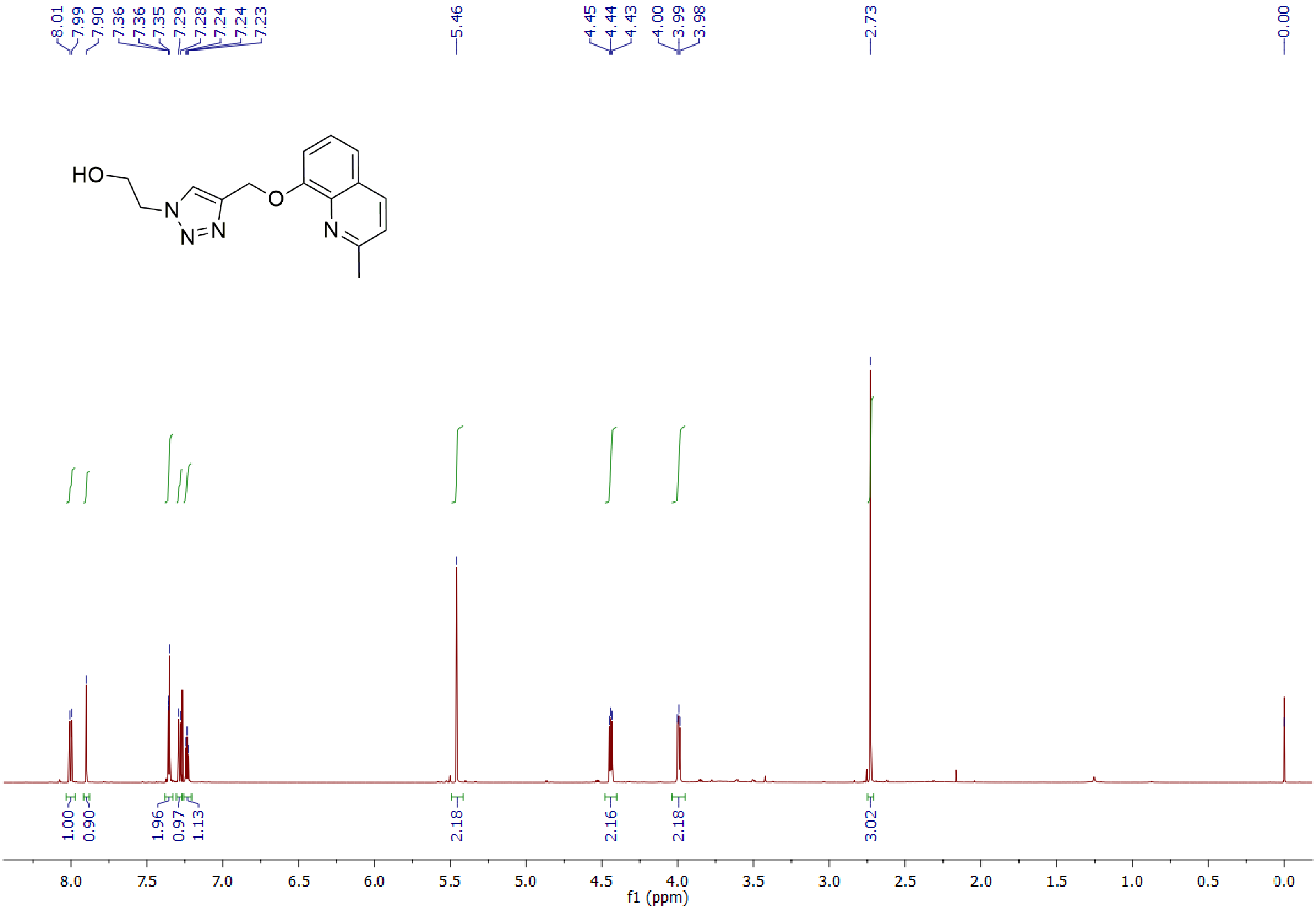

Figure S3. ^{1}H NMR spectrum of compound **M2** (600 MHz/$CDCl_3$/TMS; δ (ppm)).

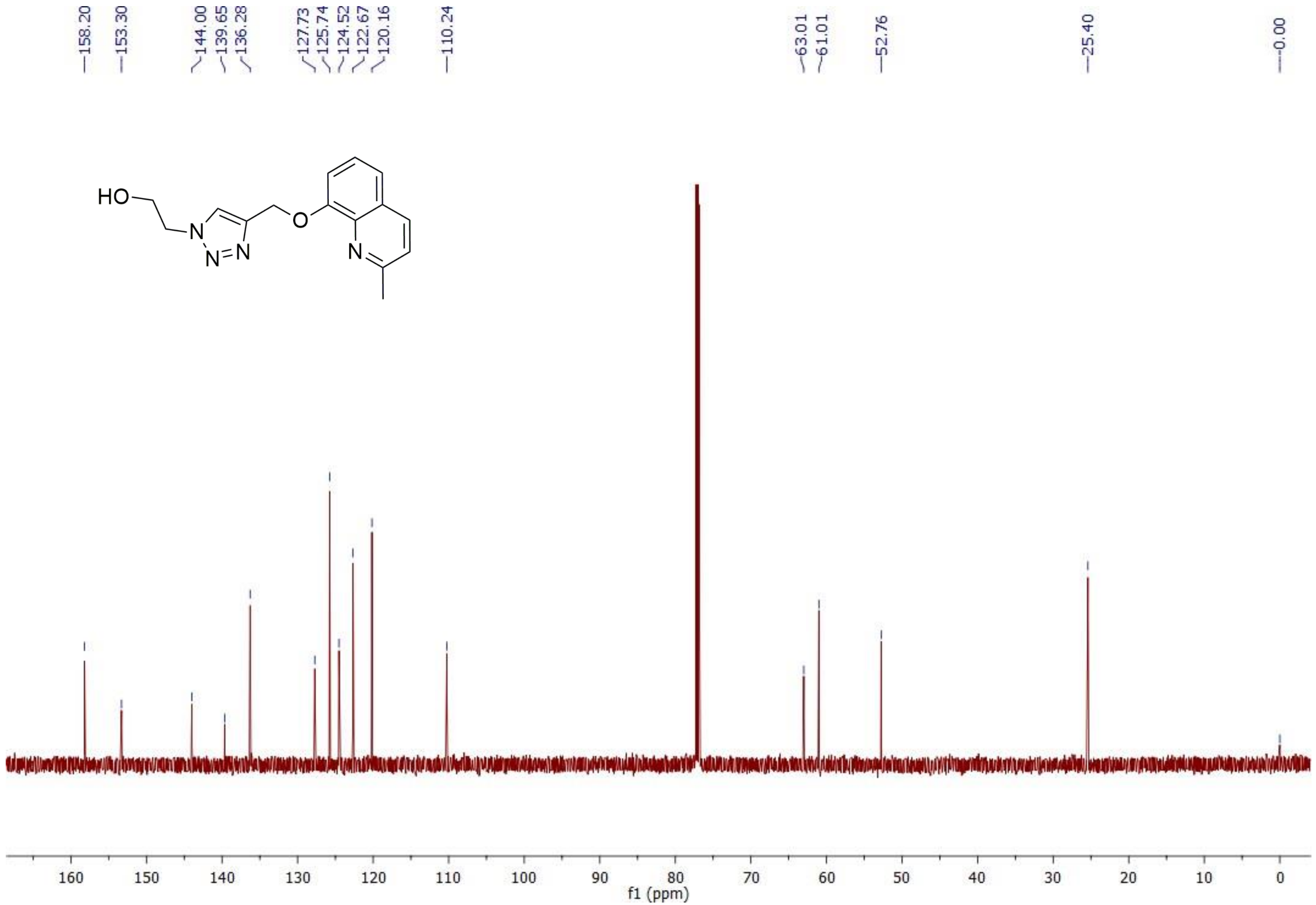

Figure S4. ^{13}C NMR spectrum of compound **M2** (150 MHz/$CDCl_3$/TMS; δ (ppm)).

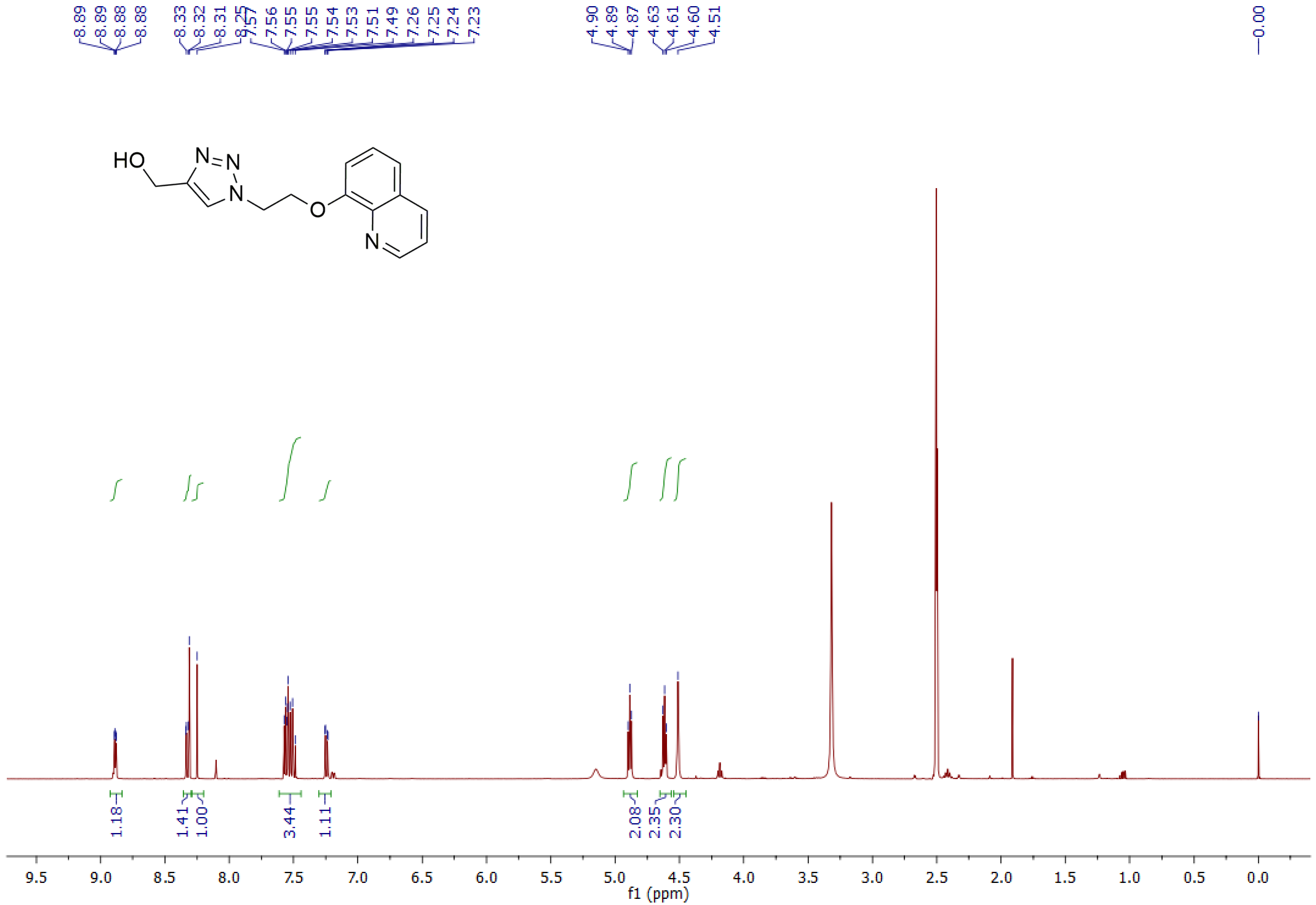

Figure S5. ^{1}H NMR spectrum of compound **M3** (400 MHz/DMSO/TMS; δ (ppm)).

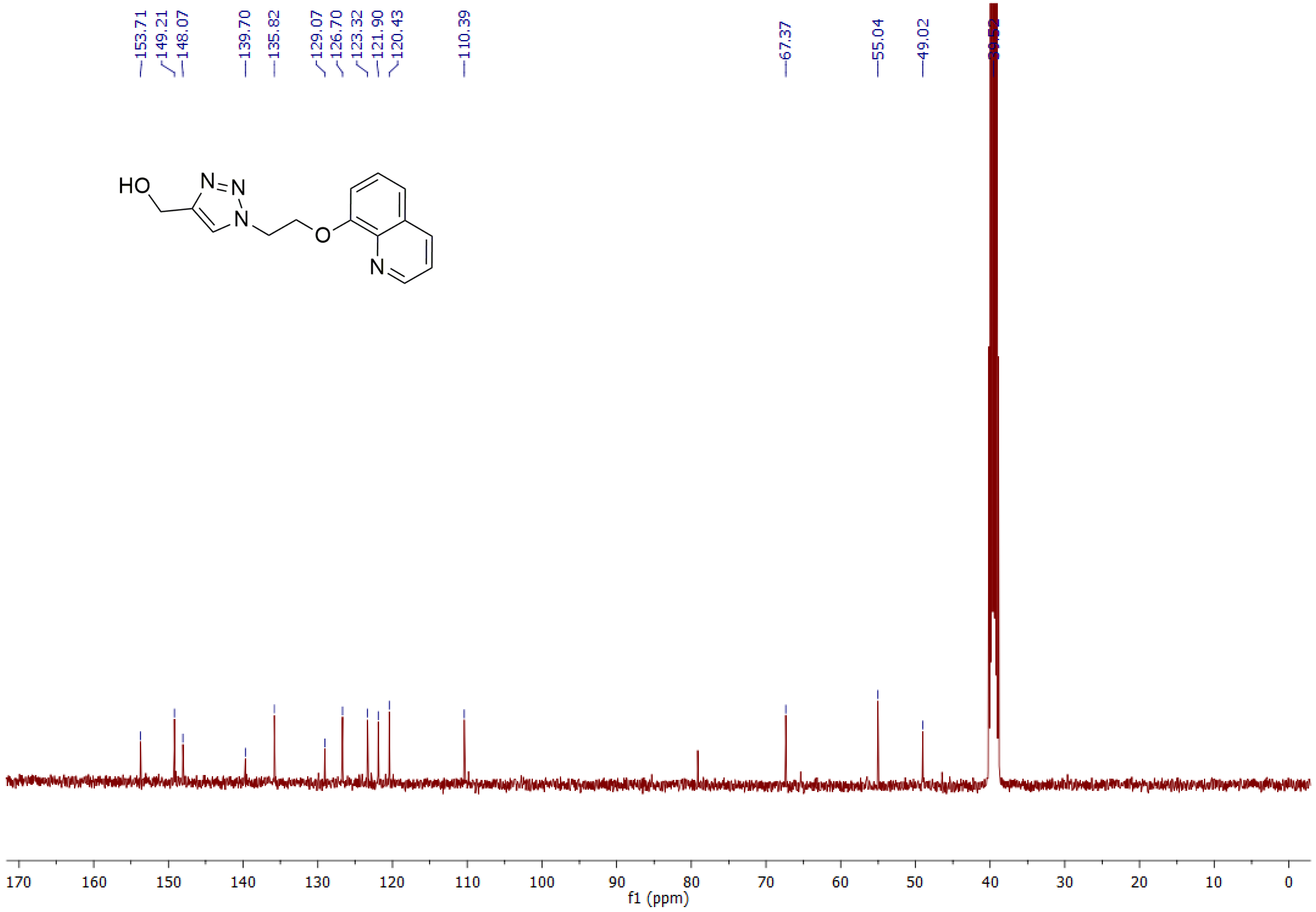

Figure S6. ^{13}C NMR spectrum of compound **M3** (100 MHz/DMSO/TMS; δ (ppm)).

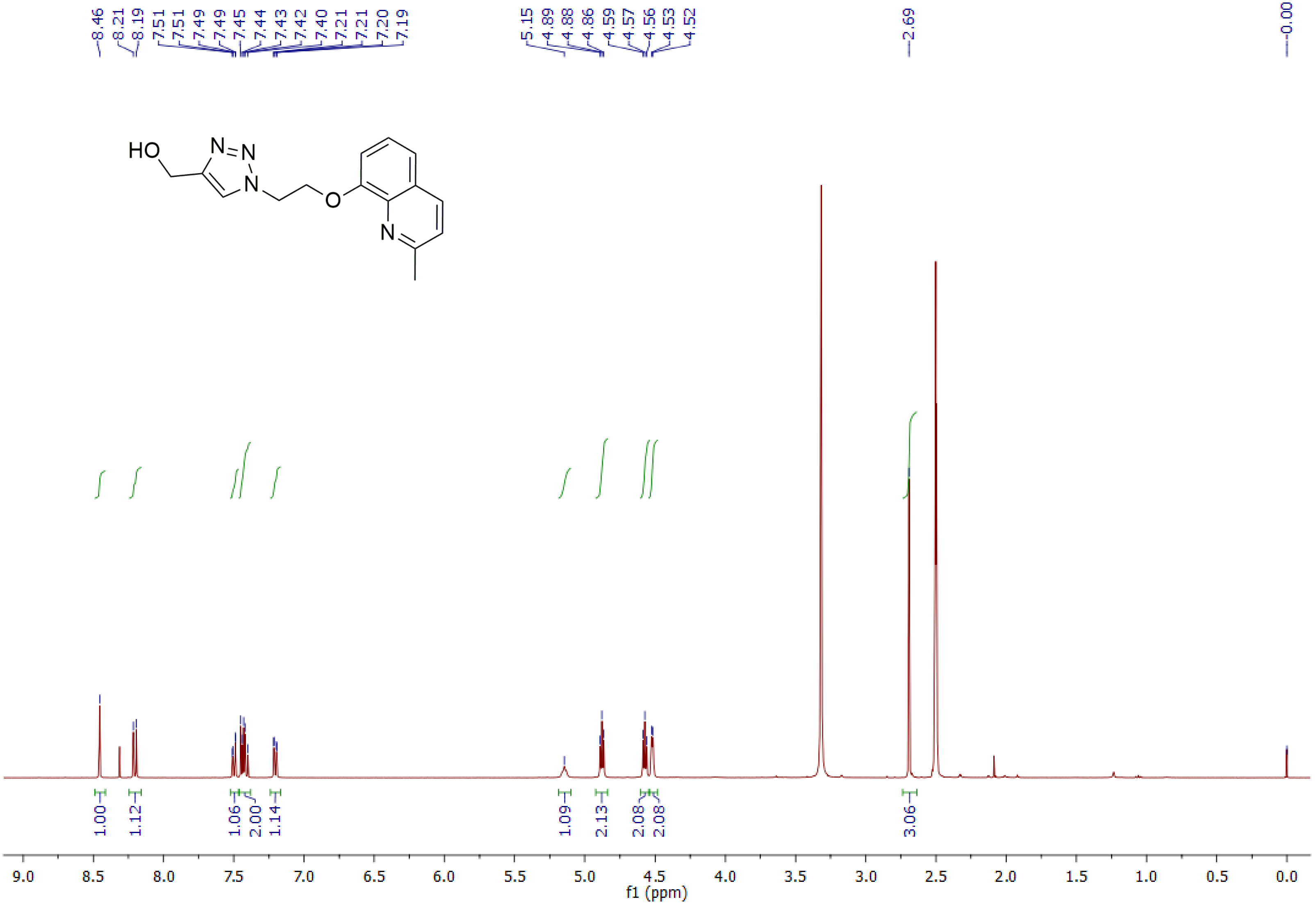

Figure S7. ^{1}H NMR spectrum of compound **M7** (400 MHz/DMSO/TMS; δ (ppm)).

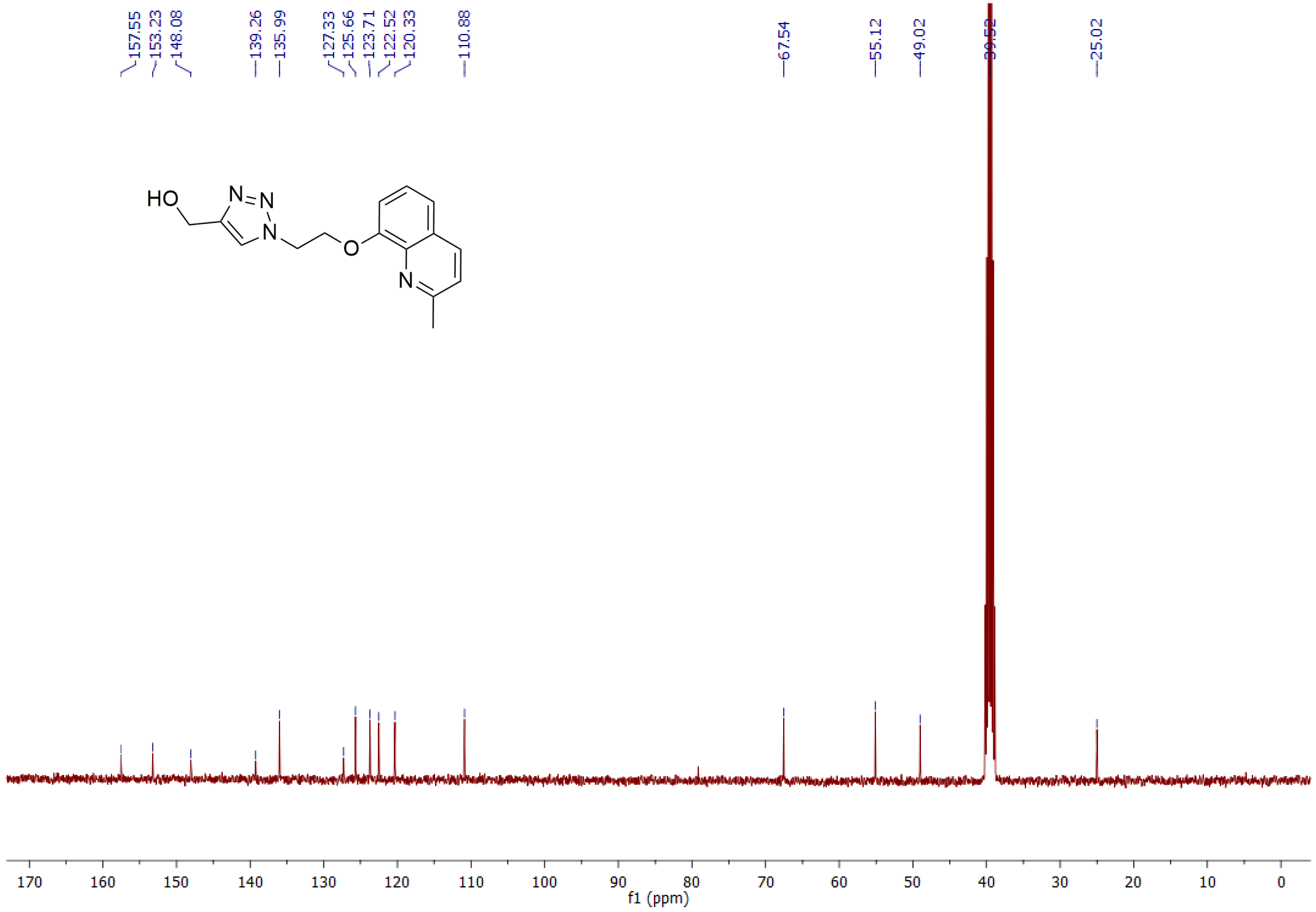

Figure S8. ^{13}C NMR spectrum of compound **M5** (100 MHz/DMSO/TMS; δ (ppm)).

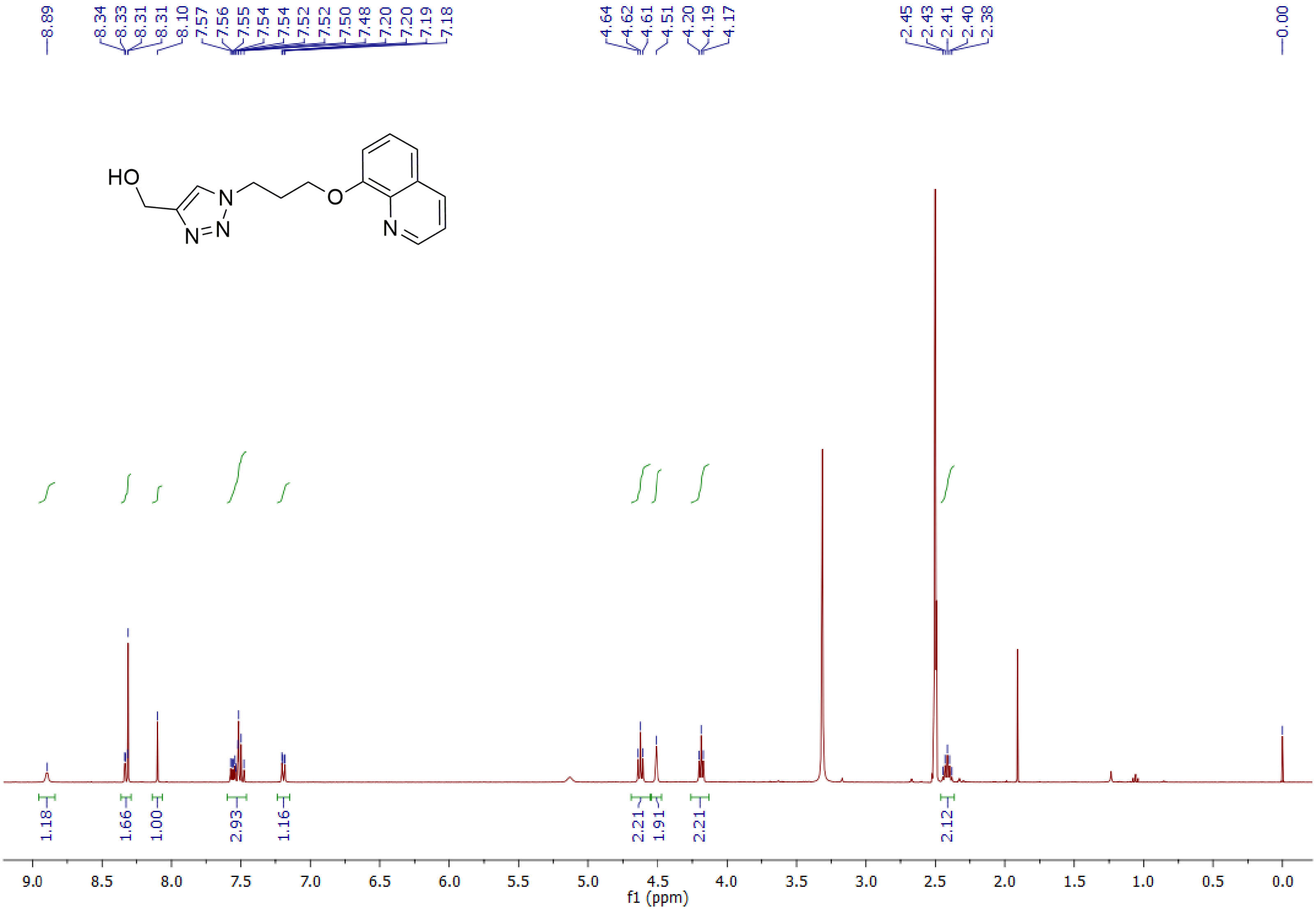

Figure S9. ^{1}H NMR spectrum of compound **M9** (400 MHz/DMSO/TMS; δ (ppm)).

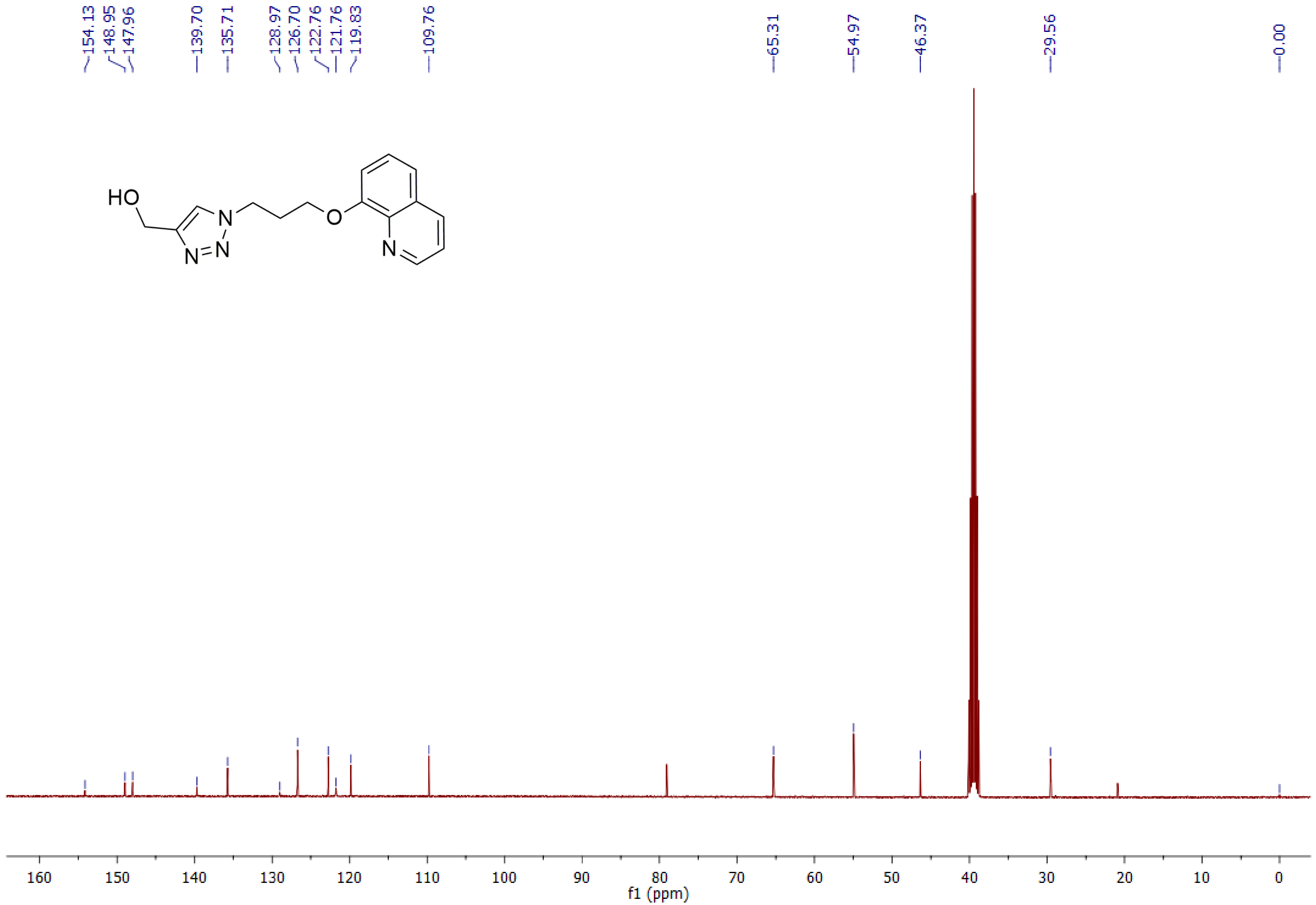

Figure S10. ^{13}C NMR spectrum of compound **M5** (100 MHz/DMSO/TMS; δ (ppm)).

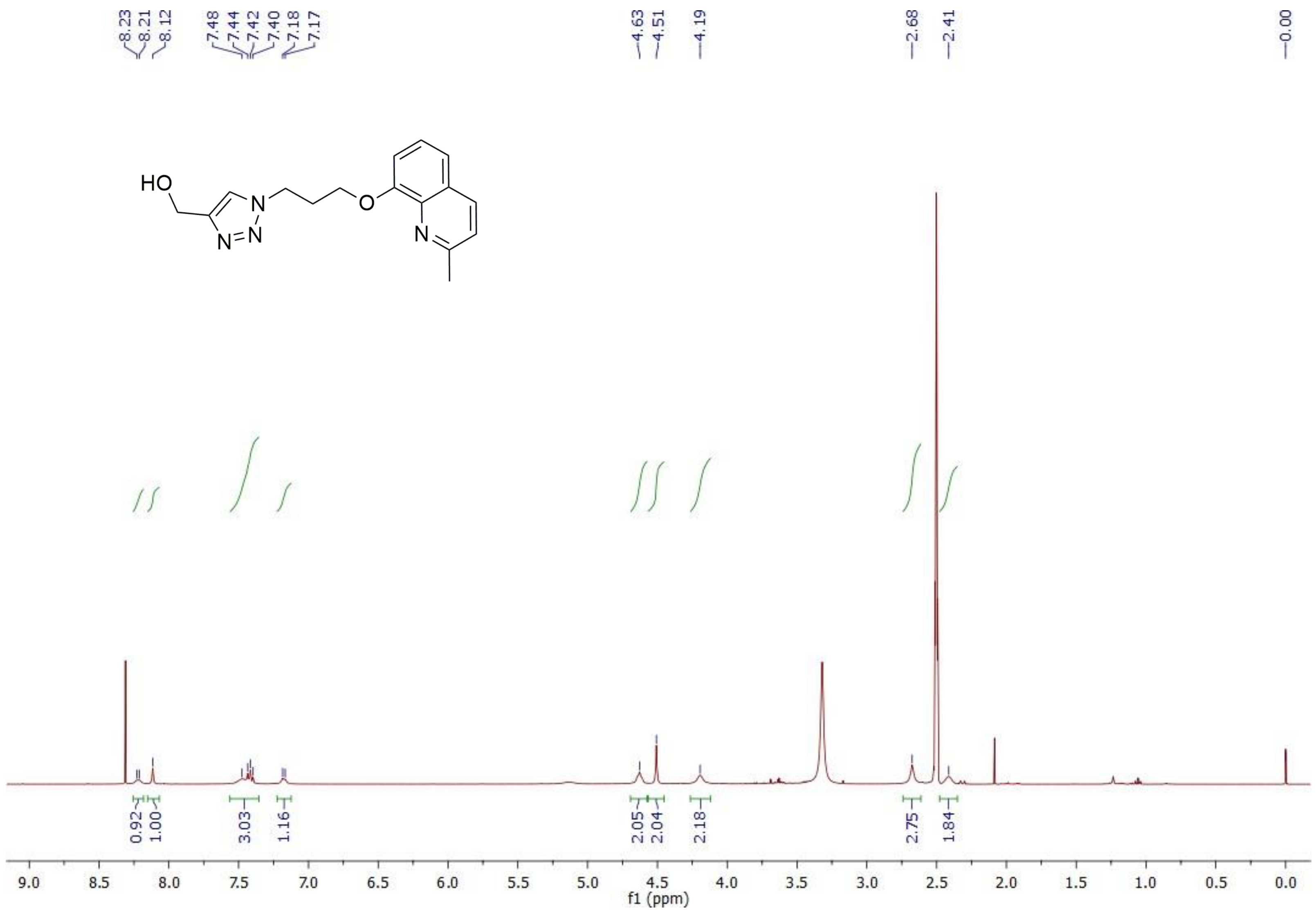

Figure S11. ^{1}H NMR spectrum of compound **M11** (400 MHz/DMSO/TMS; δ (ppm)).

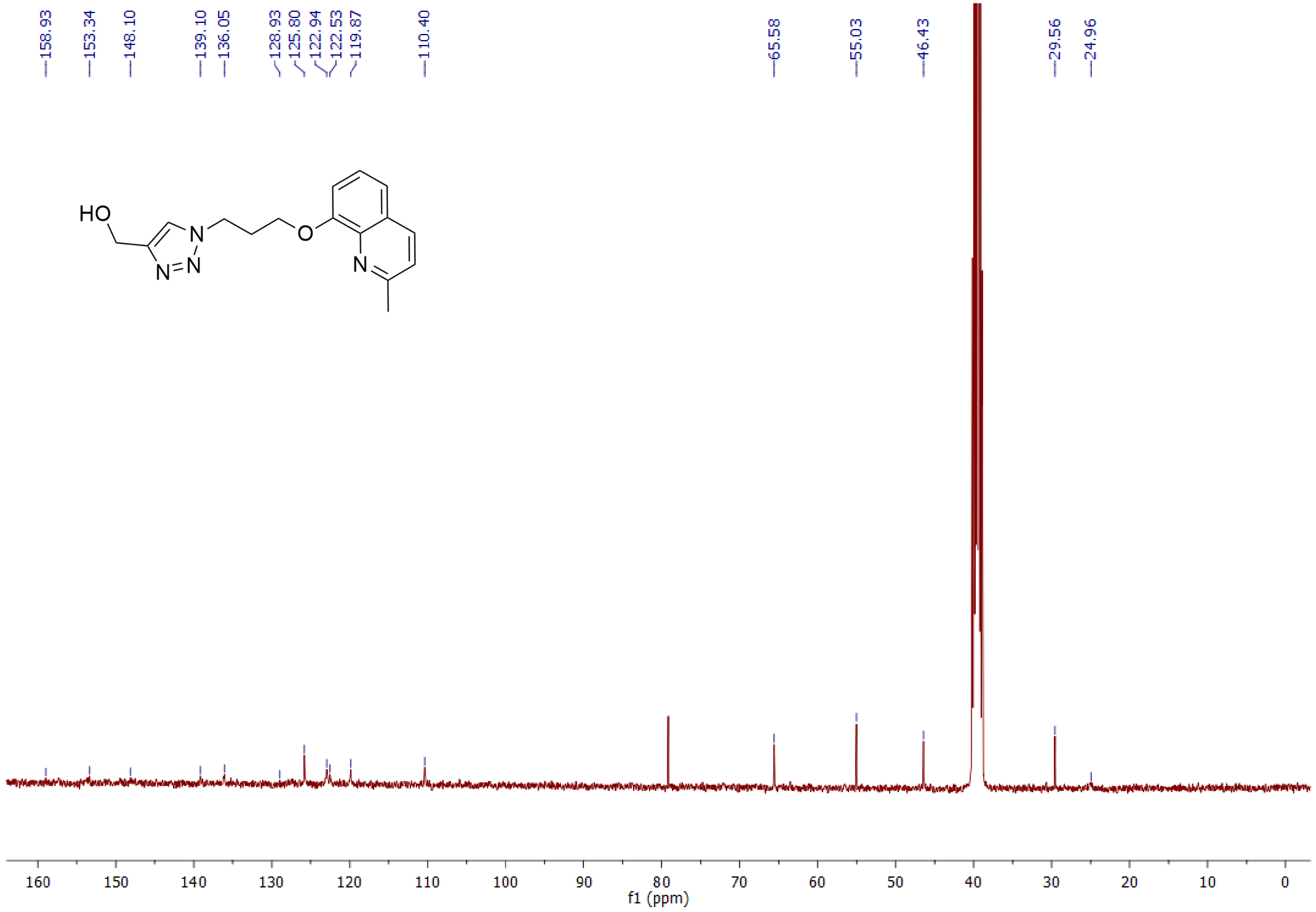

Figure S12. ^{13}C NMR spectrum of compound **M5** (100 MHz/DMSO/TMS; δ (ppm)).

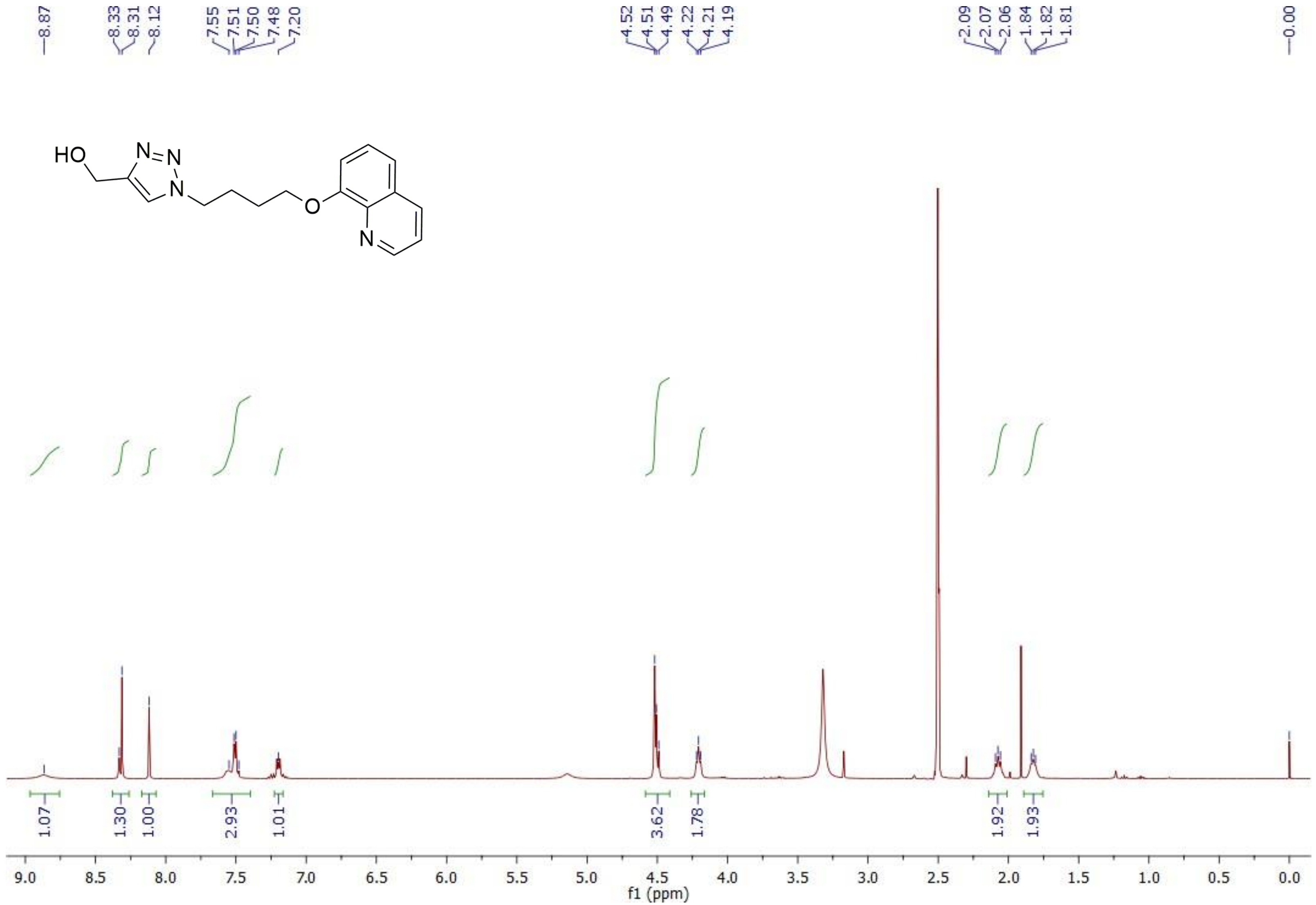

Figure S13. ^{1}H NMR spectrum of compound **M13** (400 MHz/DMSO/TMS; δ (ppm)).

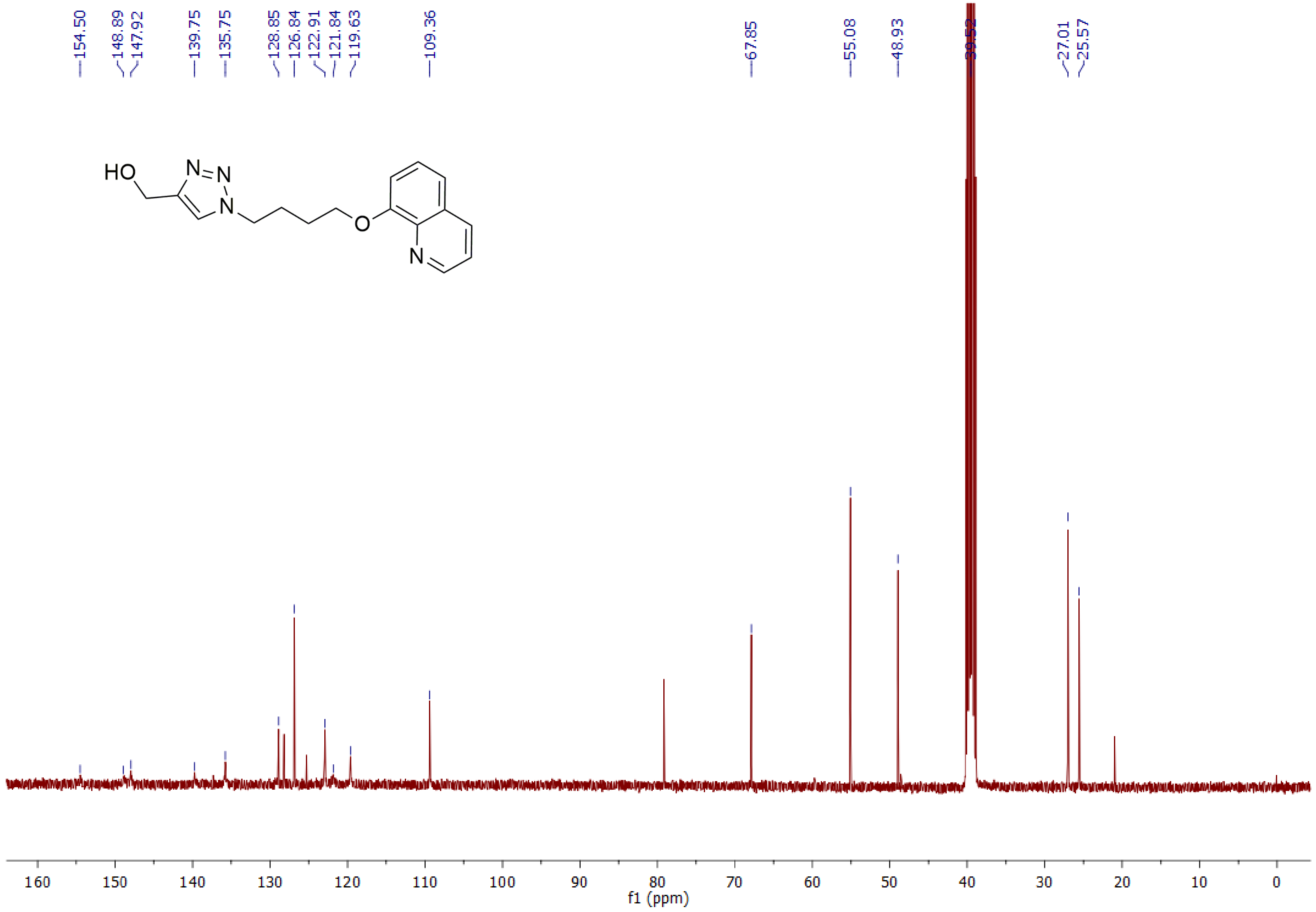

Figure S14. ^{13}C NMR spectrum of compound **M5** (100 MHz/DMSO/TMS; δ (ppm)).

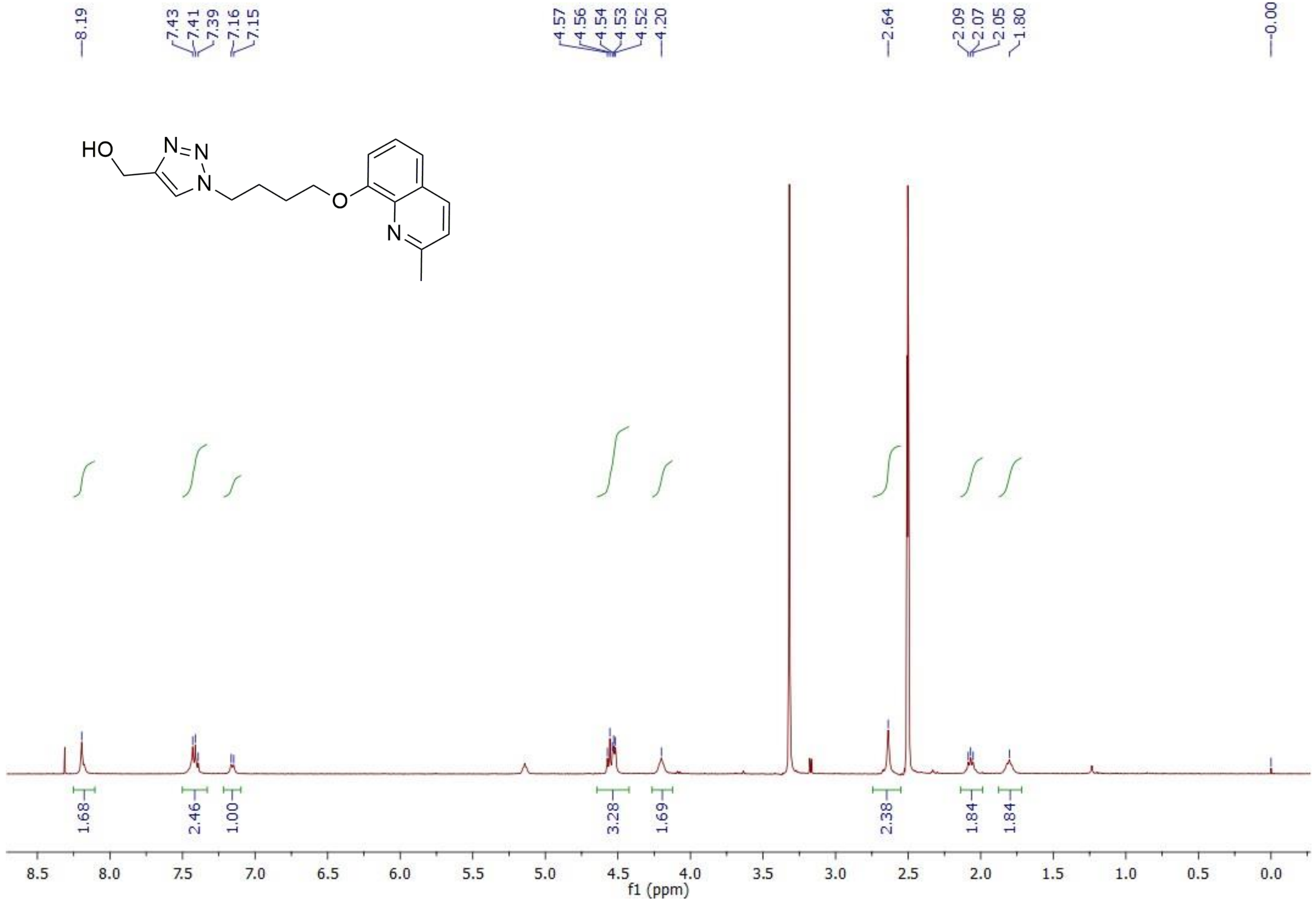

Figure S15. ^{1}H NMR spectrum of compound **M15** (400 MHz/DMSO/TMS; δ (ppm)).

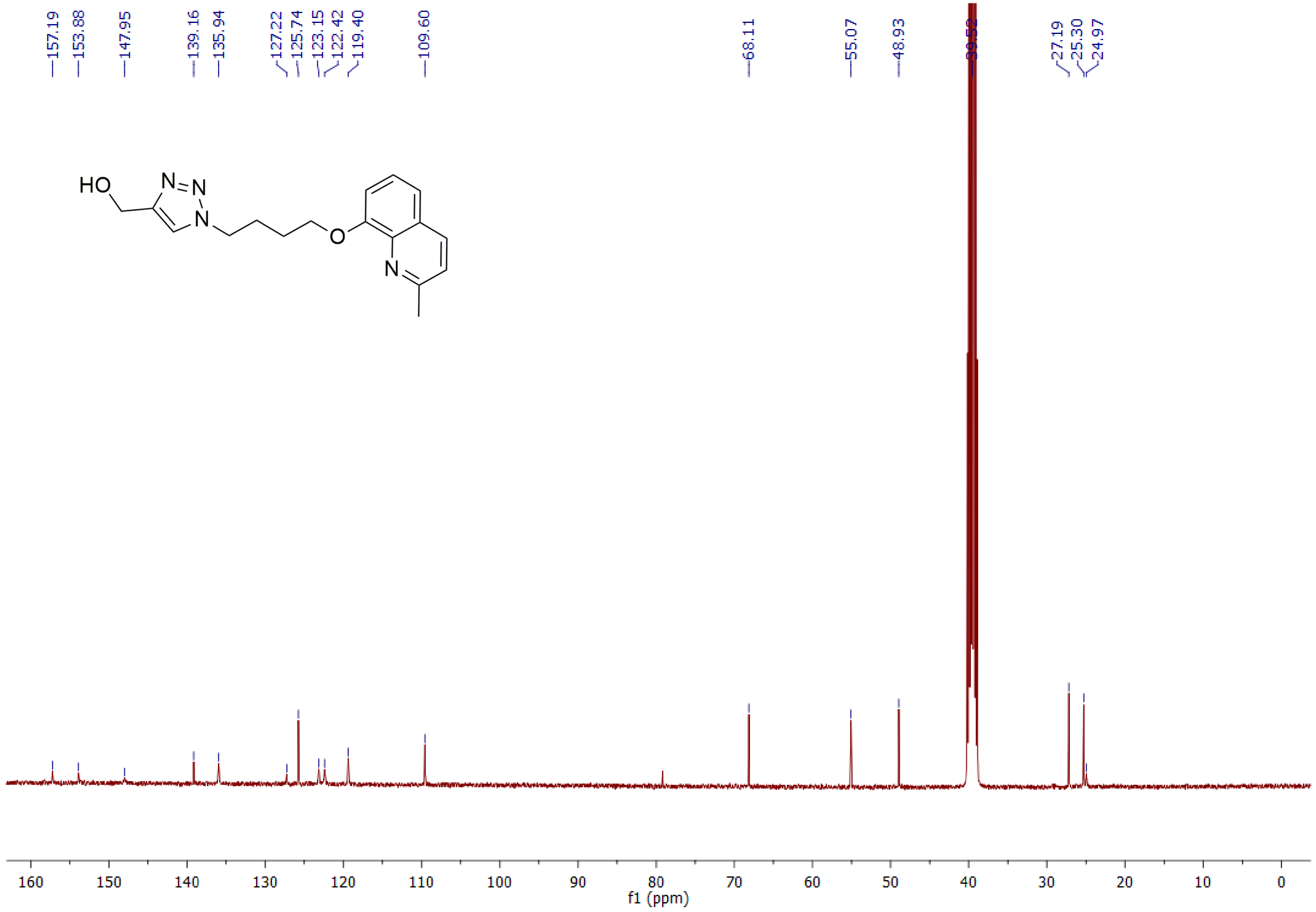

Figure S16. ^{13}C NMR spectrum of compound **M8** (100 MHz/DMSO/TMS; δ (ppm).

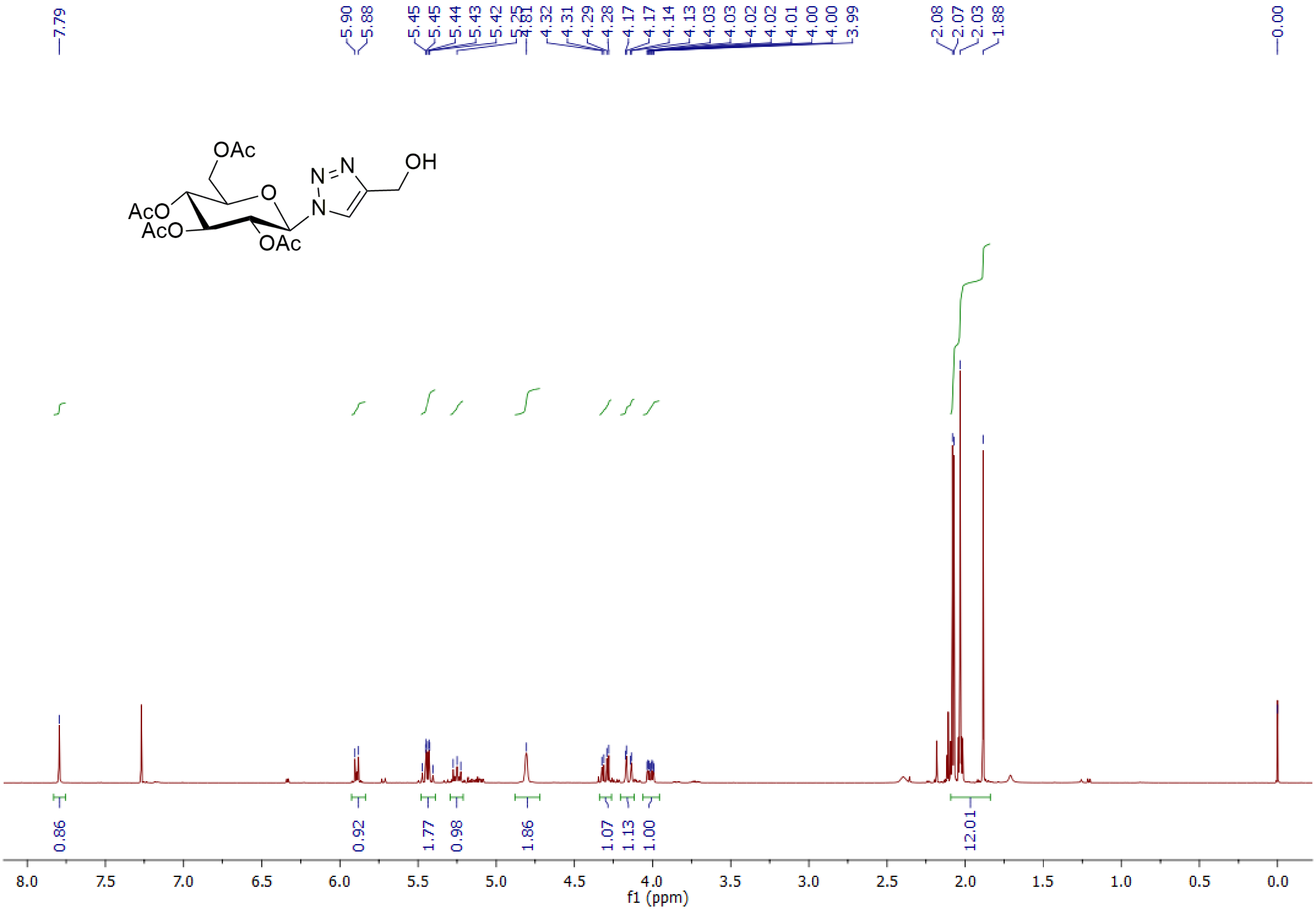

Figure S17. ^{1}H NMR spectrum of compound **M9** (400 MHz/$CDCl_3$/TMS; δ (ppm)).

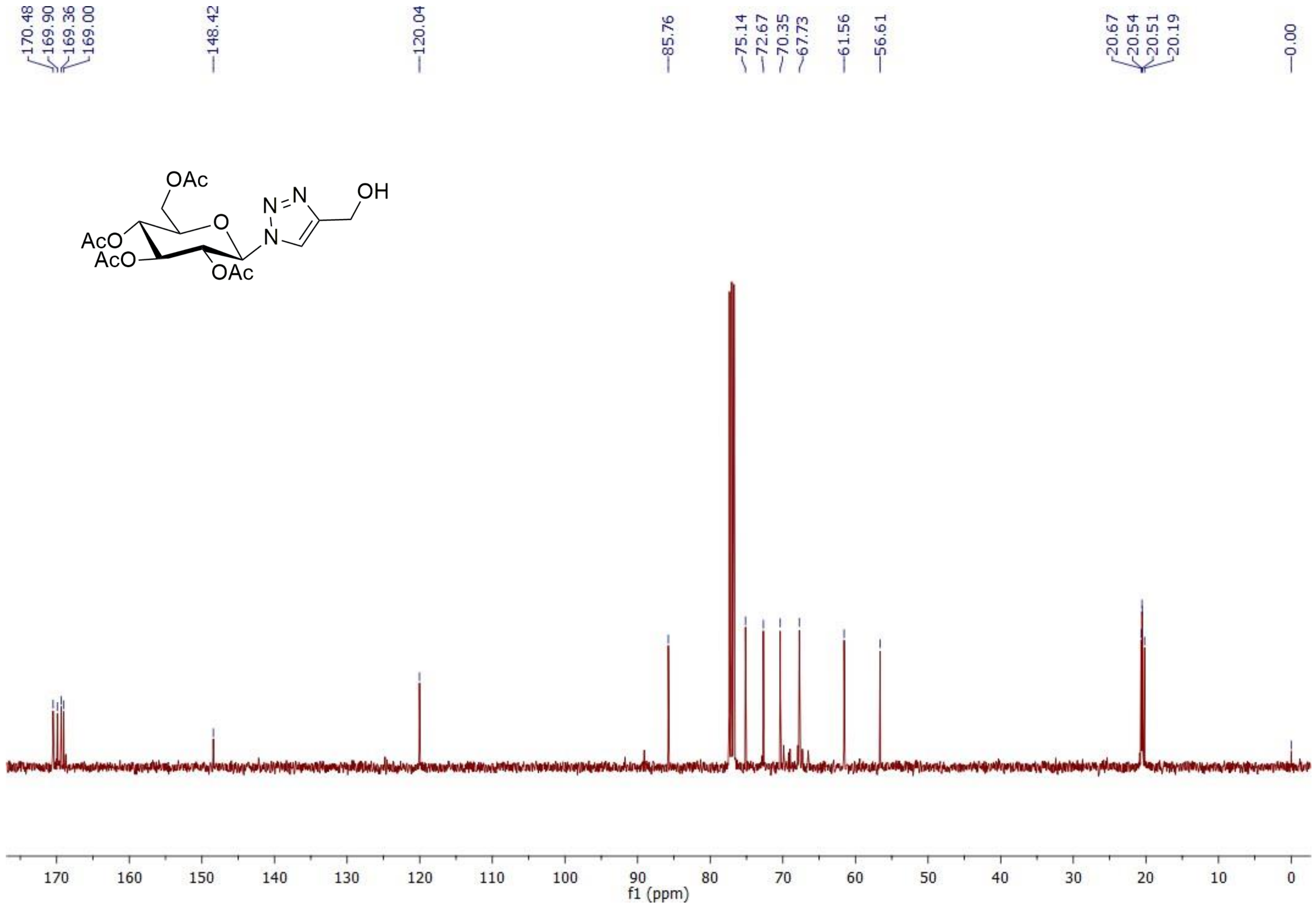

Figure S18. ^{13}C NMR spectrum of compound **M9** (100 MHz/$CDCl_3$/TMS; δ (ppm)).

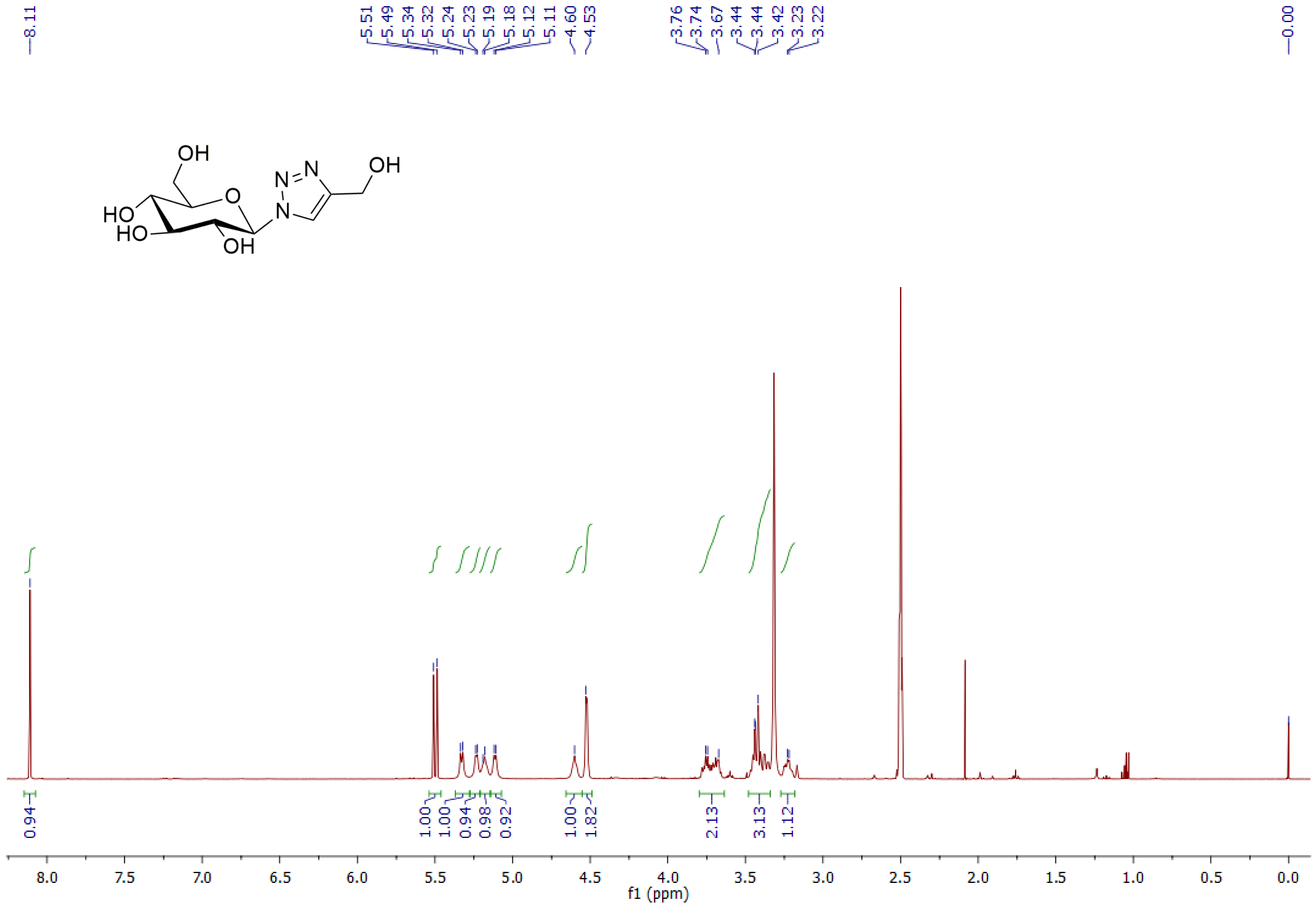

Figure S19. ^{1}H NMR spectrum of compound **M10** (400 MHz/DMSO/TMS; δ (ppm)).

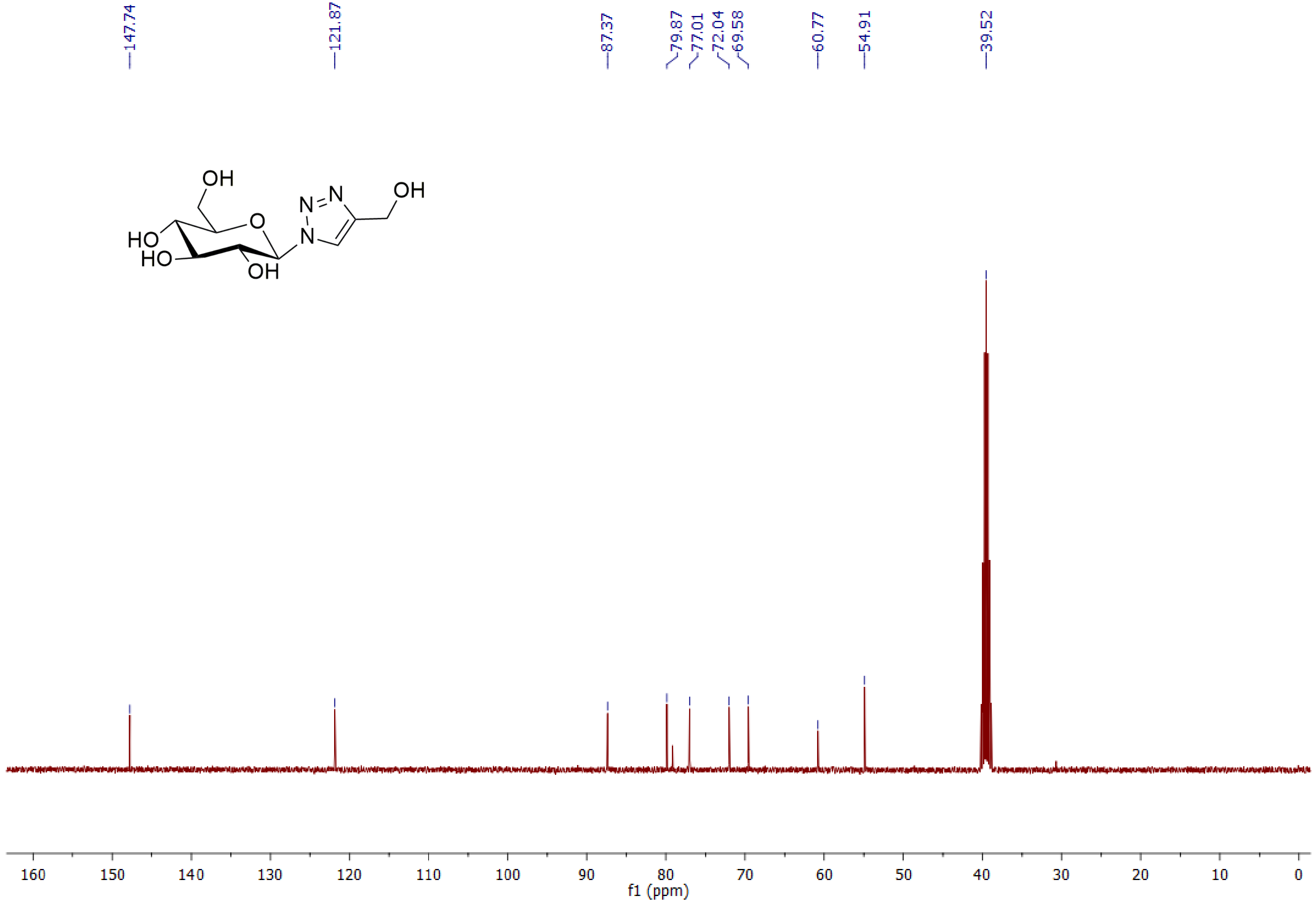

Figure S20. ^{13}C NMR spectrum of compound **M10** (100 MHz/DMSO/TMS; δ (ppm)).

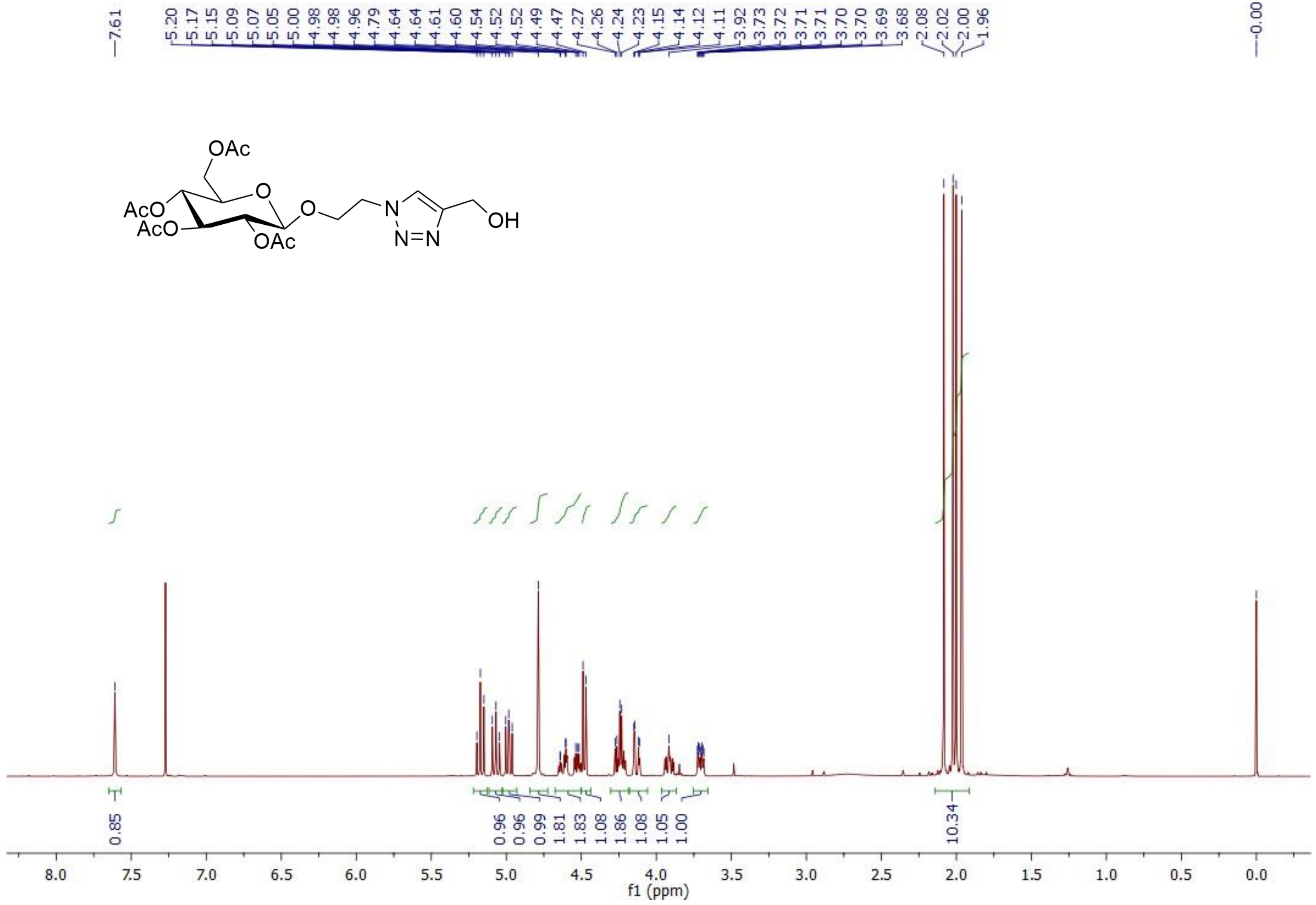

Figure S21. ^{1}H NMR spectrum of compound **M11** (400 MHz/$CDCl_3$/TMS; δ (ppm)).

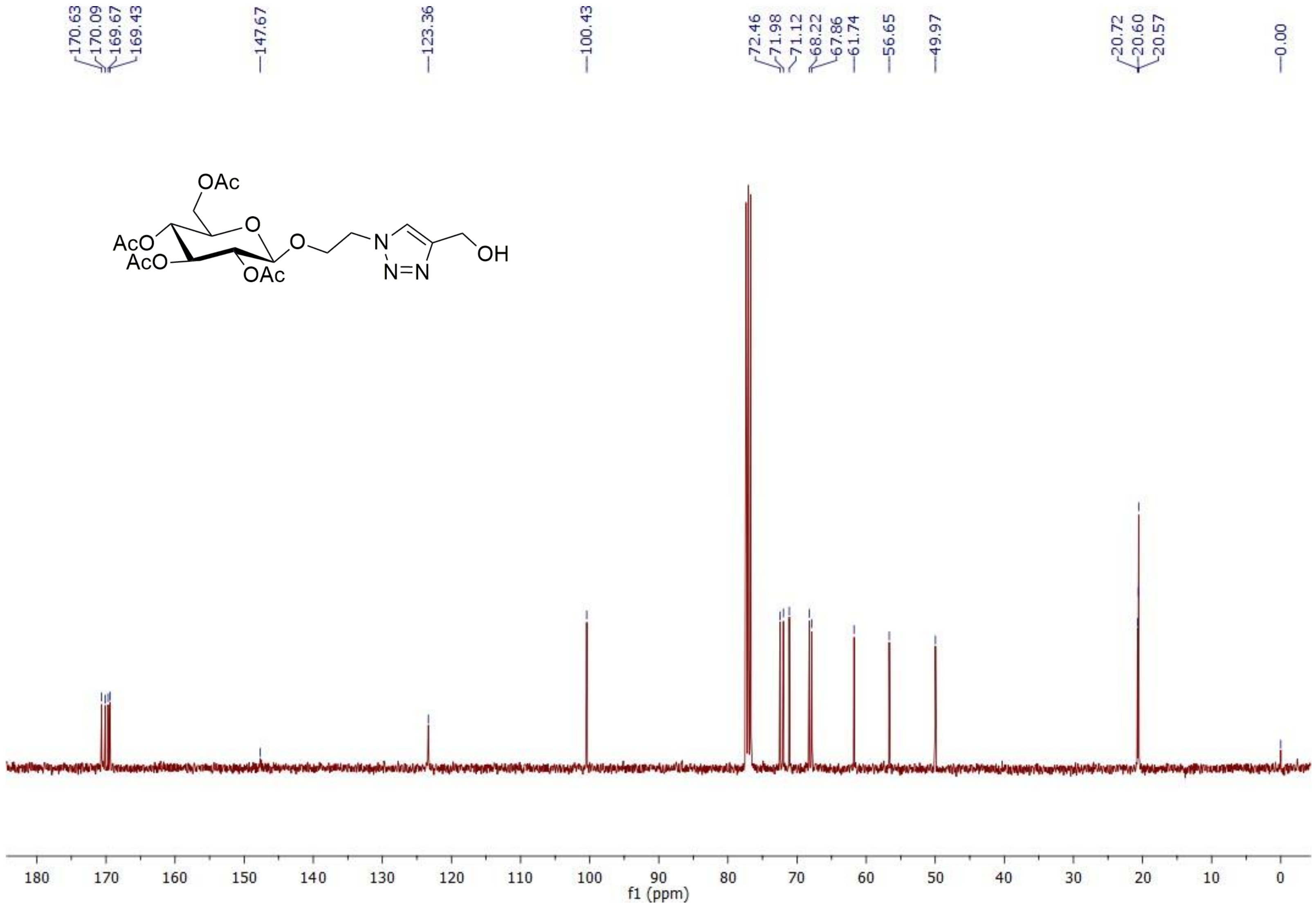

Figure S22. ^{13}C NMR spectrum of compound **M11** (100 MHz/$CDCl_3$/TMS; δ (ppm)).

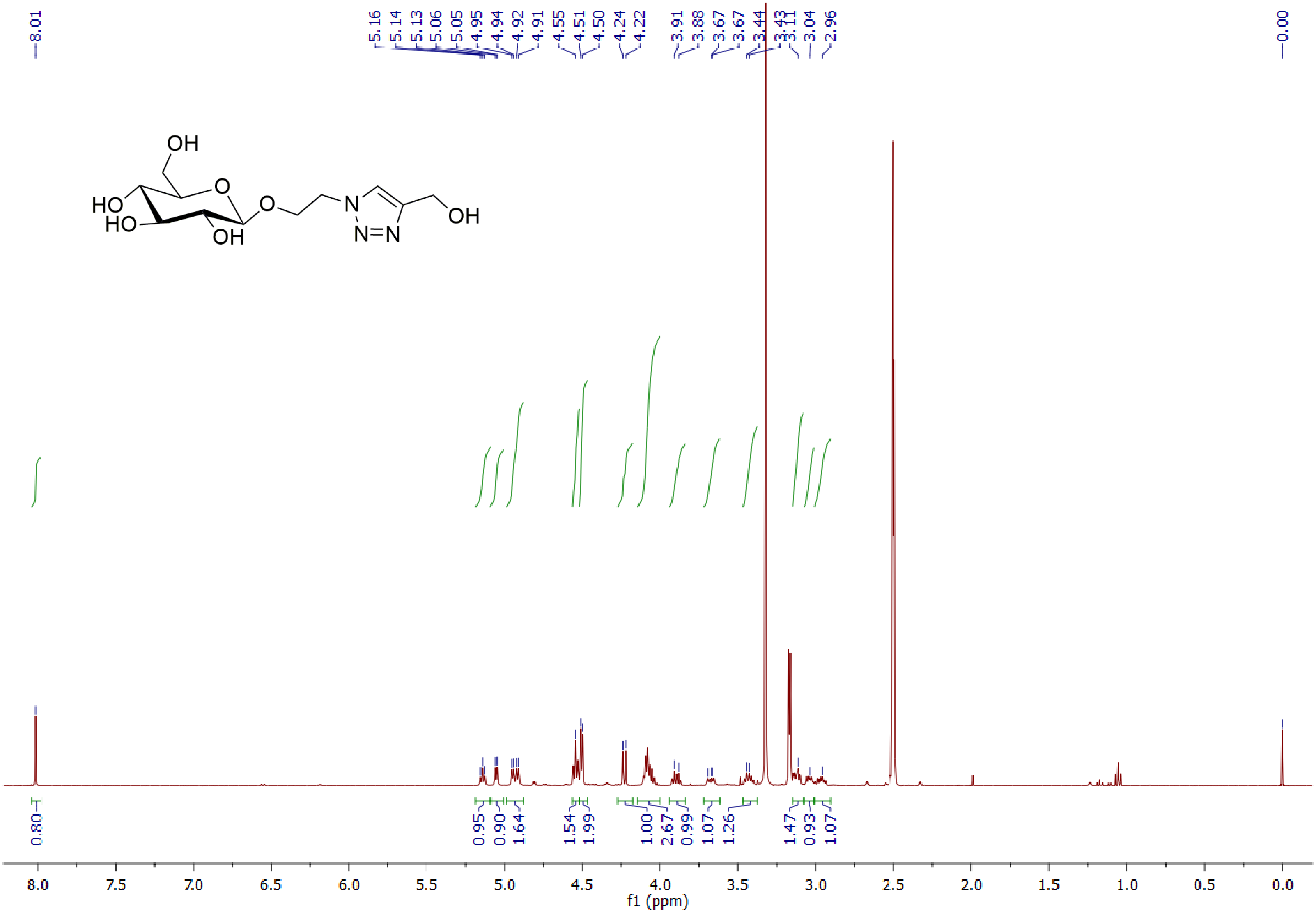

Figure S23. ^{1}H NMR spectrum of compound **M12** (400 MHz/DMSO/TMS; δ (ppm)).

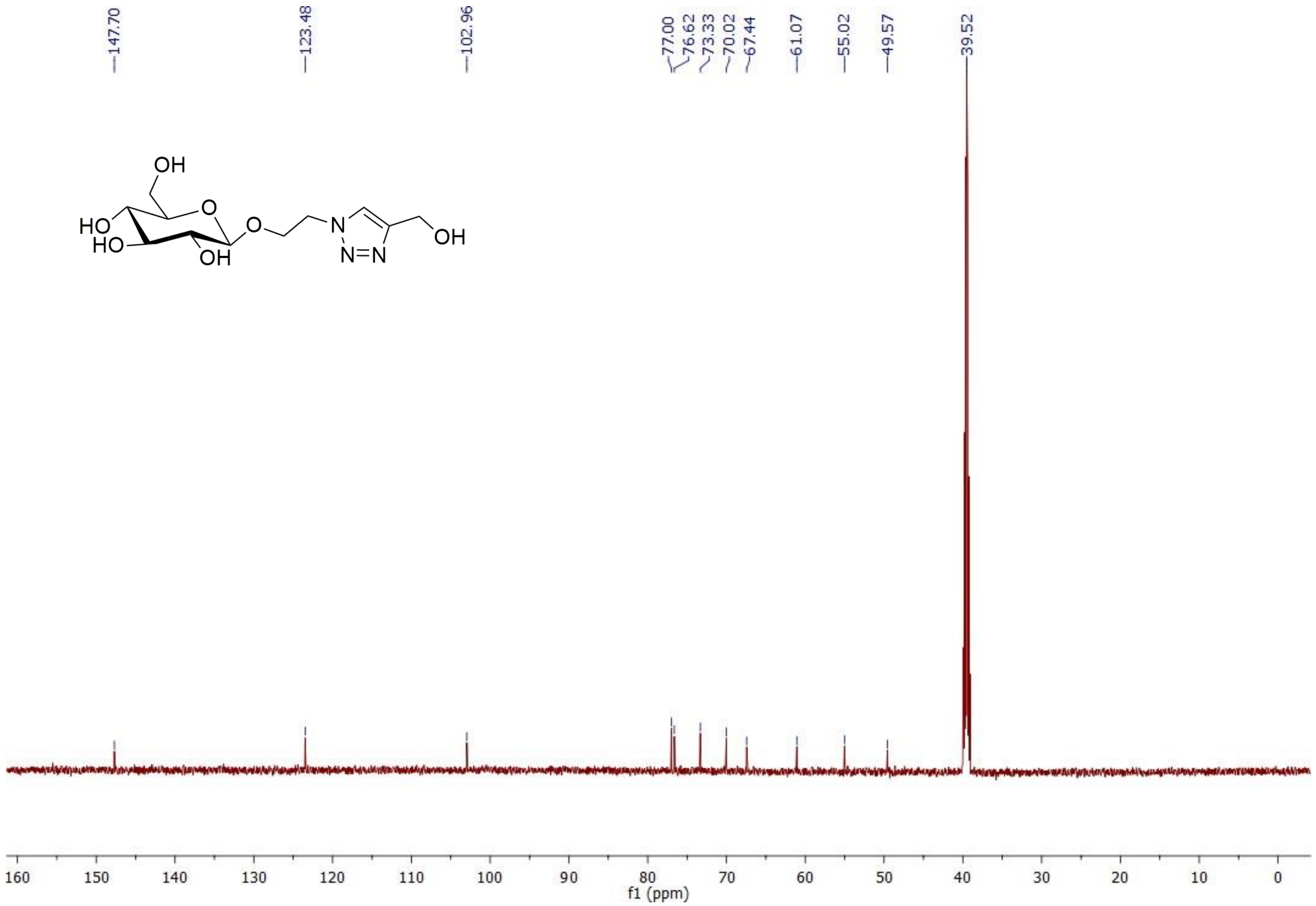

Figure S24. ^{13}C NMR spectrum of compound **M12** (100 MHz/DMSO/TMS; δ (ppm)).

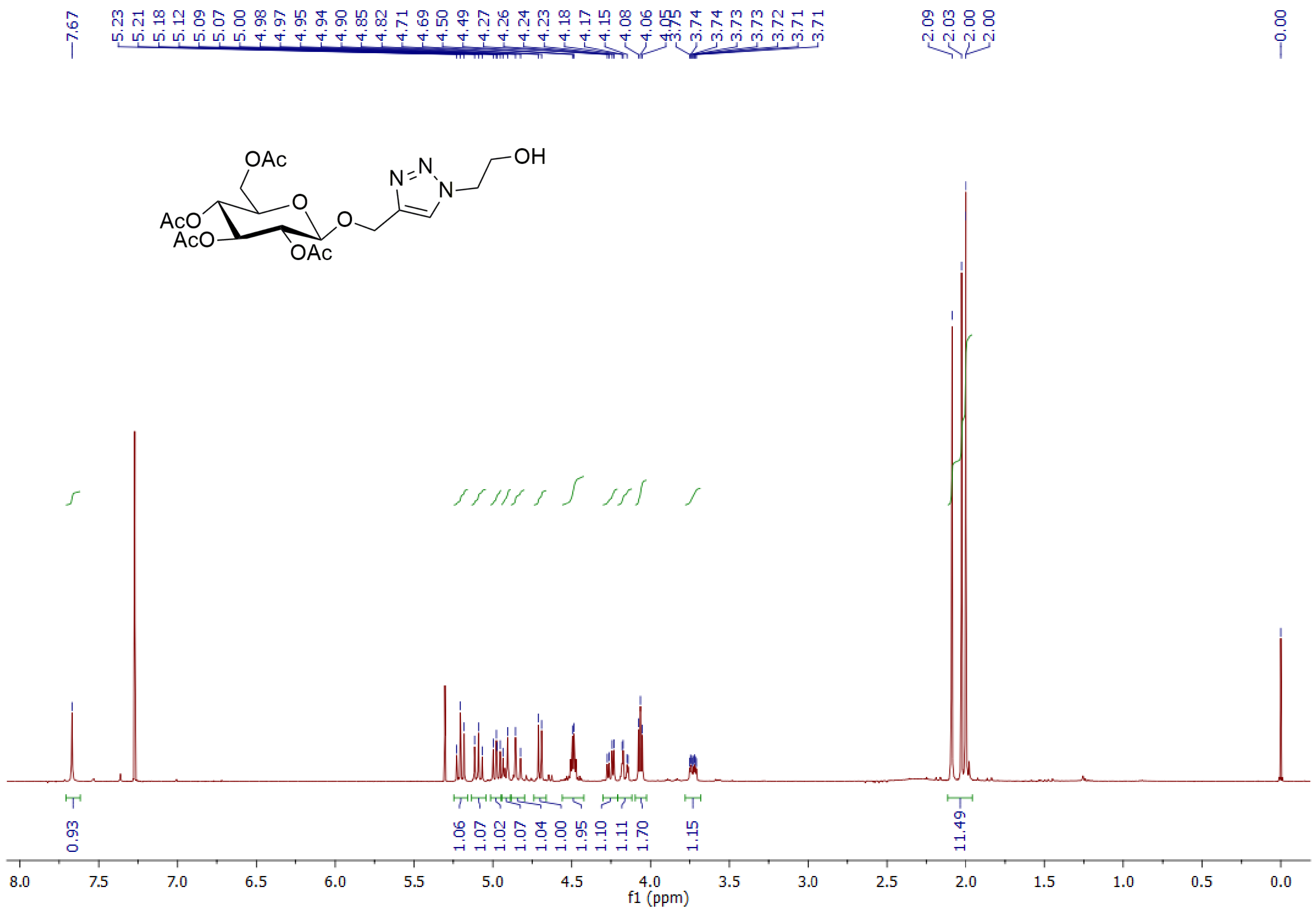

Figure S25. ^{1}H NMR spectrum of compound **M13** (400 MHz/$CDCl_3$/TMS; δ (ppm)).

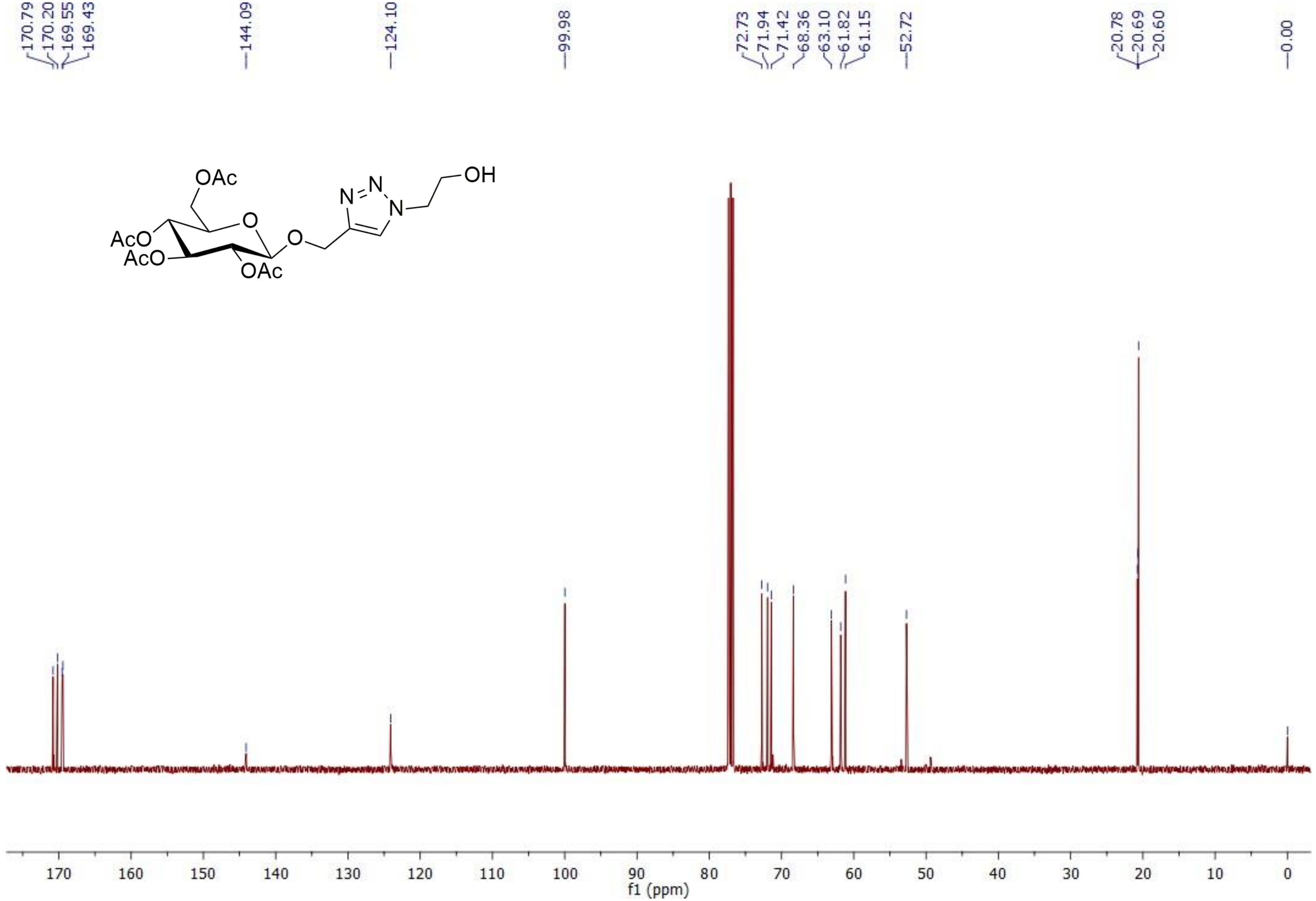

Figure S26. ^{13}C NMR spectrum of compound **M13** (100 MHz/$CDCl_3$/TMS; δ (ppm)).

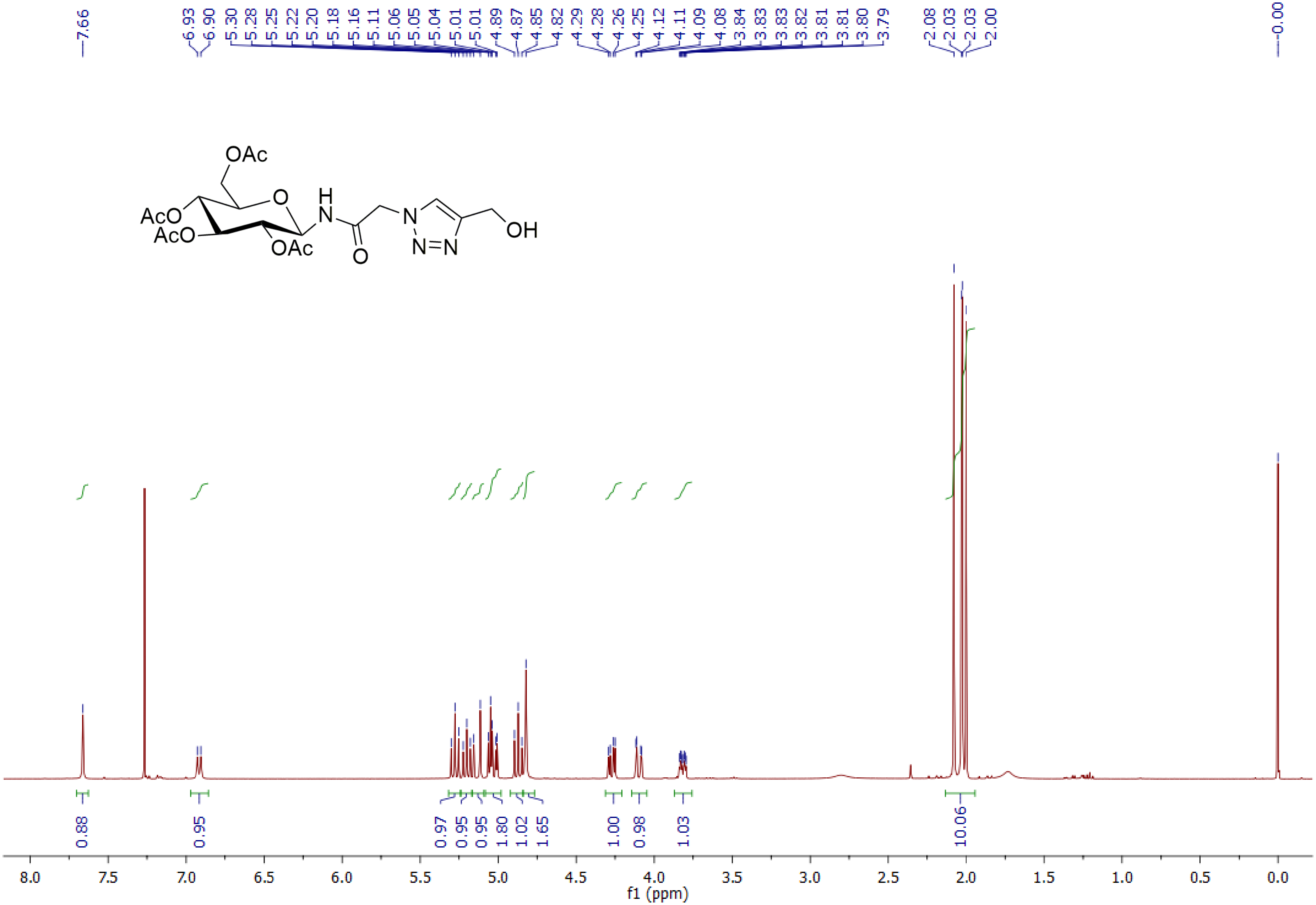

Figure S27. ^{1}H NMR spectrum of compound **M14** (400 MHz/$CDCl_3$/TMS; δ (ppm)).

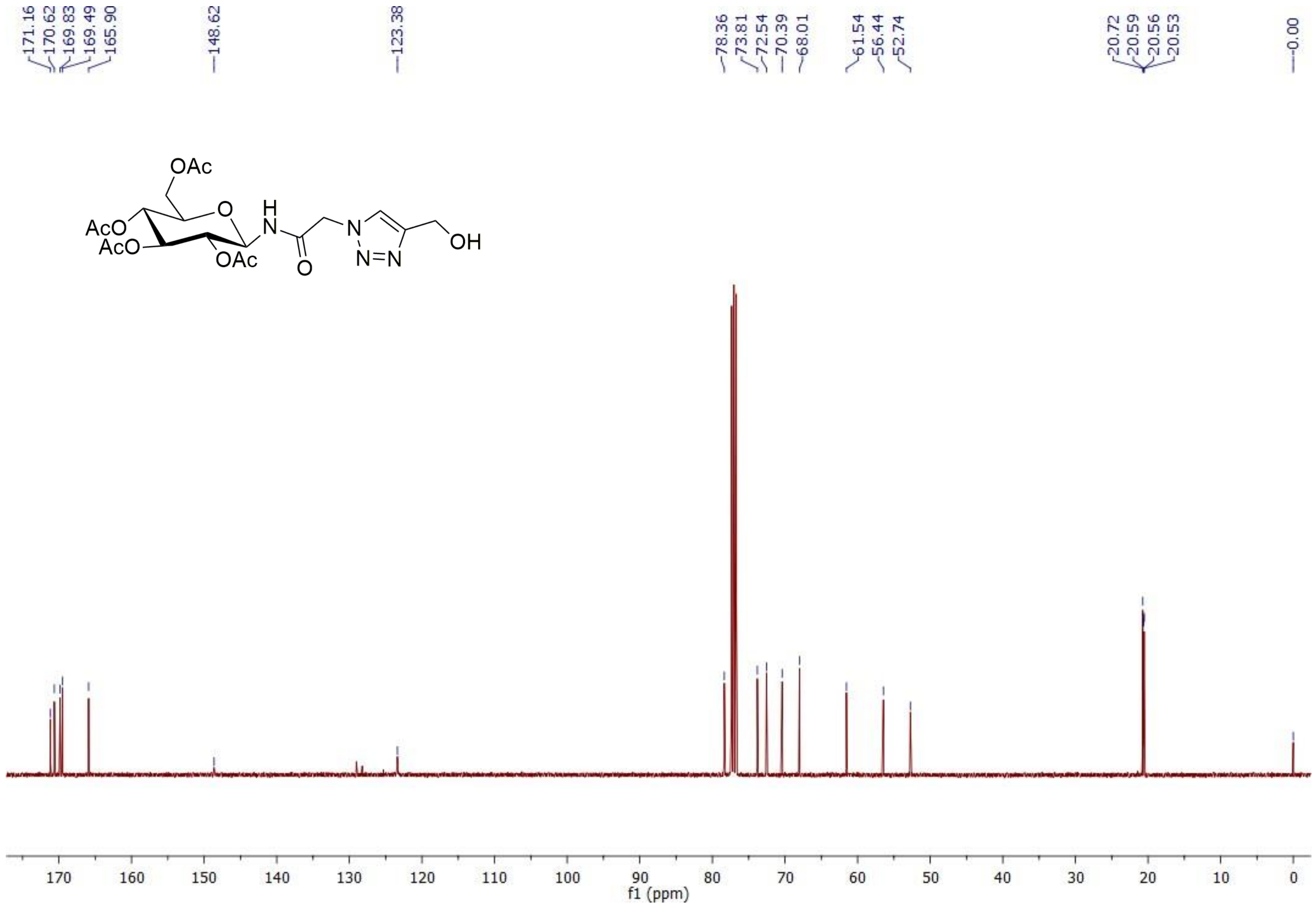

Figure S28. ^{13}C NMR spectrum of compound **M14** (100 MHz/$CDCl_3$/TMS; δ (ppm)).

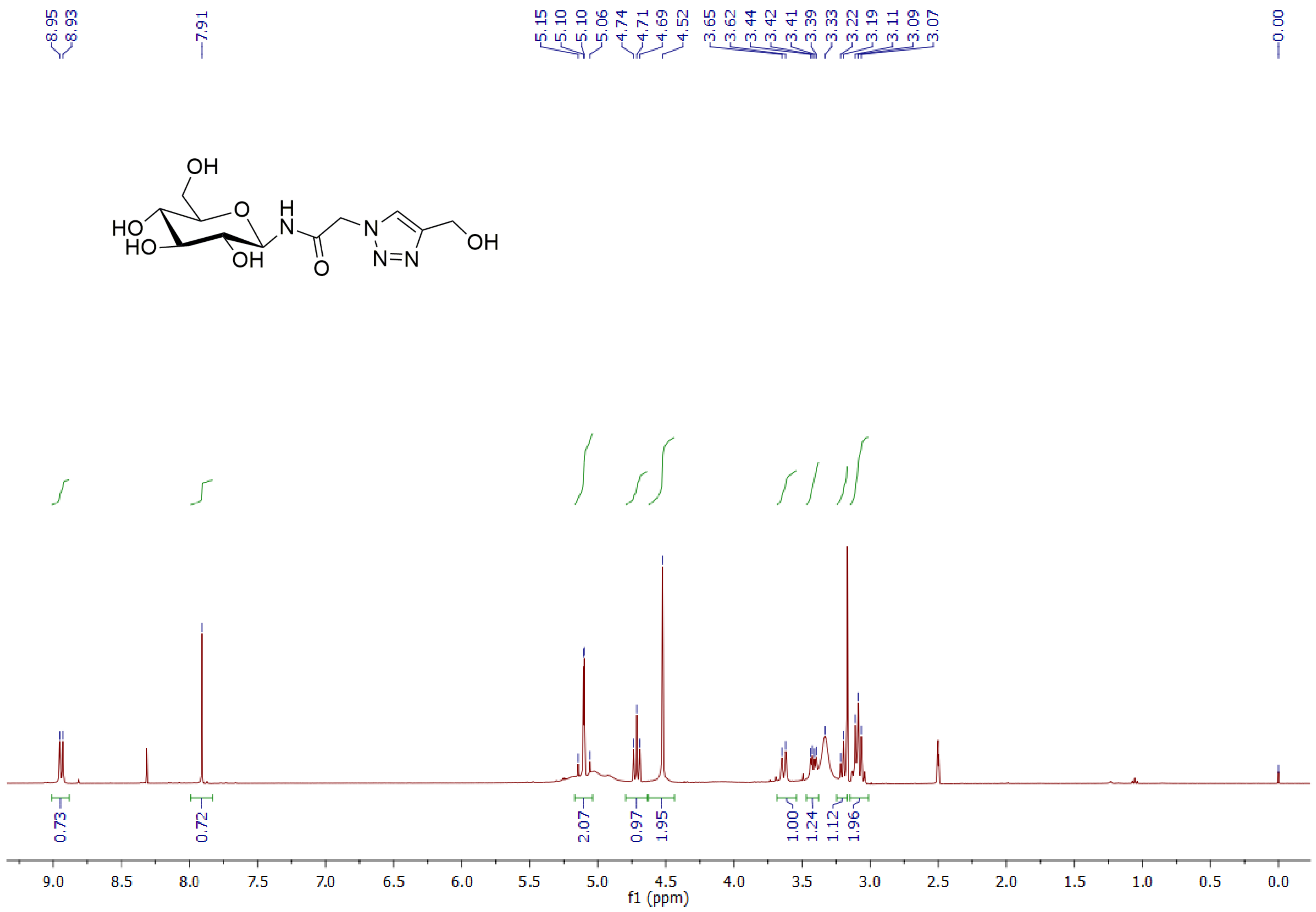

Figure S29. ^{1}H NMR spectrum of compound **M15** (400 MHz/DMSO/TMS; δ (ppm)).

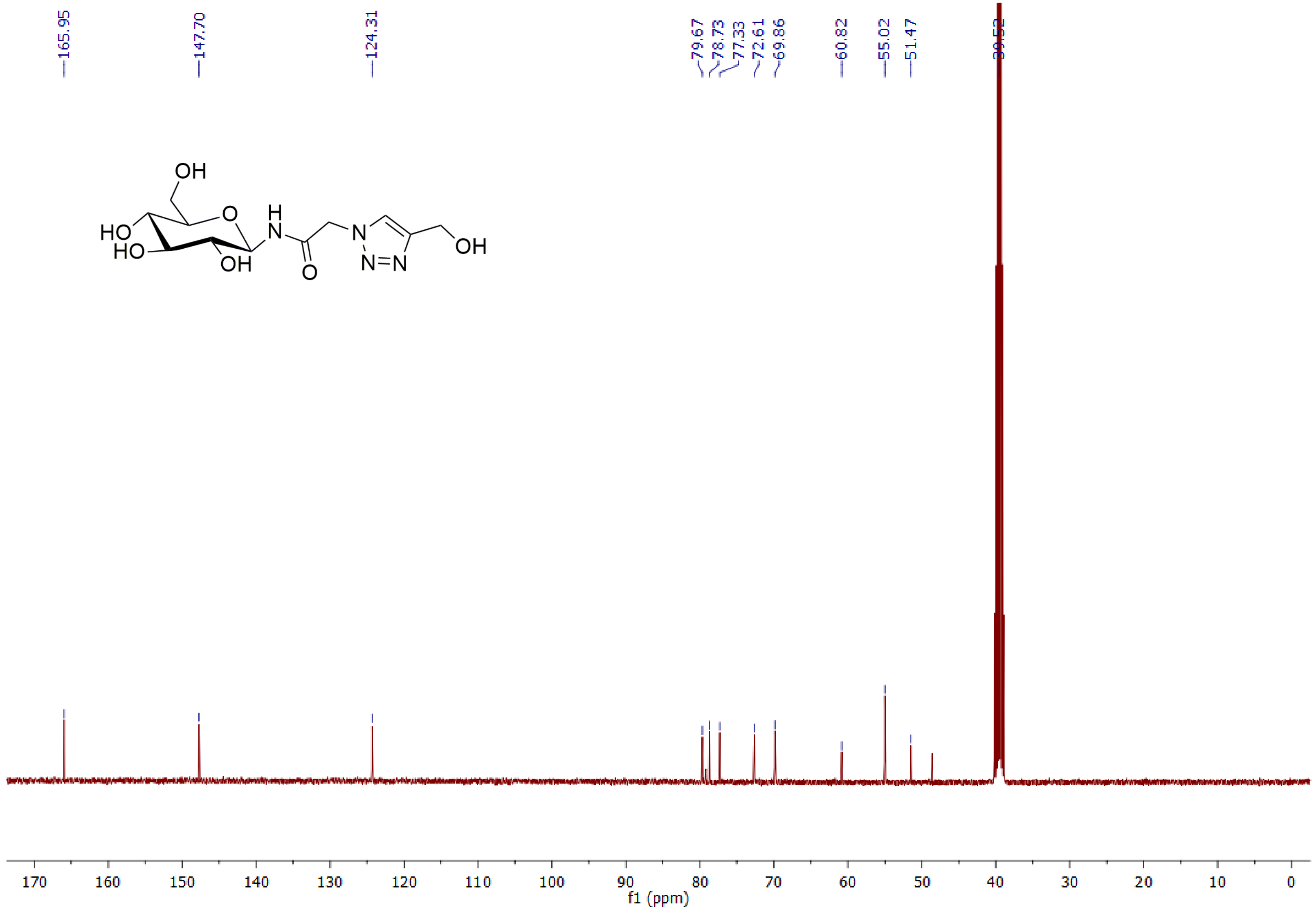

Figure S30. ^{13}C NMR spectrum of compound **M15** (100 MHz/DMSO/TMS; δ (ppm)).

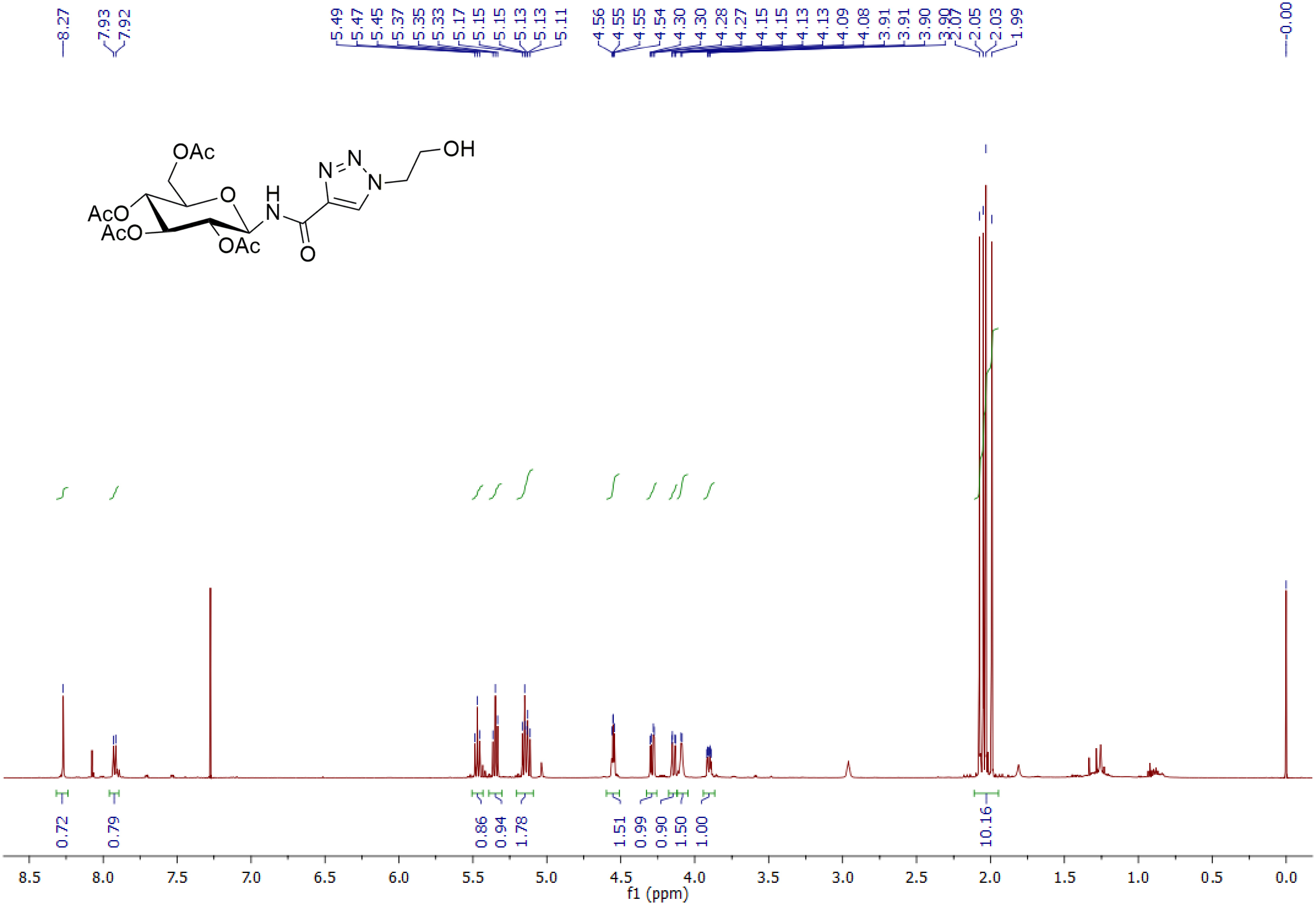

Figure S31. ^{1}H NMR spectrum of compound **M16** (600 MHz/$CDCl_3$/TMS; δ (ppm)).

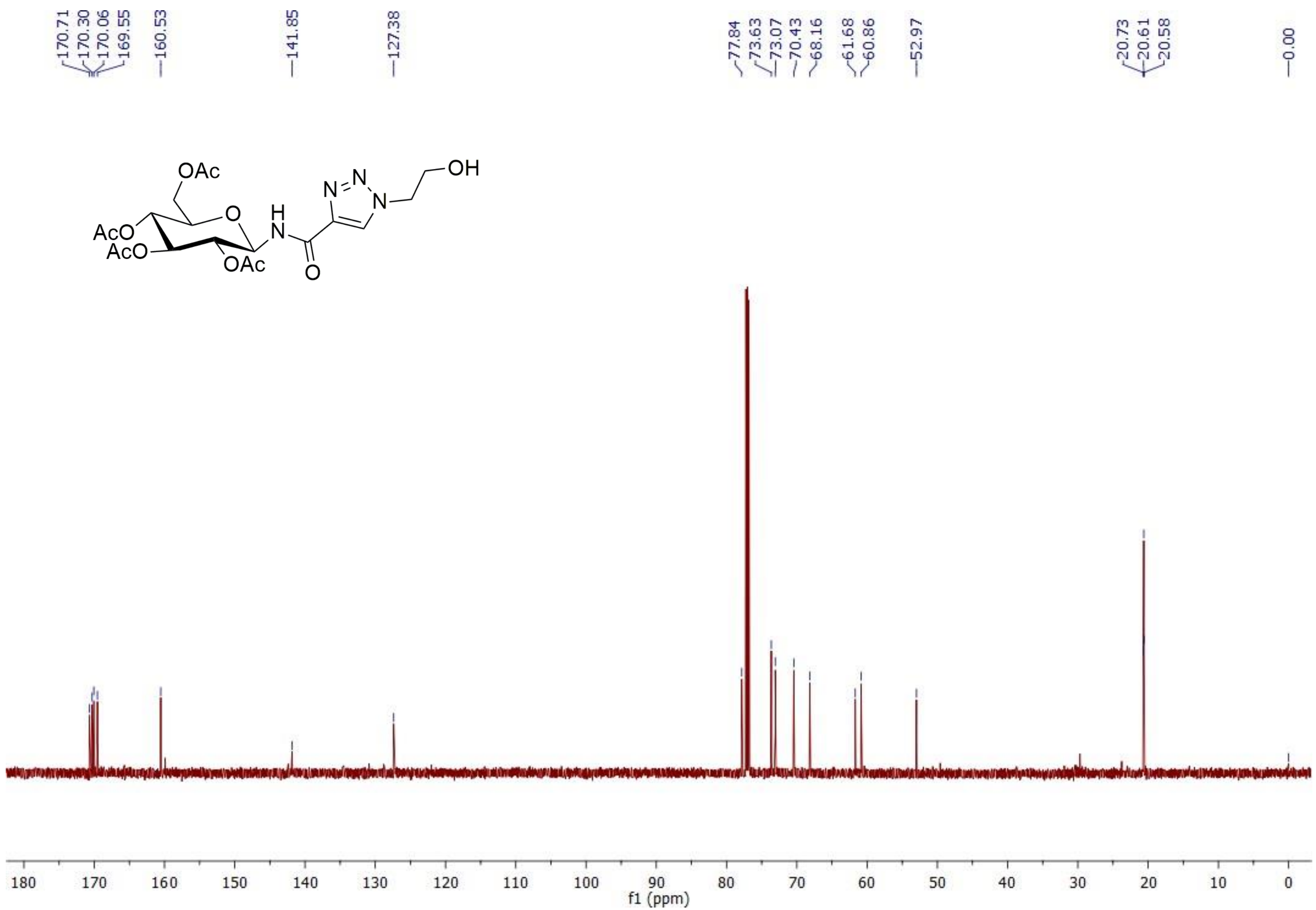

Figure S32. ^{13}C NMR spectrum of compound **M16** (150 MHz/$CDCl_3$/TMS; δ (ppm)).

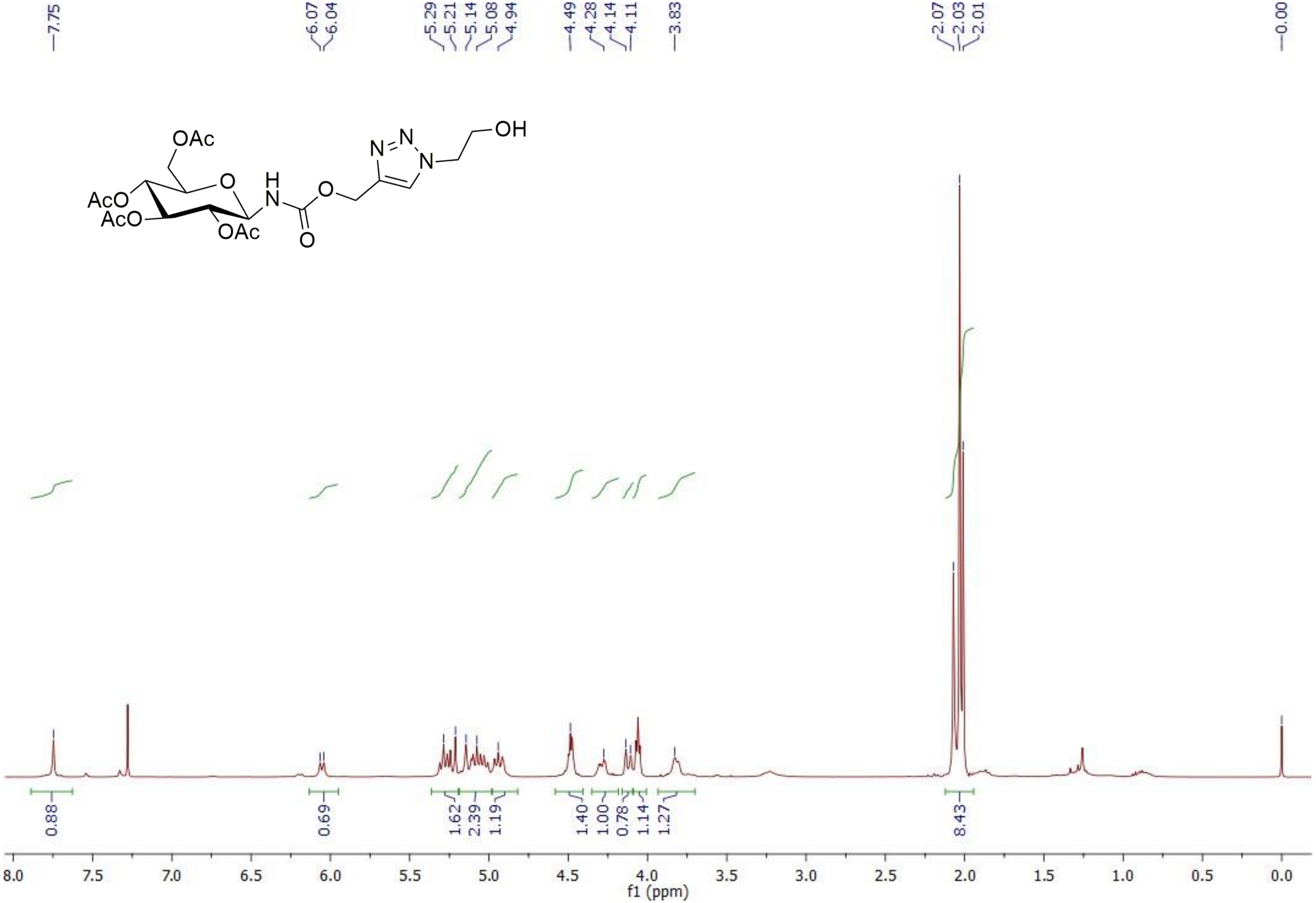

Figure S33. ^{1}H NMR spectrum of compound **M17** (400 MHz/$CDCl_3$/TMS; δ (ppm)).

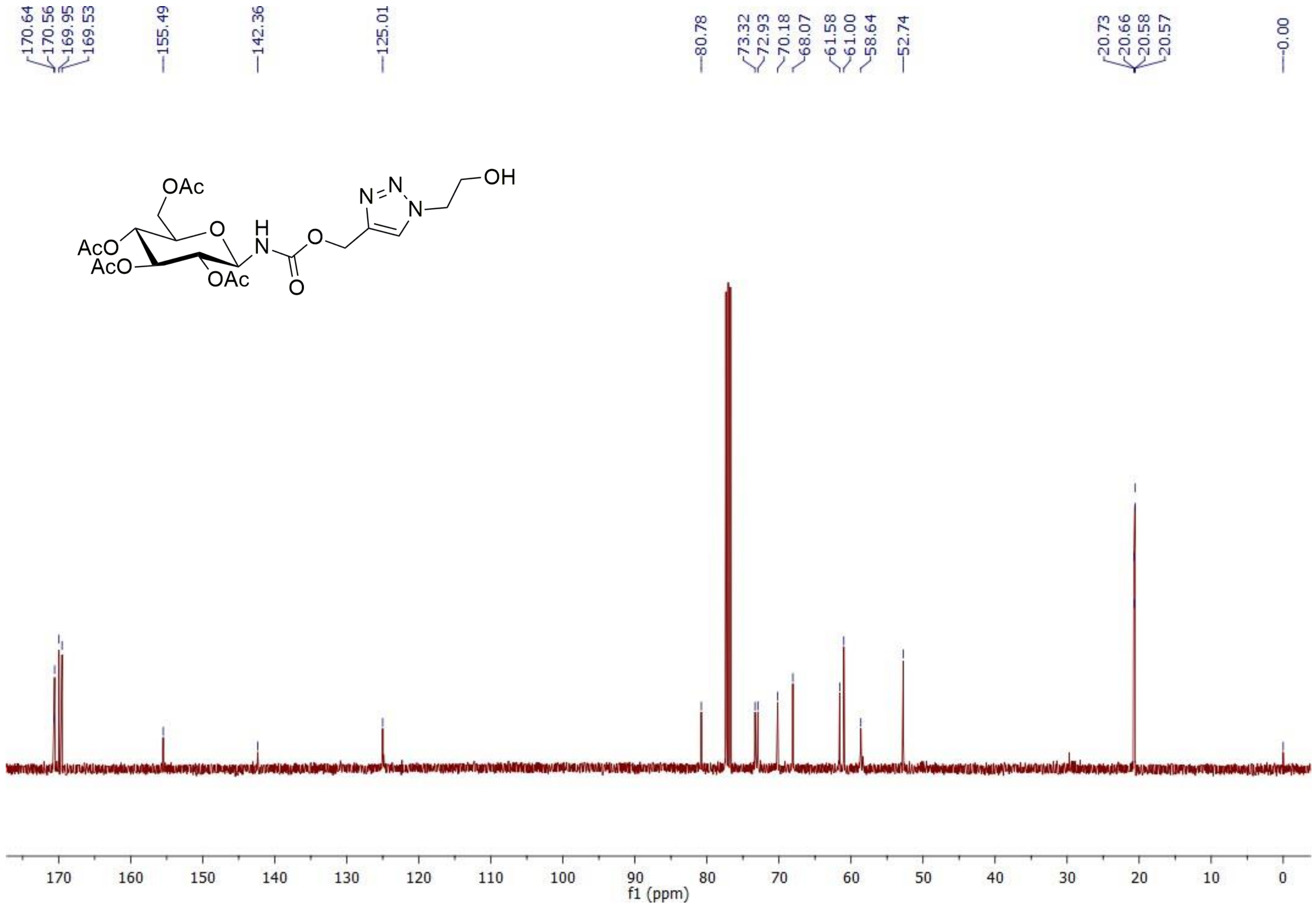

Figure S34. ^{13}C NMR spectrum of compound **M17** (100 MHz/$CDCl_3$/TMS; δ (ppm)).

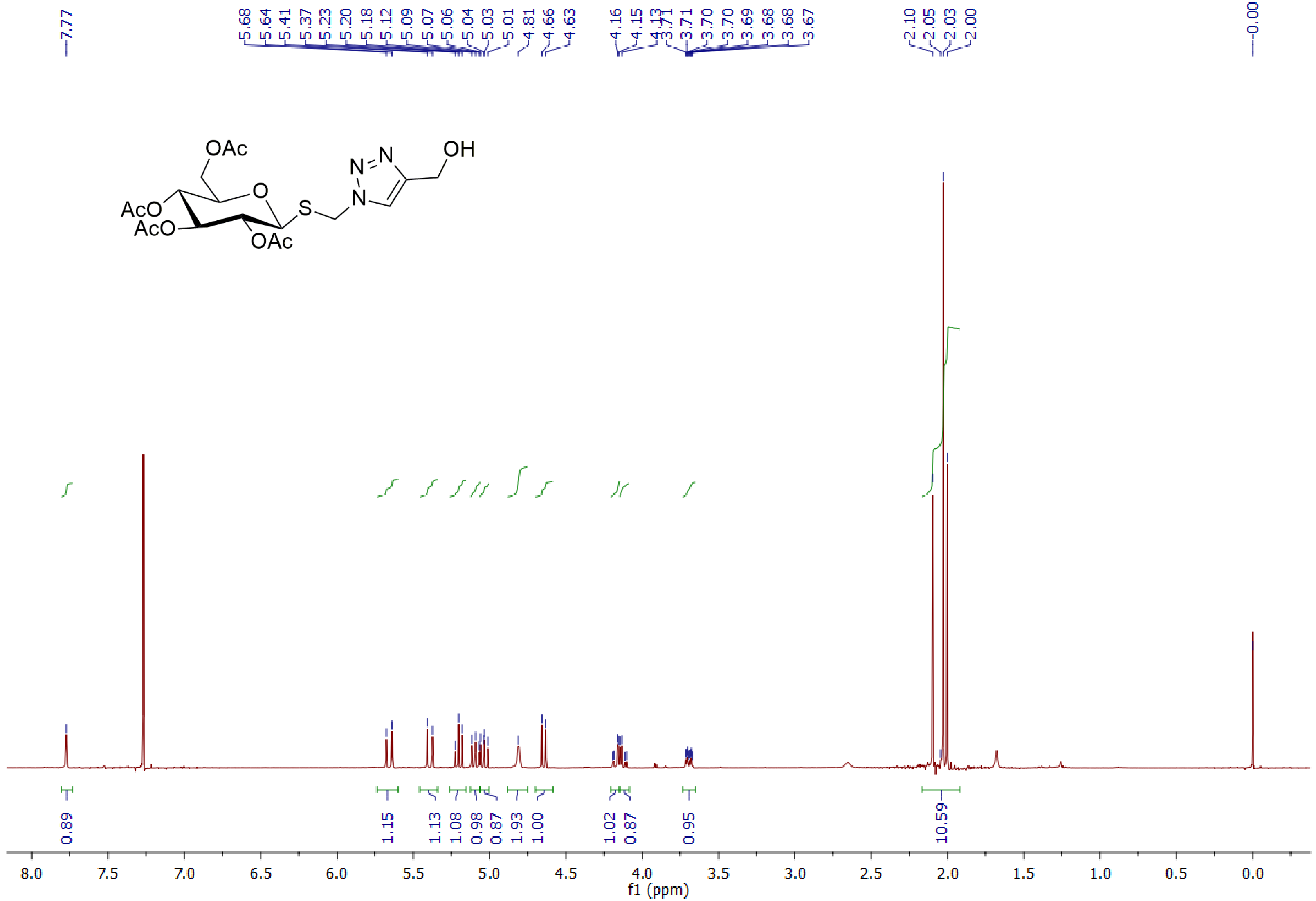

Figure S35. ^{1}H NMR spectrum of compound **M18** (400 MHz/$CDCl_3$/TMS; δ (ppm)).

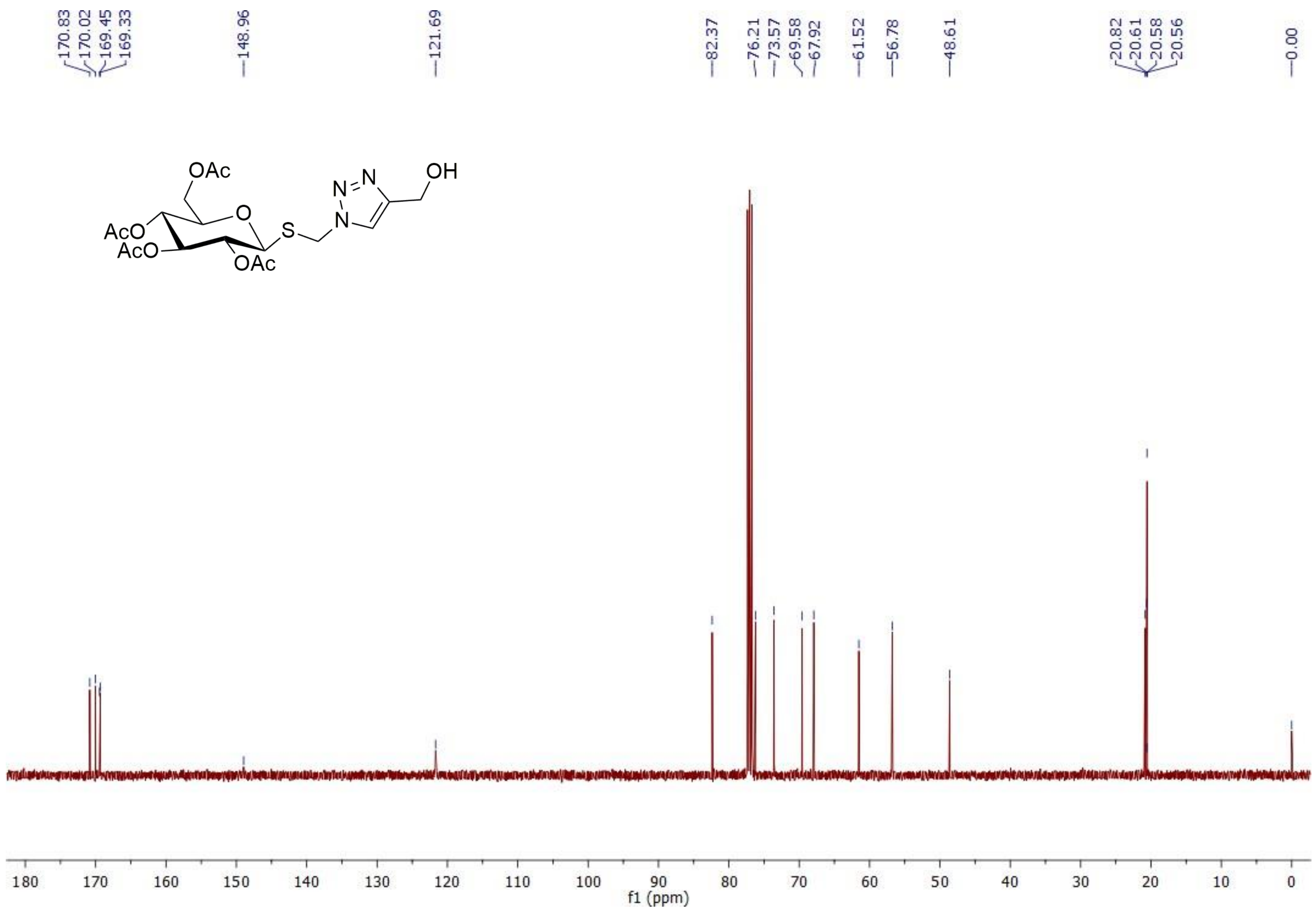

Figure S36. ^{13}C NMR spectrum of compound **M18** (100 MHz/$CDCl_3$/TMS; δ (ppm)).

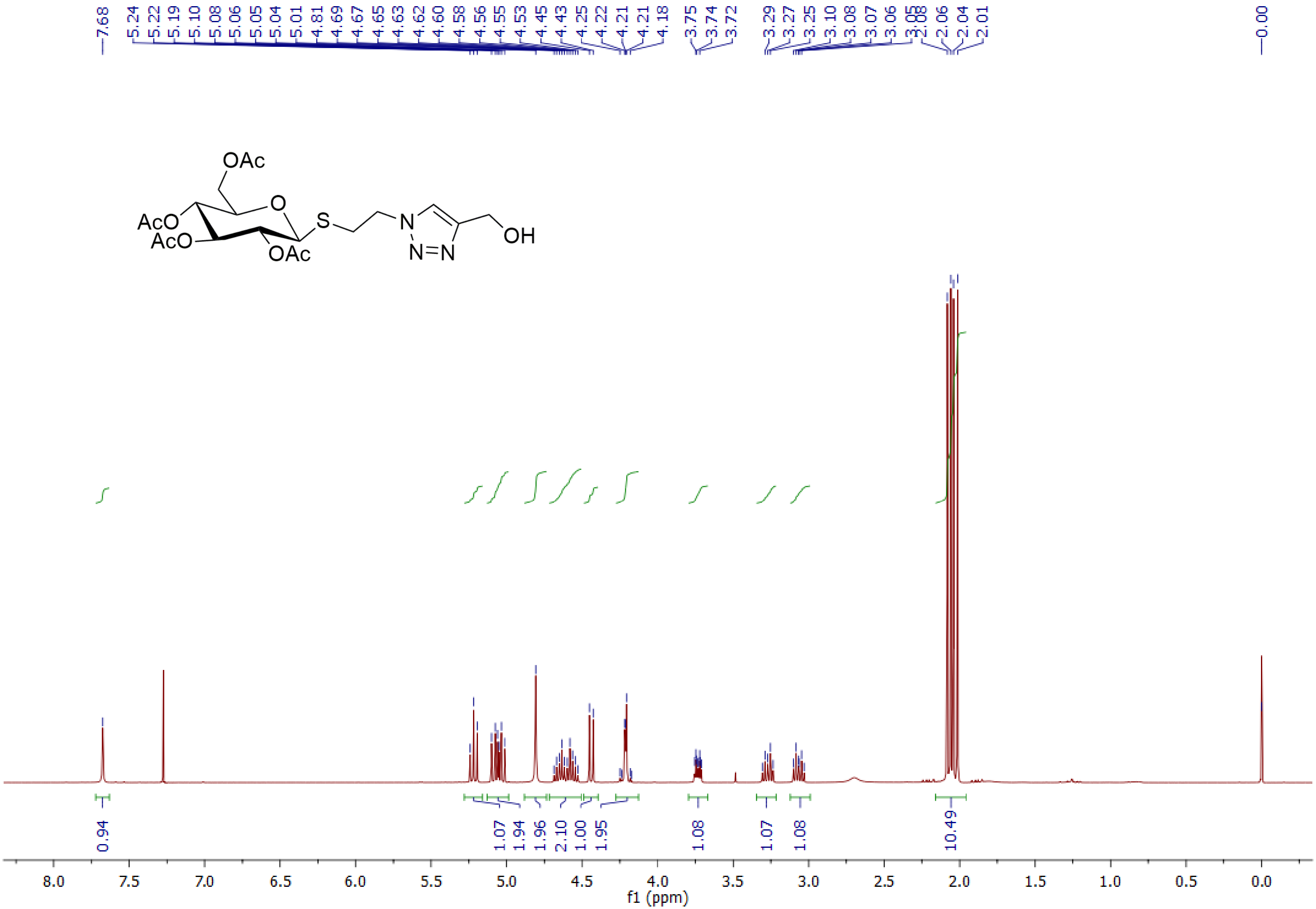

Figure S37. ^1H NMR spectrum of compound **M18** (400 MHz/$CDCl_3$/TMS; δ (ppm)).

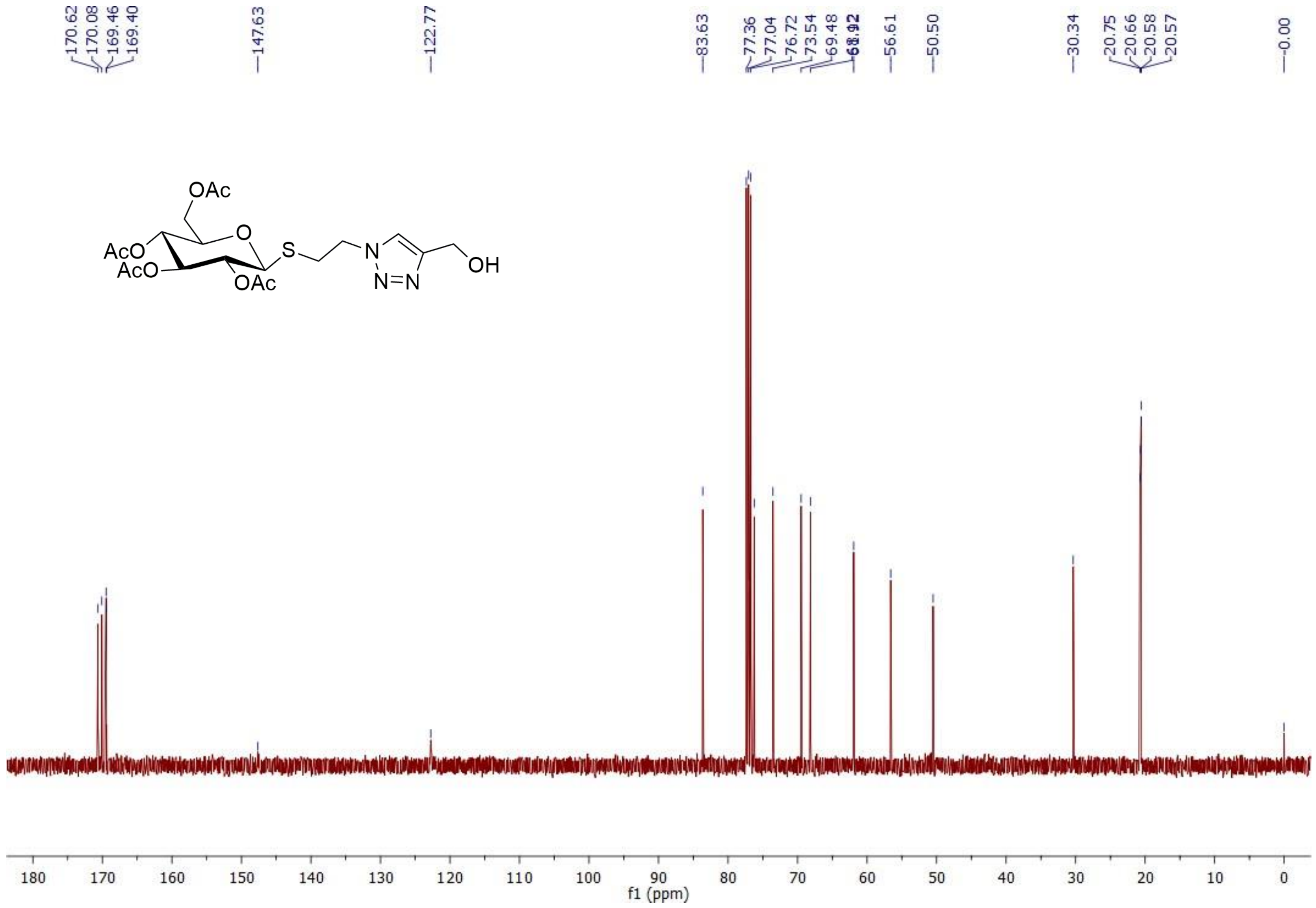

Figure S38. ^{13}C NMR spectrum of compound **M18** (100 MHz/$CDCl_3$/TMS; δ (ppm)).

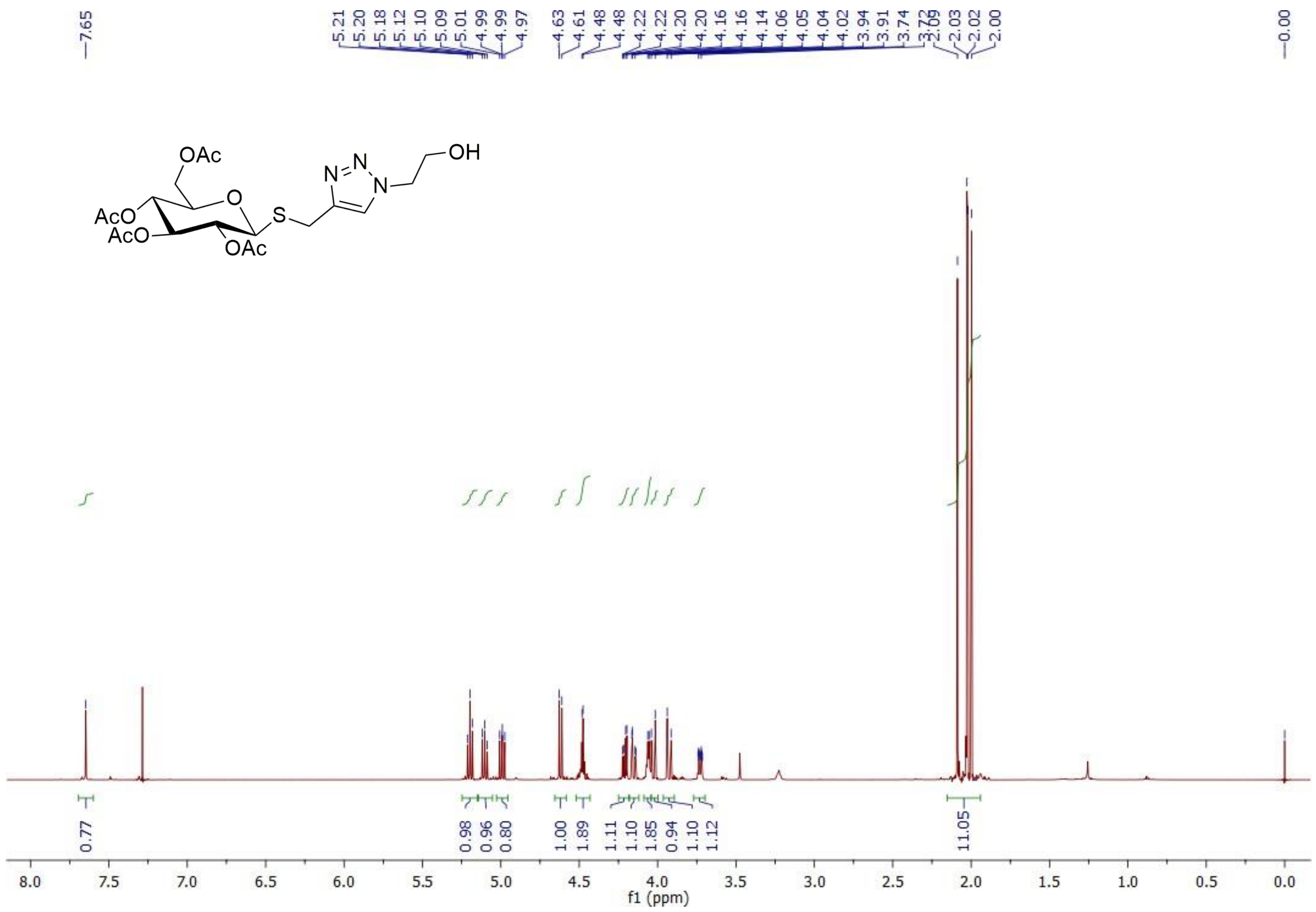

Figure S39. ^{1}H NMR spectrum of compound **M20** (600 MHz/$CDCl_3$/TMS; δ (ppm)).

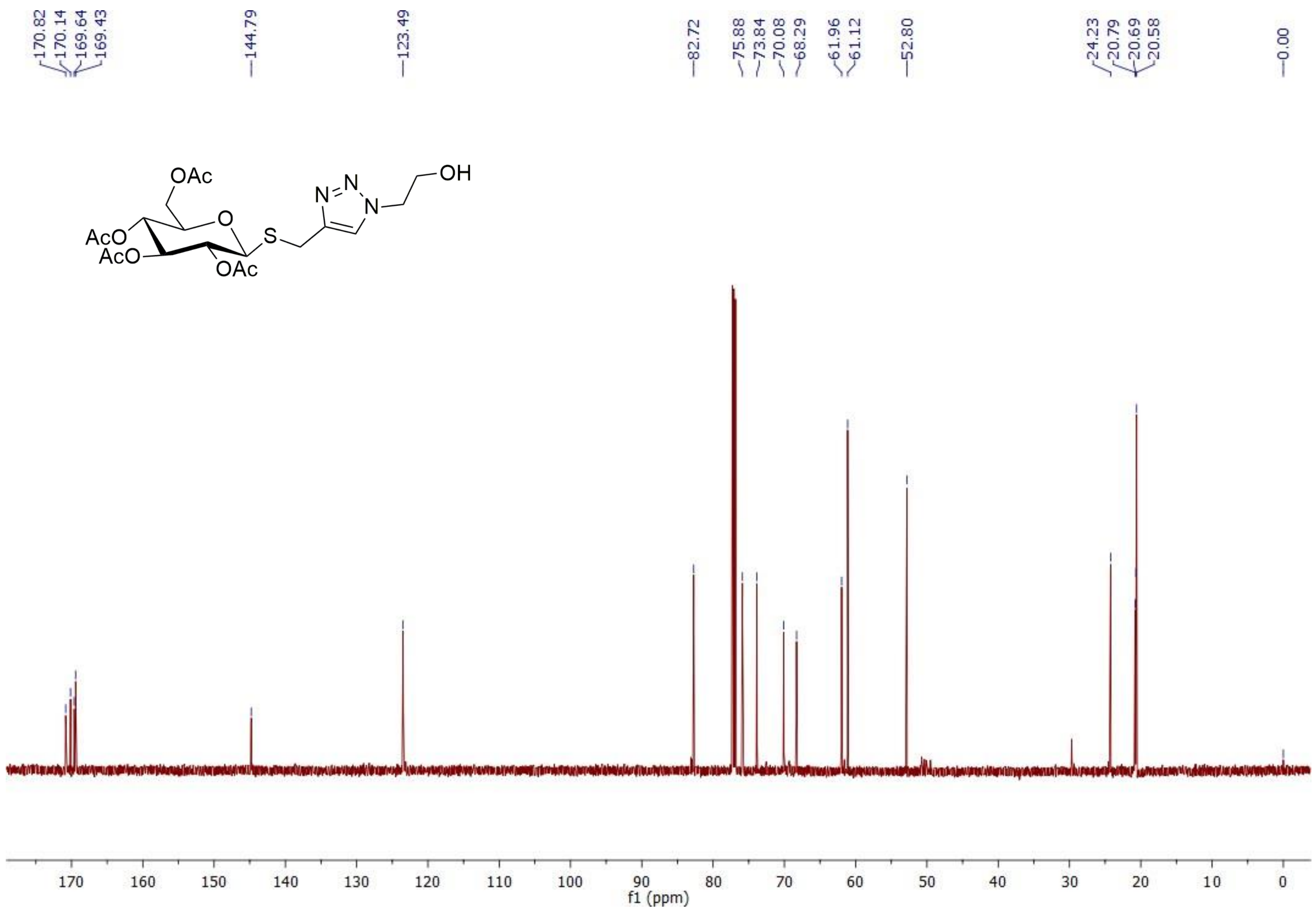

Figure S40. ^{13}C NMR spectrum of compound **M20** (150 MHz/$CDCl_3$/TMS; δ (ppm)).

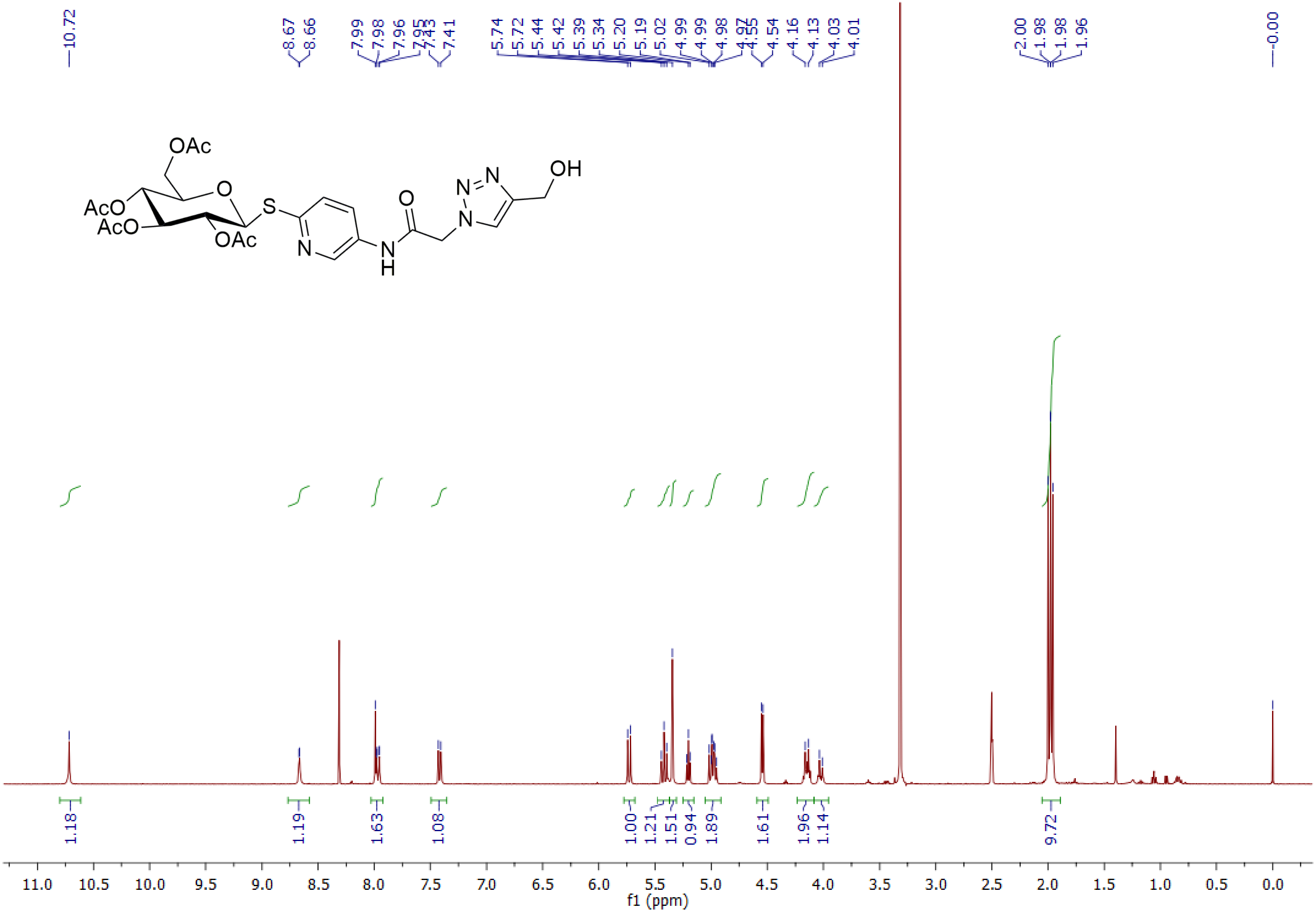

Figure S41. ^{1}H NMR spectrum of compound **M21** (400 MHz/DMSO/TMS; δ (ppm)).

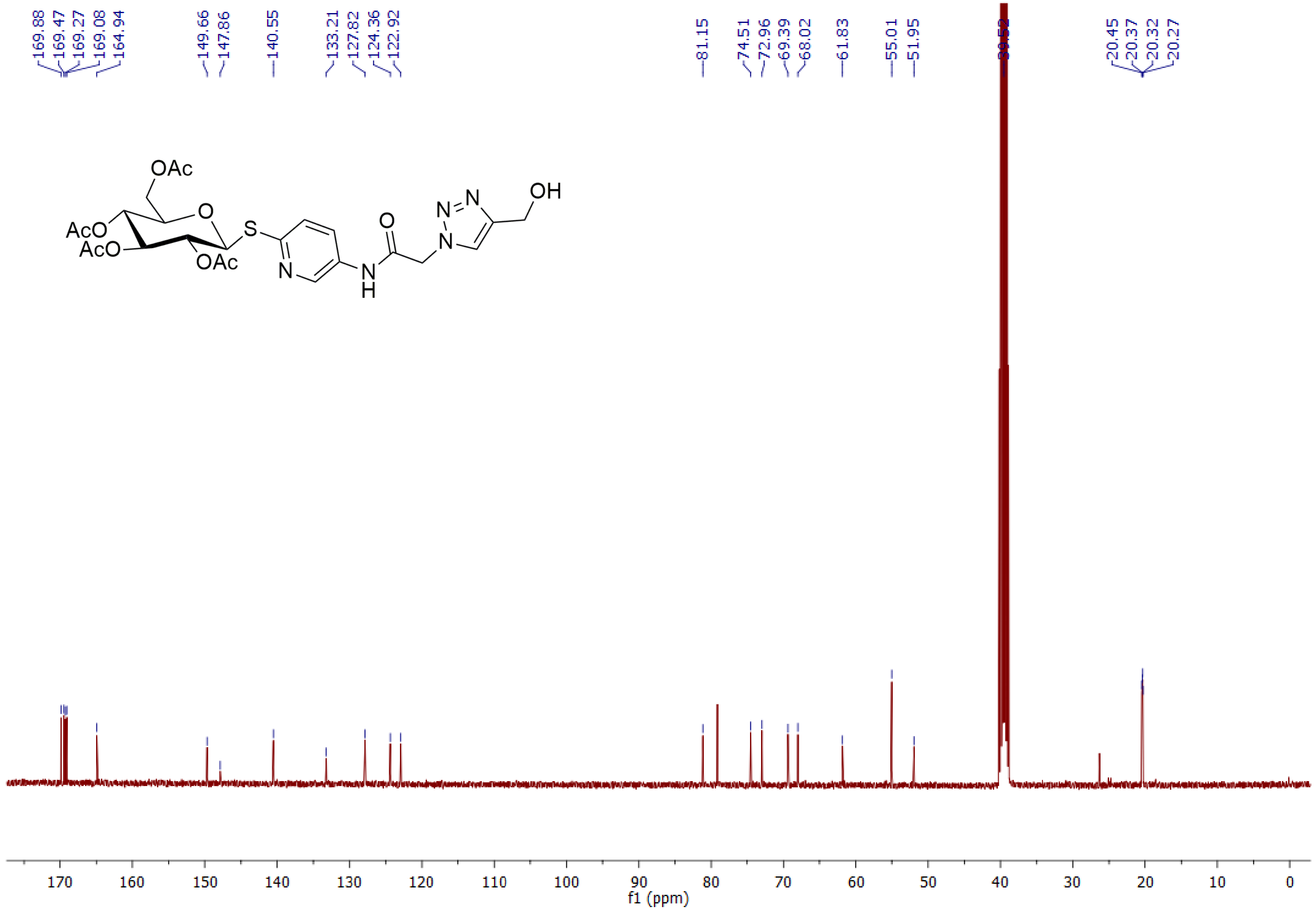

Figure S42. ^{13}C NMR spectrum of compound **M21** (100 MHz/DMSO/TMS; δ (ppm)).

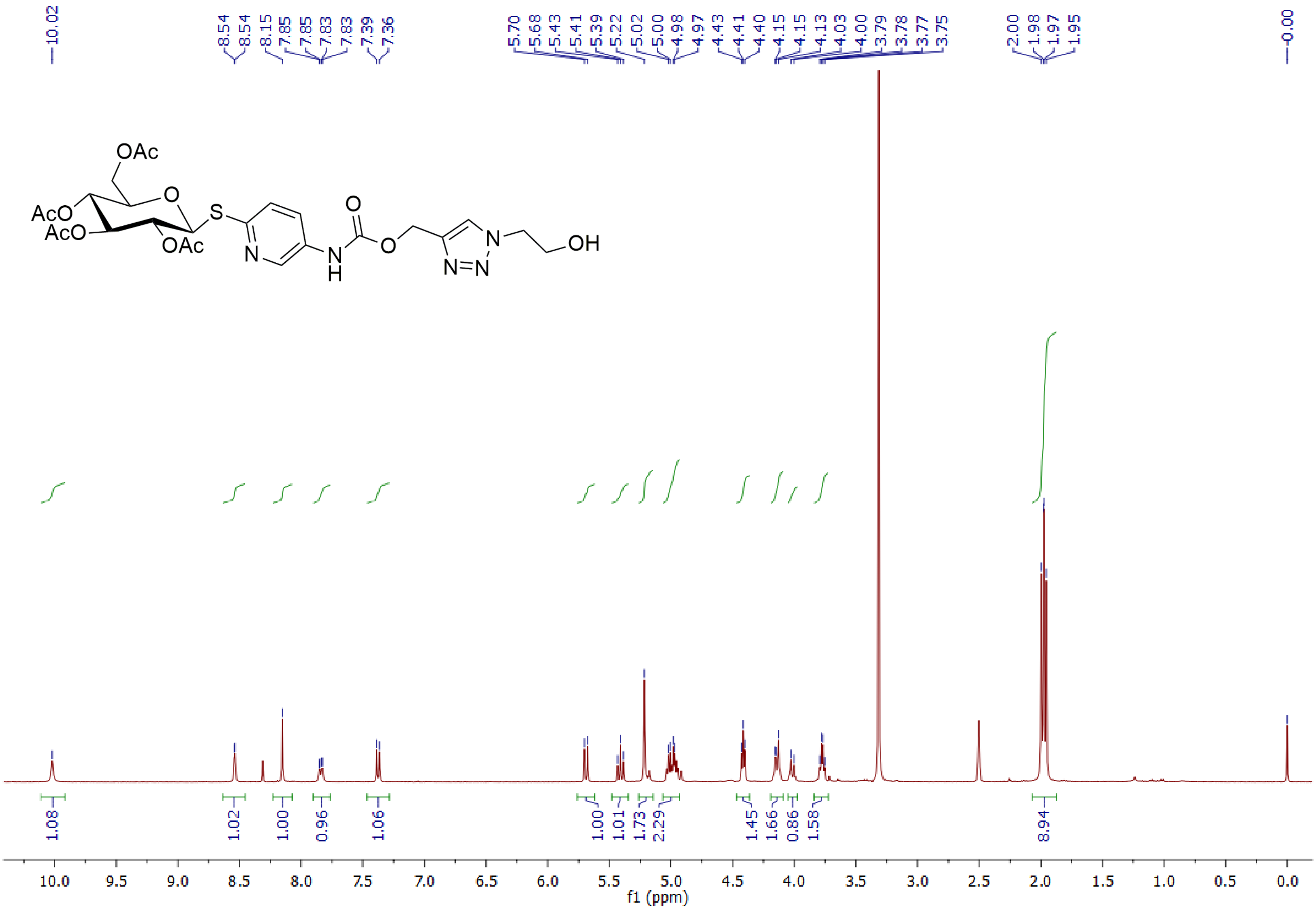

Figure S43. ^{1}H NMR spectrum of compound **M22** (400 MHz/DMSO/TMS; δ (ppm)).

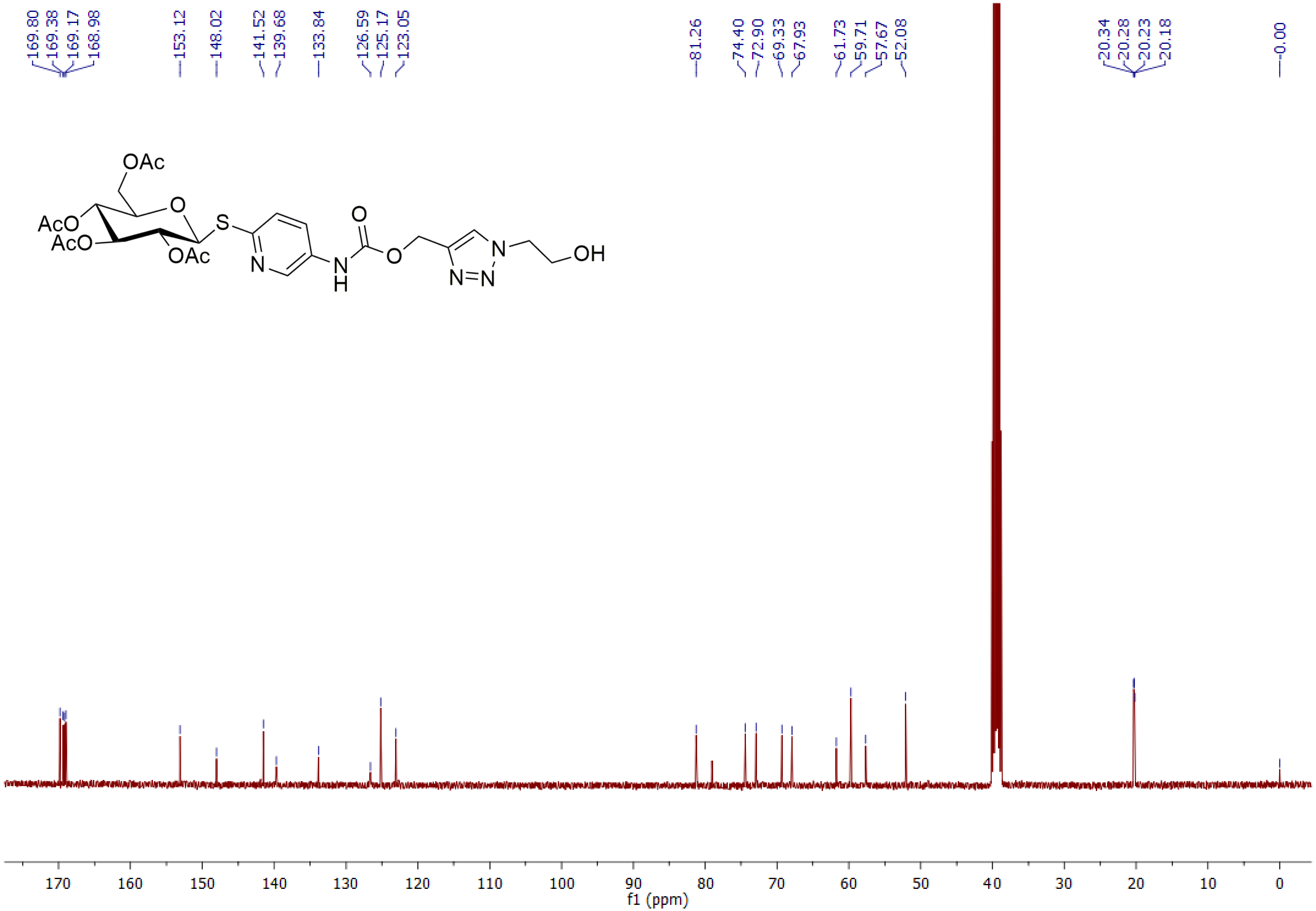

Figure S44. ^{13}C NMR spectrum of compound **M22** (100 MHz/DMSO/TMS; δ (ppm)).

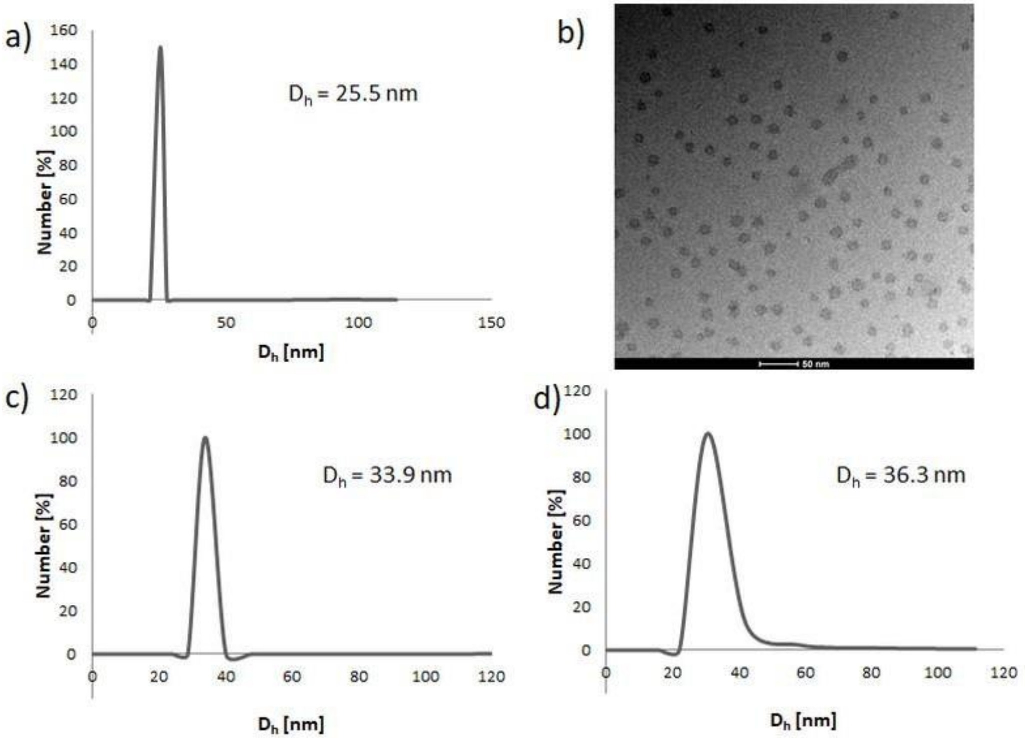

Figure S45. The size distributions of blank micelles (a), **M5**-loaded micelles (c), **M7**-loaded micelles (d); cyro-TEM image of blank micelles (b).
